# Supplementary material for: Generative AI designs functional thiolation domains for reprogramming non-ribosomal peptide synthetases
Source: Nat Commun. 2026 Sep 22;17:10084. doi: 10.1038/s41467-026-77963-6 (PMC13598123; doi:10.1038/s41467-026-77963-6)
Supplement: Supplementary file 1 [file 41467_2026_77963_MOESM1_ESM.pdf]

Generative AI designs functional thiolation domains for reprogramming non-ribosomal peptide synthetases

Authors:

Emre F. Bülbül[1,†], Seounggun Bang[1,†], Kevin George[2,†], Gabriele Bianchi[1,3], Prateek Raj[4], Seonyong Chung[1], Vincent Pauline[5], Ramon Hochstrasser[6,7], Hannah A. Minas[6,7], Walid A. M. Elgaher[1,3,8], Andreas M. Kany[1,3], Anna K. H. Hirsch[1,3,8], Steven Schmitt[6], Dirk W. Heinz[4], Olga V. Kalinina[1,2,9], Dietrich Klakow[10], Kenan A. J. Bozhüyük[1,6,*]

Affiliations:

1 Helmholtz Institute for Pharmaceutical Research Saarland (HIPS), Helmholtz Centre for Infection Research (HZI), PharmaScienceHub (PSH), Campus E8.1, 66123, Saarbrücken, Germany

2 Center for Bioinformatics, Saarland University, Campus E2.1, 66123, Saarbrücken, Germany

3 Deutsches Zentrum für Infektionsforschung (DZIF), Inhoffenstr. 7, 38124, Braunschweig, Germany

4 Department of Molecular Structural Biology, Helmholtz Centre for Infection Research, Inhoffenstr.7, 38124, Braunschweig, Germany

5 Technical University of Munich - Helmholtz AI - Munich Center for Machine Learning (MCML), Friedrich-Ludwig-Bauer-Str. 5, 85748, Garching b. München, Germany

6 Myria Biosciences AG, Hochbergerstrasse 60C, 4057, Basel, Switzerland

7 Bioprocess Laboratory (BPL), Department of Biosystems Science and Engineering (D-BSSE), Eidgenössische Technische Hochschule (ETH) Zürich, Klingelbergstrasse 48, 4056, Basel, Switzerland

8 Saarland University, Department of Pharmacy, Campus E8.1, 66123, Saarbrücken, Germany

9 Medical Faculty, Saarland University, 66421, Homburg, Germany

10 Spoken Language Systems (LSV), Saarland University, PharmaScienceHub (PSH), Campus C7.1, 66123, Saarbrücken, Germany

† These authors contributed equally.

* Corresponding author: kenan.bozhueyuek@helmholtz-hips.de

Supplementary Information

Contents

Supplementary Table 1. ESI-MS data of biosynthesized peptides, including peptide codes, calculated m/z values, molecular formulas, and literature references1–4 3

Supplementary Table 2. LC-ESI-MS/MS conditions and parameters for reaction monitoring of **1,2, 6**, and diphenhydramine (**DPH**)........ 4

Supplementary Table 3. Quantification of interface contact persistence across catalytic states. 4

Supplementary Table 4. Pairwise sequence identities between WT GxpS module 3 constructs and the corresponding template structures 5

Supplementary Figures **6**

Supplementary Figure 1. Base peak chromatograms (BPC) and extracted ion chromatograms (EIC) of Type S GxpS expressed peptides. 7

Supplementary Figure 2. LC-ESI-MS/MS chromatograms comparing producer extracts to external standards. 8

Supplementary Figure 3. Sequence similarity landscape and functional performance of AI-designed T-domains. 8

Supplementary Figure 4. Performance of error-prone libraries derived from wild-type and AI-designed T-domains. 9

Supplementary Figure 5. SEC profiles of WT GxpS_T3 and AI-2. 9

Supplementary Figure 6. Expression and solubility of tri-domain and di-domain constructs

Supplementary Figure 7. Structural dynamics of the thiolation state in WT and AI-2 constructs. 11

Supplementary Figure 8. Structural dynamics of the condensation donor state in WT and AI-2 constructs. 12

Supplementary Figure 9. Structural dynamics of the condensation acceptor state in WT and AI-2 constructs. 13

Supplementary Figure 10. Phylogenetic analysis of AI-designed and reference T-domains with downstream context. 14

Supplementary Figure 11. a, Base peak chromatograms (BPCs) and extracted ion chromatograms (EICs) of GxhS-1 expressed peptides. 17

Supplementary Figure 12. **a,** Base peak chromatograms (BPCs) and extracted ion chromatograms (EICs) of GshS expressed peptides. 19

Supplementary Figure 13. **a,** Base peak chromatograms (BPCs) and extracted ion chromatograms (EICs) of GxhS-2 expressed peptides. 21

Supplementary Figure 14. RapidFire LC–MS screening of T-domain variants across GxhS-1, GxhS-2, GshS, and SzeS systems. 22

Supplementary Figure 15. Relationship between ESMFold prediction confidence and experimental production. 23

Supplementary Figure 16. State-dependent interdomain interactions of WT GxpS_T3 and AI-2 T-domains across the NRPS catalytic cycle. 24

Supplementary Note 1: AI workflow for generation of T-domains 25

Library generation using generative protein models 25

Supplementary Figure 17. ESM2 embedding of the Round 1 generated pool, illustrating the diversity of the selected AI-designed T-domain variants. 26

Surrogate Models 28

Supplementary Table 5. Mean values and 95% confidence intervals (CI) for Spearman correlation on the T-domain test set. 30

Supplementary Table 6. Mean values and 95% confidence intervals (CI) for spearman correlation on both the GB1 and TEM1 dataset. 30

Supplementary Table 7. Performance on both the GB1 and TEM1 dataset under low-data setting. 31

Generation and filtering of Round 3 candidates 31

Supplementary Methods 33

Supplementary Method 1. Peptide synthesis and purification 33

Supplementary Figure 18. HRMS (ESI+) spectrum and UV chromatogram of **6.** 35

Supplementary Method 2. Biochemical Characterization of AI-2 and WT GxpS_T3 variants 36

Supplementary References 38

Uncropped Gels for Supplementary Figure 6 39

Supplementary Tables

Supplementary Table 1. ESI-MS data of biosynthesized peptides, including peptide codes, calculated m/z values, molecular formulas, and literature references1–4. Construct abbreviations are defined as follows: GxpS SU2, GameXPeptide synthetase (GxpS) subunit 2 expressed alone; GxpS full, co-expression of GxpS subunits 1 and 2; GxhS-1 and GxhS-2, hybrid synthetases assembled from GxpS and xenotetrapeptide synthetase (XtpS) modules (variant 1 or 2, respectively); and GshS, a hybrid synthetase assembled from GxpS and szentiamide synthetase (SzeS) modules.

NRPS system	Peptide	MS calculated $[M+H]^+$	Molecular formula	Compound	Reference
GxpS SU2	**1, 2**	392.2544	$C_{18}H_{35}N_3O_4$	fIL/FIL	[1]
	**3, 4**	358.2700	$C_{21}H_{33}N_3O_4$	lIL/LIL	[1]
GxpS full	**5**	552.4119	$C_{29}H_{53}N_5O_5$	cyclo(vLlIL)	[2]
	**6**	586.3963	$C_{32}H_{51}N_5O_5$	cyclo(vLfIL)	[2]
	**7**	566.4276	$C_{30}H_{55}N_5O_5$	cyclo(lLlIL)	[2]
	**8**	600.4119	$C_{33}H_{53}N_5O_5$	cyclo(lLfIL)	[2]
GxhS-1	**9**	477.3071	$C_{25}H_{40}N_4O_5$	vLfV	This study
	**10**	491.3228	$C_{26}H_{42}N_4O_5$	lLfV	This study
	**11**	505.3384	$C_{27}H_{44}N_4O_5$	vLfV-OEt	This study
	**12**	443.3228	$C_{22}H_{42}N_4O_5$	vLlV	This study
	**13**	457.3384	$C_{23}H_{44}N_4O_5$	lLlV	This study
	**14**	459.2966	$C_{25}H_{38}N_4O_4$	cyclo(vLfV)	[3]
	**15**	425.3122	$C_{22}H_{40}N_4O_4$	cyclo(vLlV)	[3]
GshS	**16**	727.3814	$C_{40}H_{50}N_6O_7$	vLfYW	This study
	**17**	693.3970	$C_{37}H_{52}N_6O_7$	vLlYW	This study
	**18**	741.3970	$C_{41}H_{52}N_6O_7$	lLfYW	This study
	**19**	755.4127	$C_{42}H_{54}N_6O_7$	vLfYW-OEt	This study
	**20**	711.3865	$C_{40}H_{50}N_6O_6$	vLfFW	This study
	**21**	677.4021	$C_{37}H_{52}N_6O_6$	vLlFW	This study
	**22**	739.4178	$C_{42}H_{54}N_6O_6$	vLfFW-OEt	This study
GxhS-2	**23**	411.2966	$C_{21}H_{38}N_4O_4$	cyclo(vLvV)	[3]
	**24**	425.3122	$C_{22}H_{40}N_4O_4$	cyclo(lLvV)	[3]
	**25**	429.3071	$C_{21}H_{40}N_4O_5$	vLvV	This study
	**26**	443.3228	$C_{22}H_{42}N_4O_5$	lLvV	This study
SzeS	**27**	838.4134	$C_{45}H_{55}N_7O_9$	lTfvYW	[4]

Supplementary Table 2. LC-ESI-MS/MS conditions and parameters for reaction monitoring of **1,2, 6**, and diphenhydramine (**DPH**).

Analyte	Q1 mass [Da]	Q3 mass [Da]	Collision Energy [V]	Tube lens offset [V]	Spray Voltage [V]	Retention Time [min]
**1 and 2**	390.217	129.967	21.9	92	3700	1.08 and 1.1
		259.167	18.7			
**6**	586.367	261.133	21.9	79	4000	1.35
		374.217	18.7			
**DPH**	256.05	164.967<br>166.967	41.8<br>13.0	30	4000	1.05

Supplementary Table 3. Quantification of interface contact persistence across catalytic states. Residue–residue contact statistics derived from MD simulations of the thiolation, condensation donor (C_donor), and condensation acceptor (C_acceptor) states for WT GxpS_T3 and AI-2 constructs (two independent replicates each). For each interface (A–T or T–C), ≥50 % occupancy denotes the number of residue–residue contact pairs present in ≥50 % of analyzed MD frames, while ≥80 % occupancy indicates the number of highly persistent contacts present in ≥80 % of frames. Contacts were defined using a heavy-atom distance cutoff of 4.5 Å and evaluated every 10th frame over 100 ns trajectories (1,000 frames analyzed per replicate). Interfaces are defined as follows: **A–T** denotes the interface between the A-domain and the T-domain, whereas **T–C** denotes the interface between the T-domain and the C-domain. Complete interaction data are provided in Supplementary Data 1.

State	Interface	Construct	≥50% occupancy	≥80% occupancy
Thiolation_Rep1	AT	WT	28	19
Thiolation_Rep2	AT	WT	29	16
Thiolation_Rep1	AT	AI-2	26	10
Thiolation_Rep2	AT	AI-2	20	12
C_donor_Rep1	AT	WT	10	6
C_donor_Rep2	AT	WT	10	5
C_donor_Rep1	TC	WT	47	25
C_donor_Rep2	TC	WT	38	21
C_donor_Rep1	AT	AI-2	2	1
C_donor_Rep2	AT	AI-2	13	6
C_donor_Rep1	TC	AI-2	43	23
C_donor_Rep2	TC	AI-2	30	17
C_acceptor_Rep1	AT	WT	16	8
C_acceptor_Rep2	AT	WT	6	2
C_acceptor_Rep1	TC	WT	38	24
C_acceptor_Rep2	TC	WT	52	31
C_acceptor_Rep1	AT	AI-2	4	2
C_acceptor_Rep2	AT	AI-2	4	1
C_acceptor_Rep1	TC	AI-2	28	17
C_acceptor_Rep2	TC	AI-2	43	33

Supplementary Table 4. Pairwise sequence identities between WT GxpS module 3 constructs and the corresponding template structures used for homology modeling of the thiolation, condensation donor, and condensation acceptor states. Sequence identities are reported for the full modeled constructs as well as separately for the A-, T-, and C-domains. Templates correspond to PDB entries 5T3D (thiolation state), 9BE3 (condensation donor state), and 8JBR (condensation acceptor state).

State	Template	Full Construct	T domain	A domain	C domain
**Thiolation**	5T3D	28.4 %	25.3 %	37.8 %	19.5 %
**Condensation donor**	9BE3	31.5 %	27.8 %	43.2 %	19.9 %
**Condensation acceptor**	8JBR	34.8 %	32.9 %	44.1 %	22.4 %

Supplementary Figures

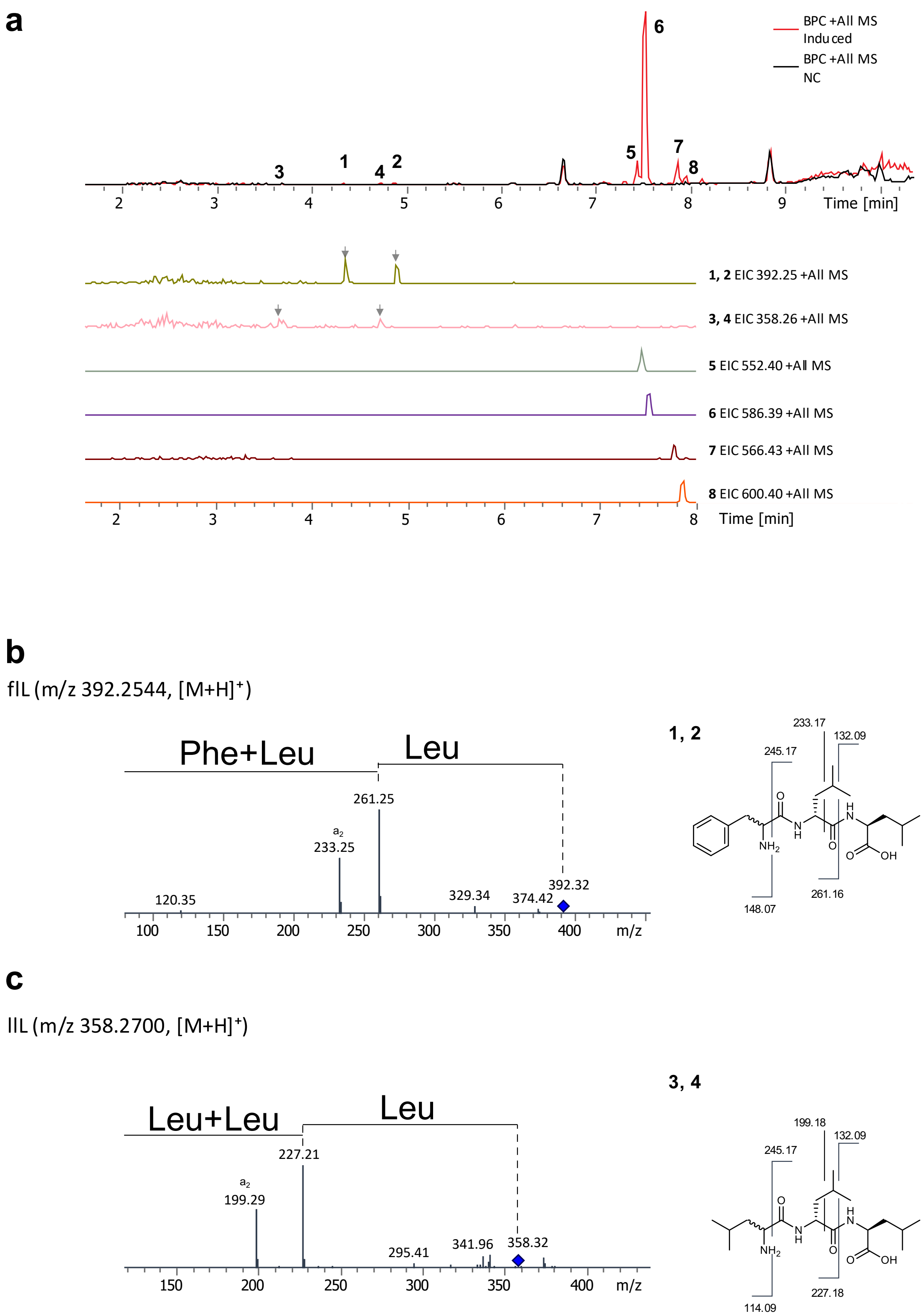

d

vLlIL (m/z 552.4119, [M+H]⁺)

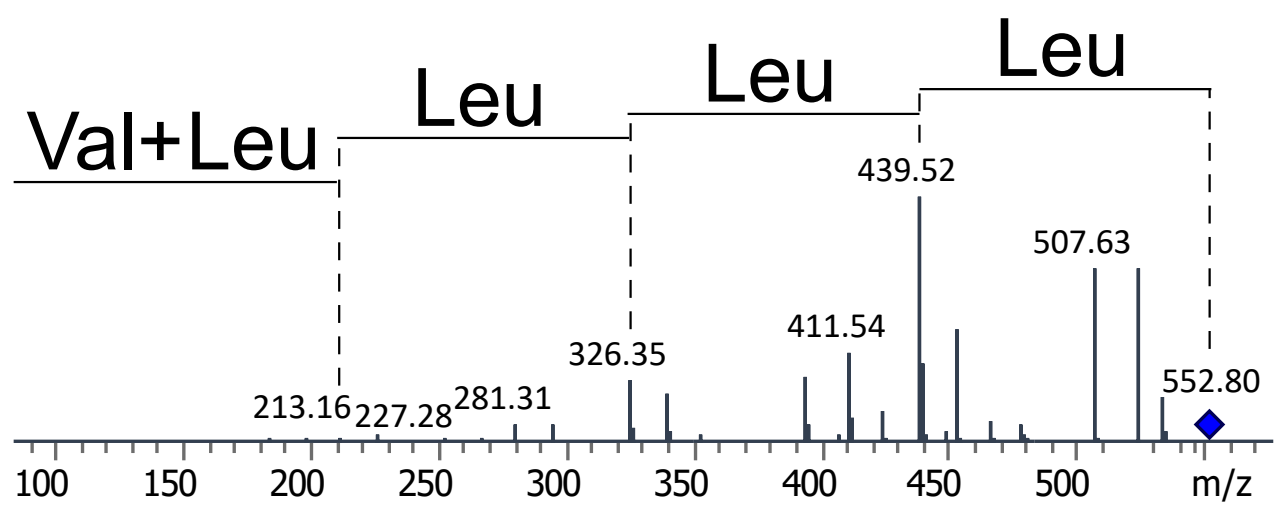

5

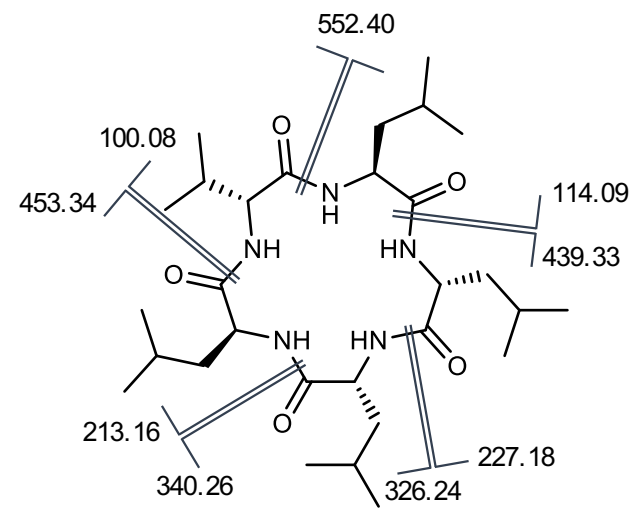

e

vLfIL (m/z 586.3963, [M+H]⁺)

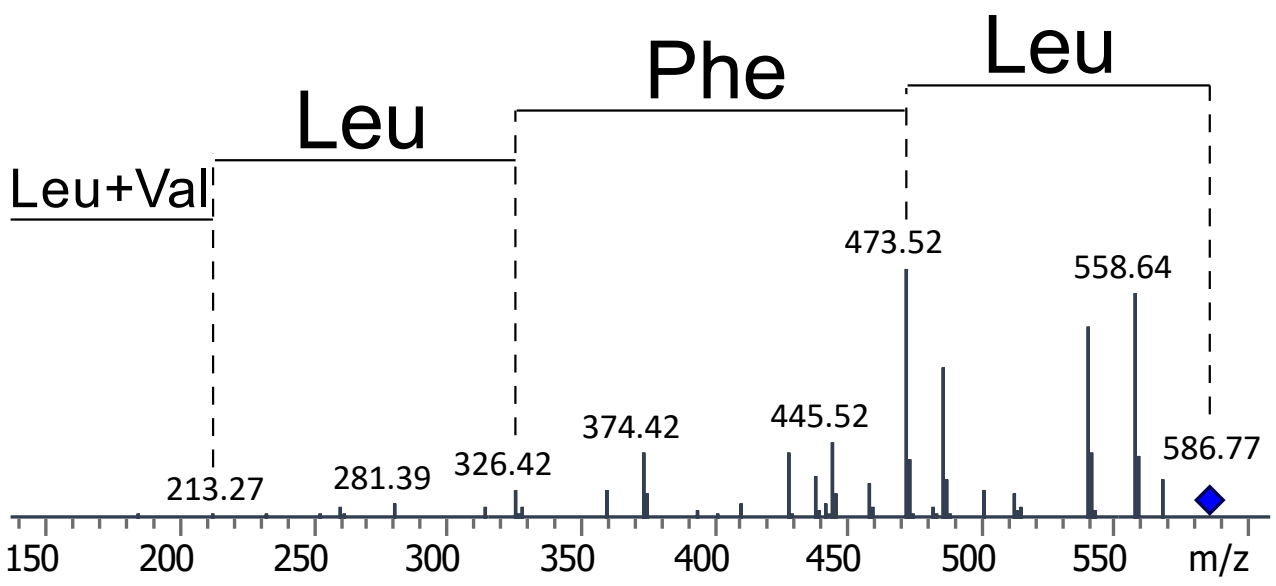

6

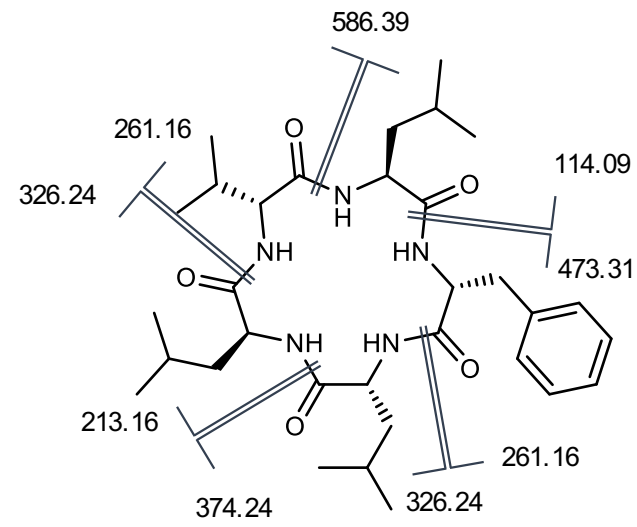

f

ILlIL (m/z 566.4276, [M+H]⁺)

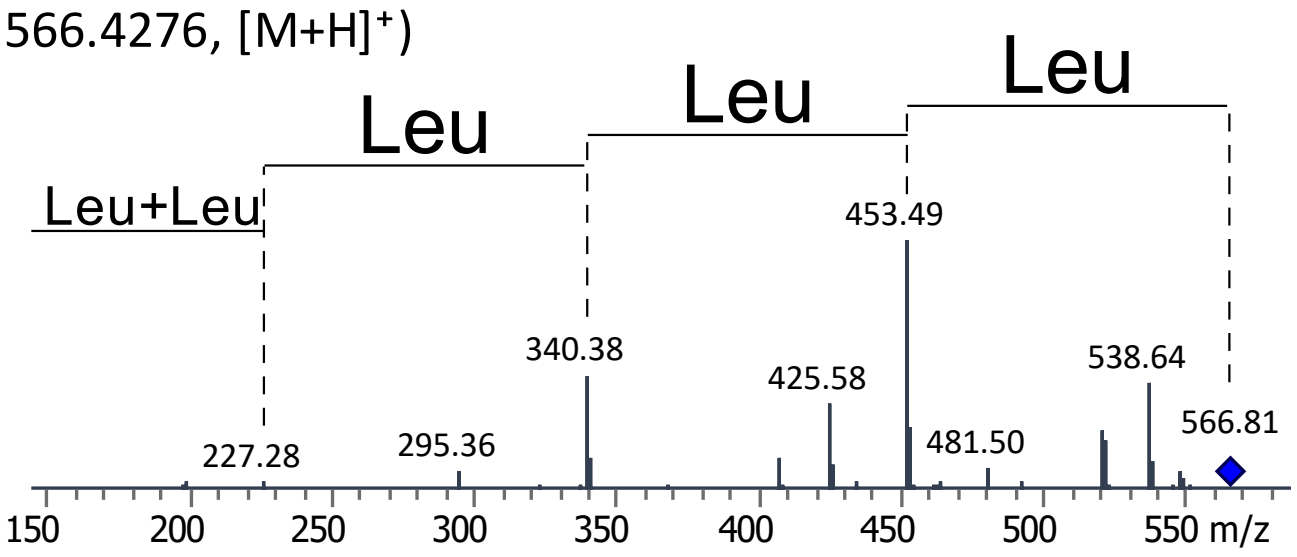

7

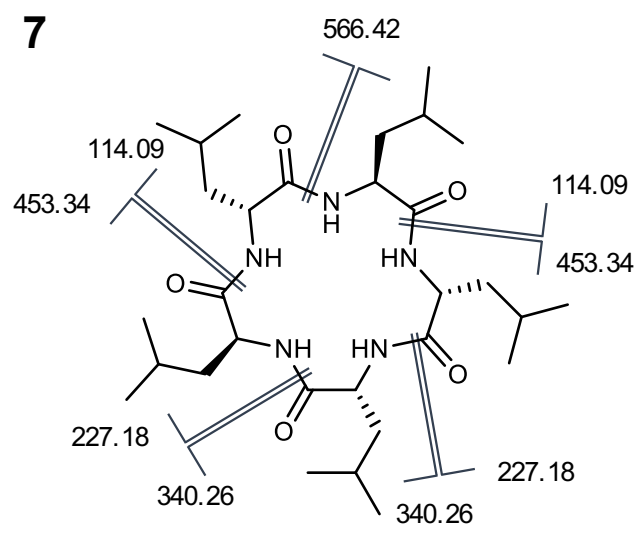

g

ILfIL (m/z 600.4119, [M+H]⁺)

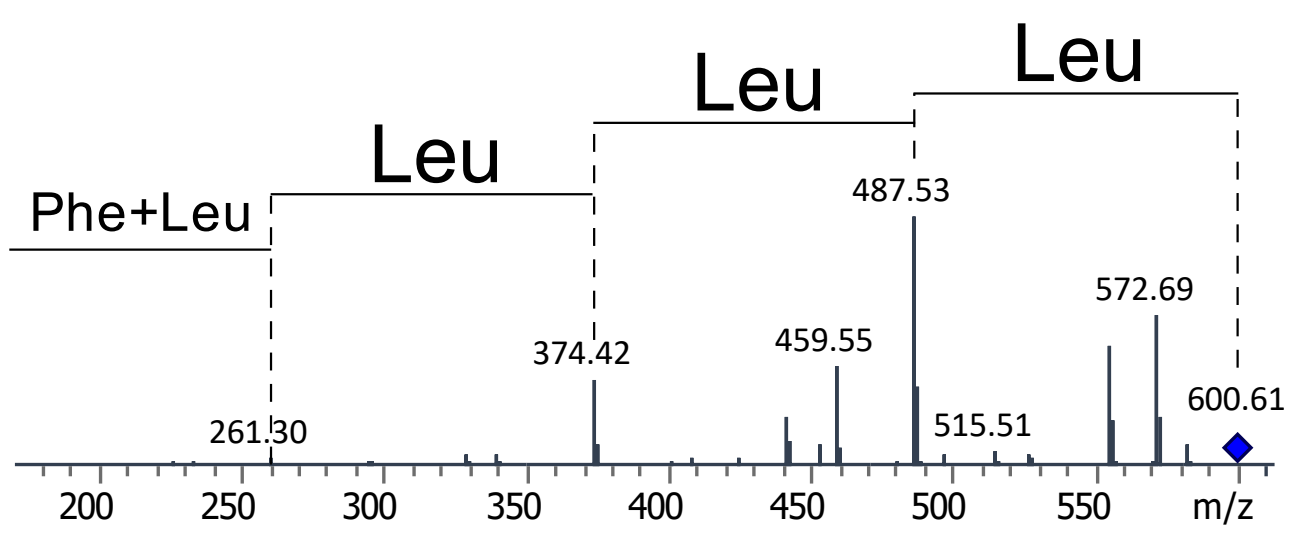

8

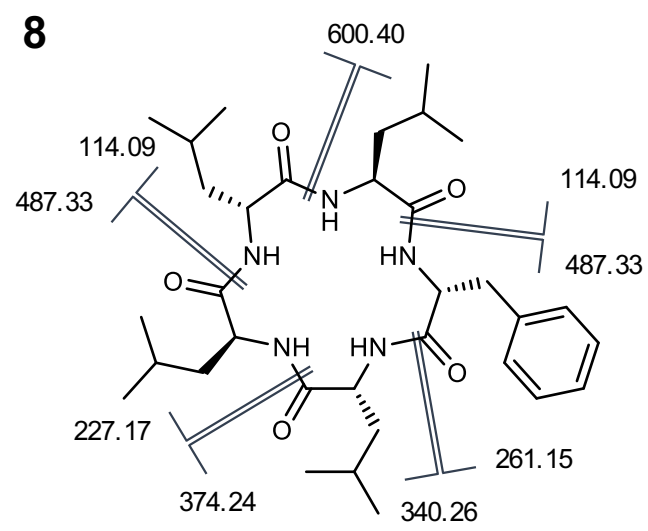

Supplementary Figure 1. Base peak chromatograms (BPC) and extracted ion chromatograms (EIC) of Type S GxpS expressed peptides. **a,** Base peak chromatograms (BPCs) and extracted ion chromatograms (EICs; extracted at the expected m/z values) for peptides produced by GxpS expression. Distinct chromatographic peaks corresponding to individual peptide products are labeled **1–8**. **b–g** Tandem mass spectrometry (MS/MS) spectra of peptides **1–8** with annotated fragment ions, confirming product identity.

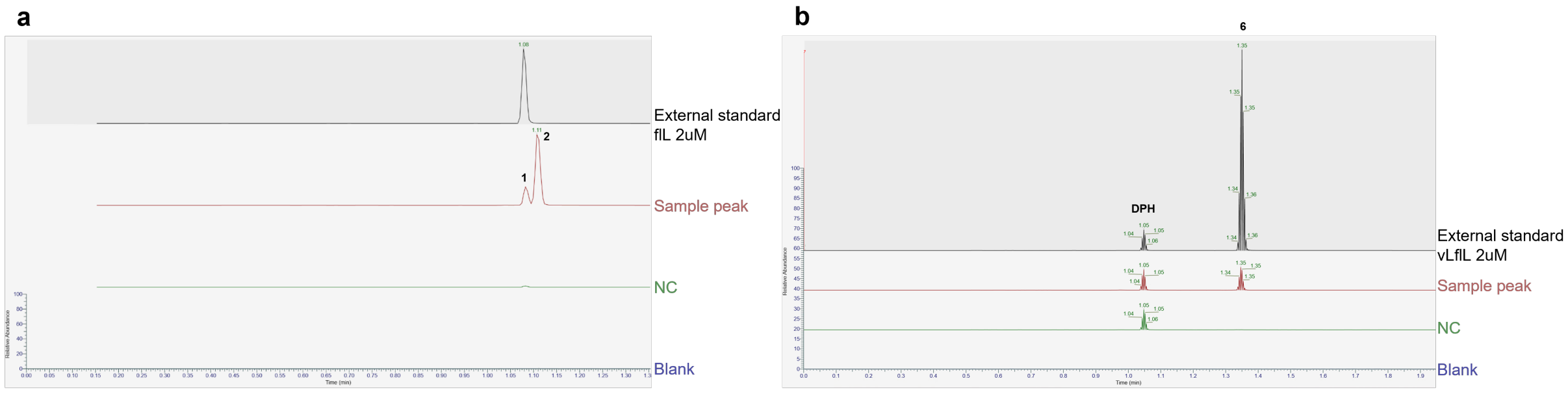

Supplementary Figure 2. LC-ESI-MS/MS chromatograms comparing producer extracts to external standards. **a**, EICs for tripeptides **1** and **2**. The SU-2 only construct produced two major peaks corresponding to a mixture of linear tripeptides products[1]. **b,** EICs for pentapeptide **6.** Traces show the 2mM external standard (black), producer sample (red), negative control containing diphenhydramine (DPH; in **b**), and blank (blue). Distinct epimers were distinguished based on their retention times[1]. LC-ESI-MS/MS conditions and parameters are listed in Supplementary Table 2.

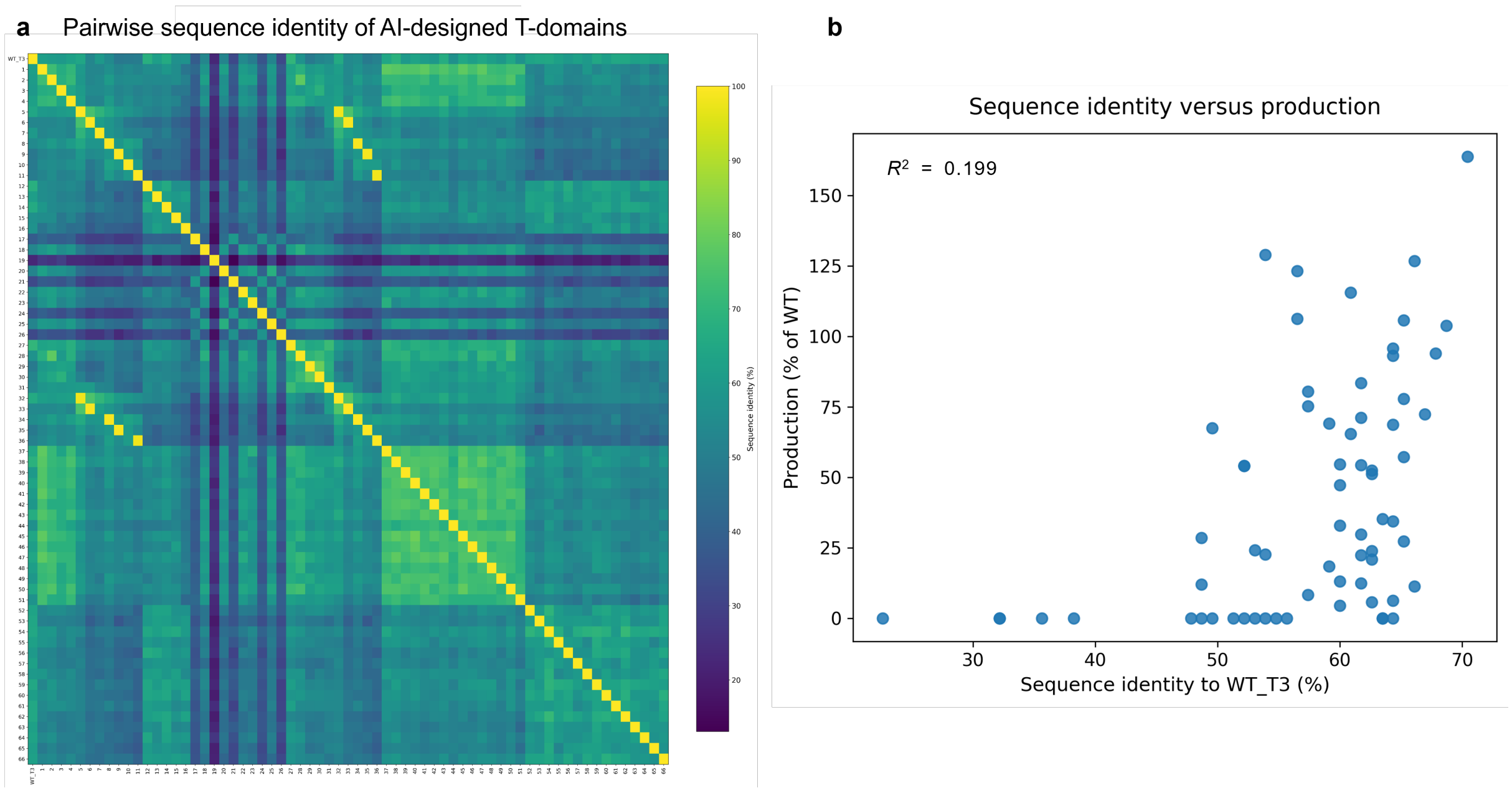

Supplementary Figure 3. Sequence similarity landscape and functional performance of AI-designed T-domains. **a,** Pairwise amino acid sequence identity heatmap of the WT GxpS_T3 and 66 AI-designed T-domain variants. Sequence identity values were calculated by global pairwise alignment and are shown as percentages. Variants are ordered consistently along both axes to facilitate comparison across designs. **b,** Relationship between sequence identity to WT GxpS_T3 and peptide production levels for AI-designed T-domain variants in the SU2-only tripeptide assay system (R^2 = 0.199; Pearson r = 0.45, two-sided p = 1.7×10^{-4}, n = 66 T-domain variants). Production is reported as normalized titers relative to the WT GxpS_T3 control. The WT GxpS_T3 sequence itself is excluded from this plot. Each point represents an individual T-domain variant. The lack of a strict correlation highlights that high catalytic performance can be achieved despite substantial sequence divergence from the native T-domain. Source data are provided as a Source Data file.

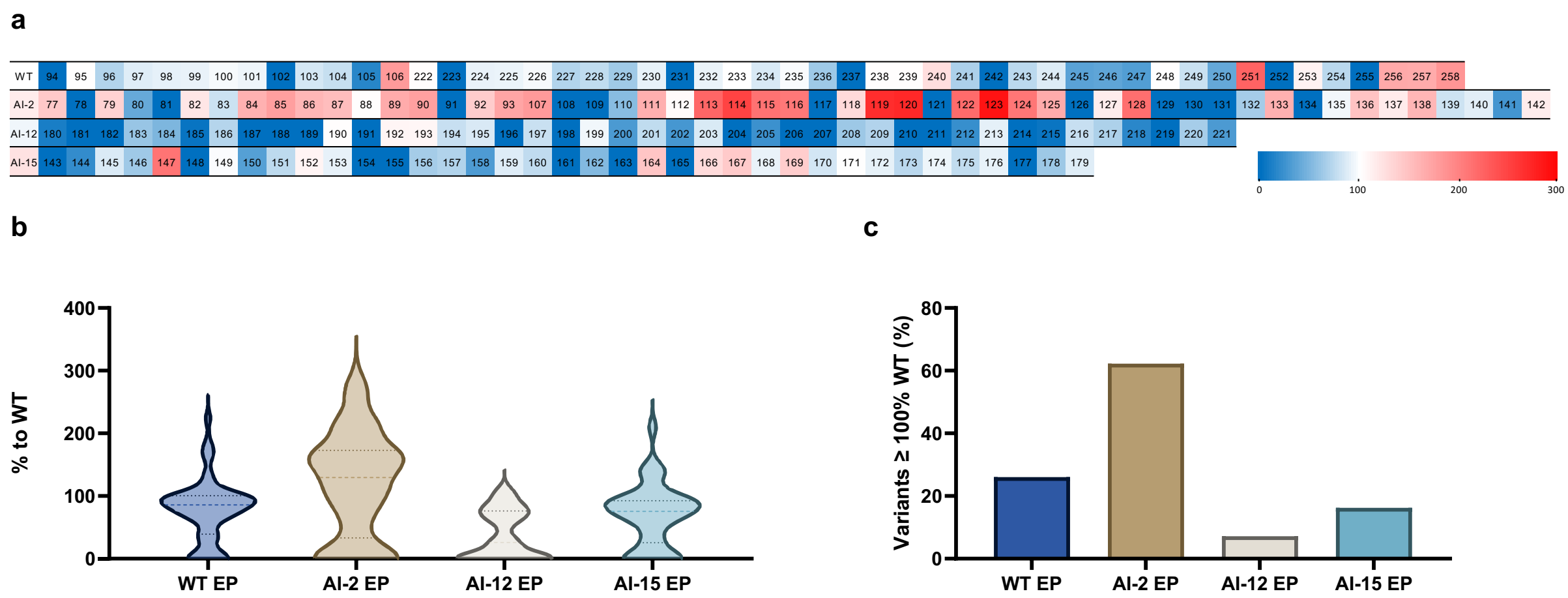

Supplementary Figure 4. Performance of error-prone libraries derived from wild-type and AI-designed T-domains. **a**, Heatmap representation of normalized tripeptide production levels for individual variants from the WT GxpS_T3, AI-2, AI-12, and AI-15 error-prone libraries. Each cell represents an individual variant, and the numbers shown correspond to variant IDs. The color scale represents normalized production values ranging from 0 to 300, with blue indicating lower and red indicating higher production levels. b, Violin plots of normalized peptide production levels for error-prone variants derived from each parental T-domain group. Within each violin, the dashed line indicates the median and the dotted lines the 25th and 75th percentiles; the violin spans the full range of the observed values. Sample sizes are as in c. **c**, Fraction of variants with production ≥ WT, defined as variants exceeding 100 % of WT normalized production. Sample sizes were as follows: WT EP (*n* = 50), AI-2 EP (*n* = 53), AI-12 EP (*n* = 42), AI-15 EP (*n* = 37). Source data are provided as a Source Data file.

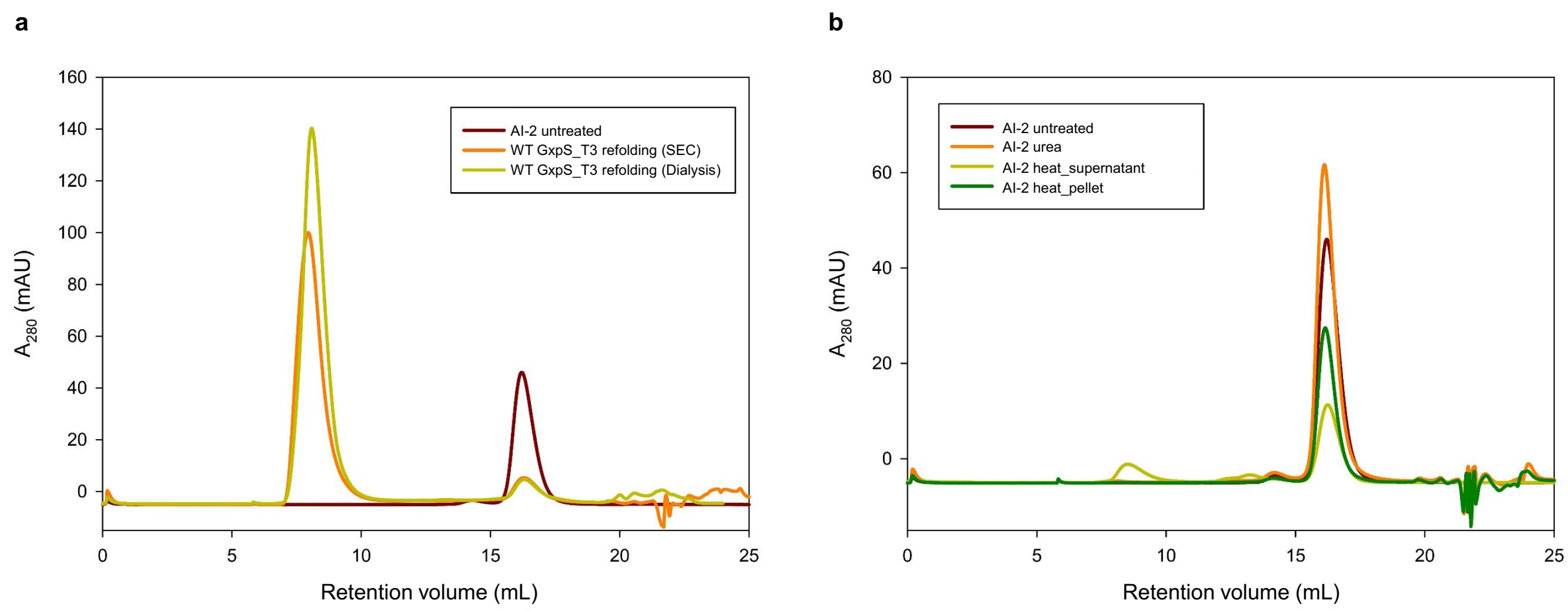

Supplementary Figure 5. SEC profiles of WT GxpS_T3 and AI-2. a, SEC chromatograms of WT GxpS_T3 following refolding after purification under denaturing conditions, showing a major void-volume peak and a minor species at the elution volume corresponding to the soluble reference. b, SEC chromatograms of AI-2 after denaturation in 6 M urea and refolding by SEC, compared to untreated soluble AI-2. Additional traces show SEC profiles of the soluble fraction after heat treatment (95 °C, 10 min; rapid cooling) and of the clarified insoluble fraction. UV absorbance at 280 nm is plotted against retention volume. The experiments were performed in three independent biological replicates (*n* = 3) with similar results. Source data are provided as a Source Data file.

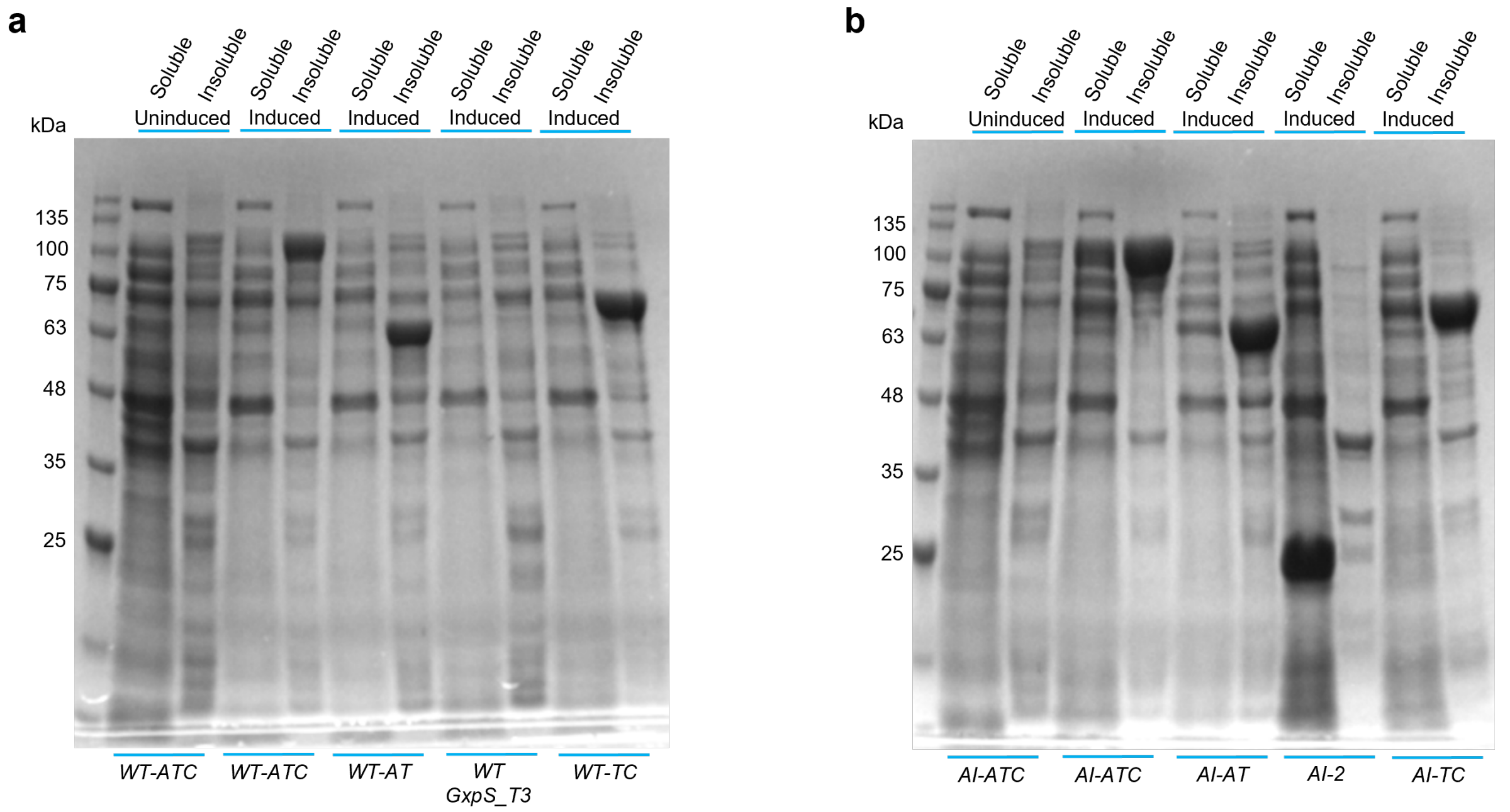

Supplementary Figure 6. Expression and solubility of tri-domain and di-domain constructs containing **(a)** WT GxpS_T3 and **(b)** AI-2. Protein expression was induced by addition of 0.2 mM IPTG followed by incubation at 18 °C for 16 hours. Expected protein sizes were as follows: WT-ATC, 116 kDa; WT-AT, 65 kDa; WT GxpS_T3, 21 kDa; WT-TC, 72 kDa; AI-ATC, 116 kDa; AI-AT, 65 kDa; AI-2, 23 kDa; and AI-TC, 72 kDa. The experiment was performed in two independent biological replicates (n = 2) with similar results. Uncropped gel images are provided at the end of the Supplementary Information file.

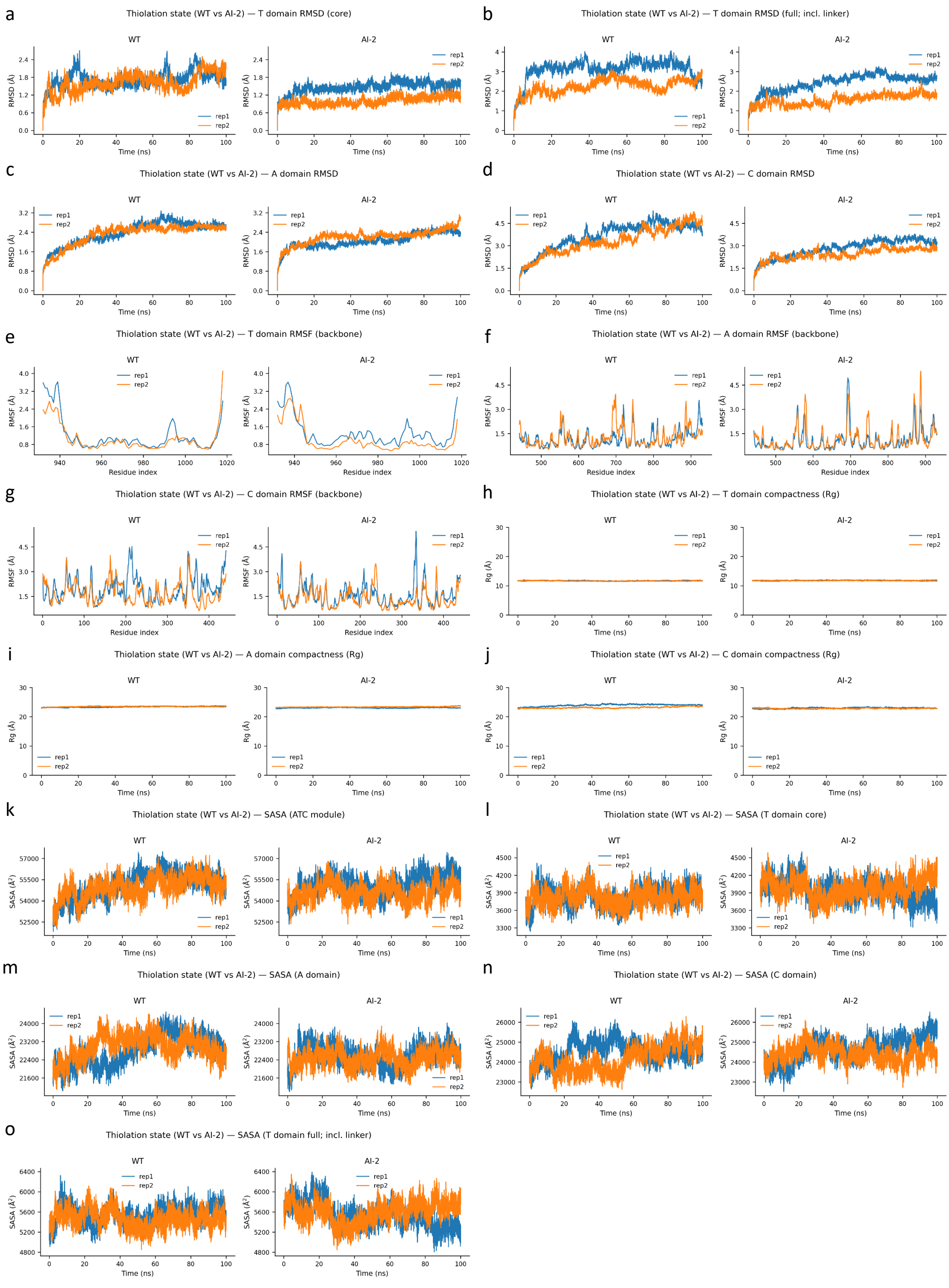

Supplementary Figure 7. Structural dynamics of the thiolation state in WT and AI-2 constructs. Molecular dynamics trajectories (100 ns) of the thiolation state were analyzed for the wild-type (WT) and AI-2 variants using two independent replicates per system (n = 2). Left panels show WT and right panels show AI-2. a: T-domain RMSD without linker. b: T-domain RMSD including linker. c: RMSD of the A-domain. d: RMSD of the C-domain. e: RMSF of the T-domain. f: RMSF of the A-domain. g: RMSF of the C-domain. h: Rg of the T-domain. i: Rg of the A-domain. j: Rg of the C-domain. k: SASA of the ATC tridomain. l: SASA of the T-domain without linker. m: SASA of the A-domain. n: SASA of the C-domain. o: SASA of the T-domain including linker. Time-resolved RMSD are reported for the A-domain, C-domain, T-domain core and full T-domain including the linker, indicating overall structural stability. RMSF of backbone atoms highlight residue-level flexibility across the three domains. Rg profiles for each domain reflect changes in global compactness during the simulation. SASA is shown for the ATC module, individual domains and the T-domain core (and full T-domain where indicated), reporting exposure to solvent over time. For time-dependent analyses, 10,000 frames correspond to 100 ns. WT and AI-2 panels share identical y-axis scaling to enable direct quantitative comparison. No averaging was performed; individual replicate trajectories are shown. Source data are provided as a Source Data file.

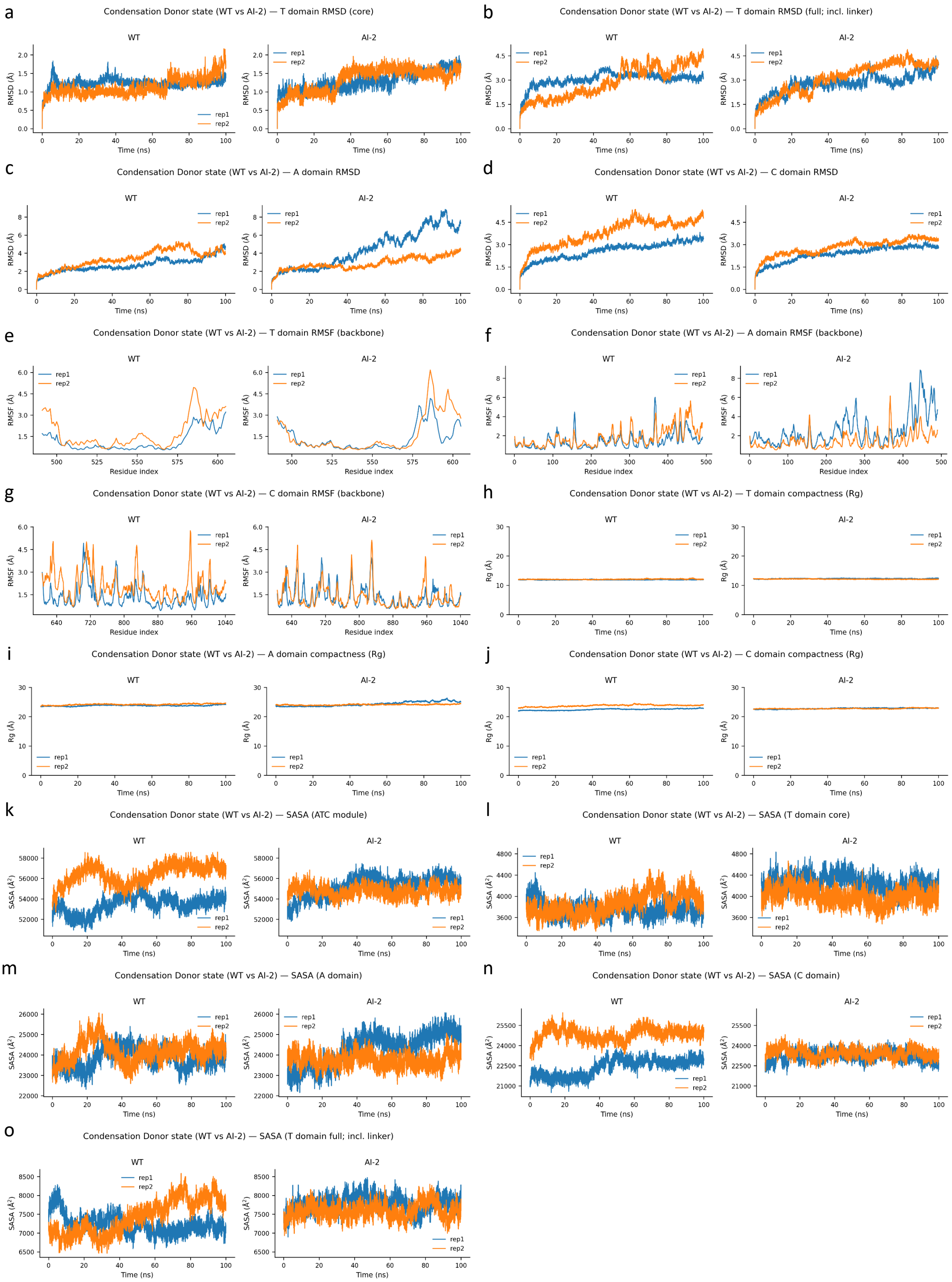

Supplementary Figure 8. Structural dynamics of the condensation donor state in WT and AI-2 constructs. Molecular dynamics trajectories (100 ns) of the condensation donor state were analyzed for the wild-type (WT) and AI-2 variants using two independent replicates per system (n = 2). Left panels show WT and right panels show AI-2. a: T-domain RMSD without linker. b: T-domain RMSD including linker. c: RMSD of the A-domain. d: RMSD of the C-domain. e: RMSF of the T-domain. f: RMSF of the A-domain. g: RMSF of the C-domain. h: Rg of the T-domain. i: Rg of the A-domain. j: Rg of the C-domain. k: SASA of the ATC tridomain. l: SASA of the T-domain without linker. m: SASA of the A-domain. n: SASA of the C-domain. o: SASA of the T-domain including linker. Time-resolved RMSD are reported for the A-domain, C-domain, T-domain core and full T-domain including the linker, indicating overall structural stability. RMSF of backbone atoms highlight residue-level flexibility across the three domains. Rg profiles for each domain reflect changes in global compactness during the simulation. SASA is shown for the ATC module, individual domains and the T-domain core (and full T-domain where indicated), reporting exposure to solvent over time. For time-dependent analyses, 10,000 frames correspond to 100 ns. WT and AI-2 panels share identical y-axis scaling to enable direct

quantitative comparison. No averaging was performed; individual replicate trajectories are shown. Source data are provided as a Source Data file.

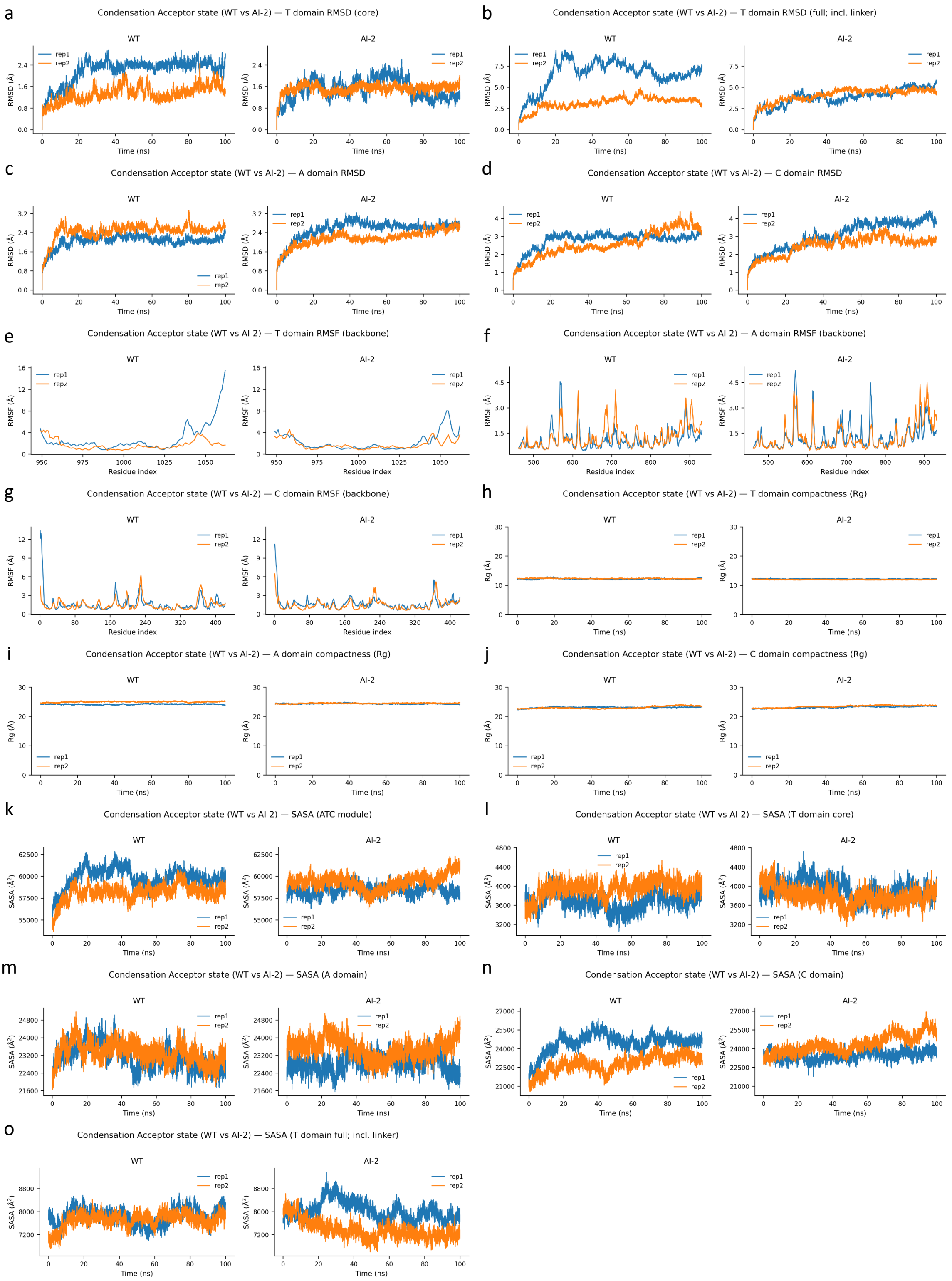

Supplementary Figure 9. Structural dynamics of the condensation acceptor state in WT and AI-2 constructs. Molecular dynamics trajectories (100 ns) of the condensation acceptor state were analyzed for the wild-type (WT) and AI-2 variants using two independent replicates per system (n = 2). Left panels show WT and right panels show AI-2. a: T-domain RMSD without linker. b: T-domain RMSD including linker. c: RMSD of the A-domain. d: RMSD of the C-domain. e: RMSF of the T-domain. f: RMSF of the A-domain. g: RMSF of the C-domain. h: Rg of the T-domain. i: Rg of the A-domain. j: Rg of the C-domain. k: SASA of the ATC tridomain. l: SASA of the T-domain without linker. m: SASA of the A-domain. n: SASA of the C-domain. o: SASA of the T-domain including linker. Time-resolved RMSD are reported for the A-domain, C-domain, T-domain core and full T-domain including the linker, indicating overall structural stability. RMSF of backbone atoms highlight residue-level flexibility across the three domains. Rg profiles for each domain reflect changes in global compactness during the simulation. SASA is shown for the ATC module,

individual domains and the T-domain core (and full T-domain where indicated), reporting exposure to solvent over time. For time-dependent analyses, 10,000 frames correspond to 100 ns. WT and AI-2 panels share identical y-axis scaling to enable direct quantitative comparison. No averaging was performed; individual replicate trajectories are shown. Source data are provided as a Source Data file.

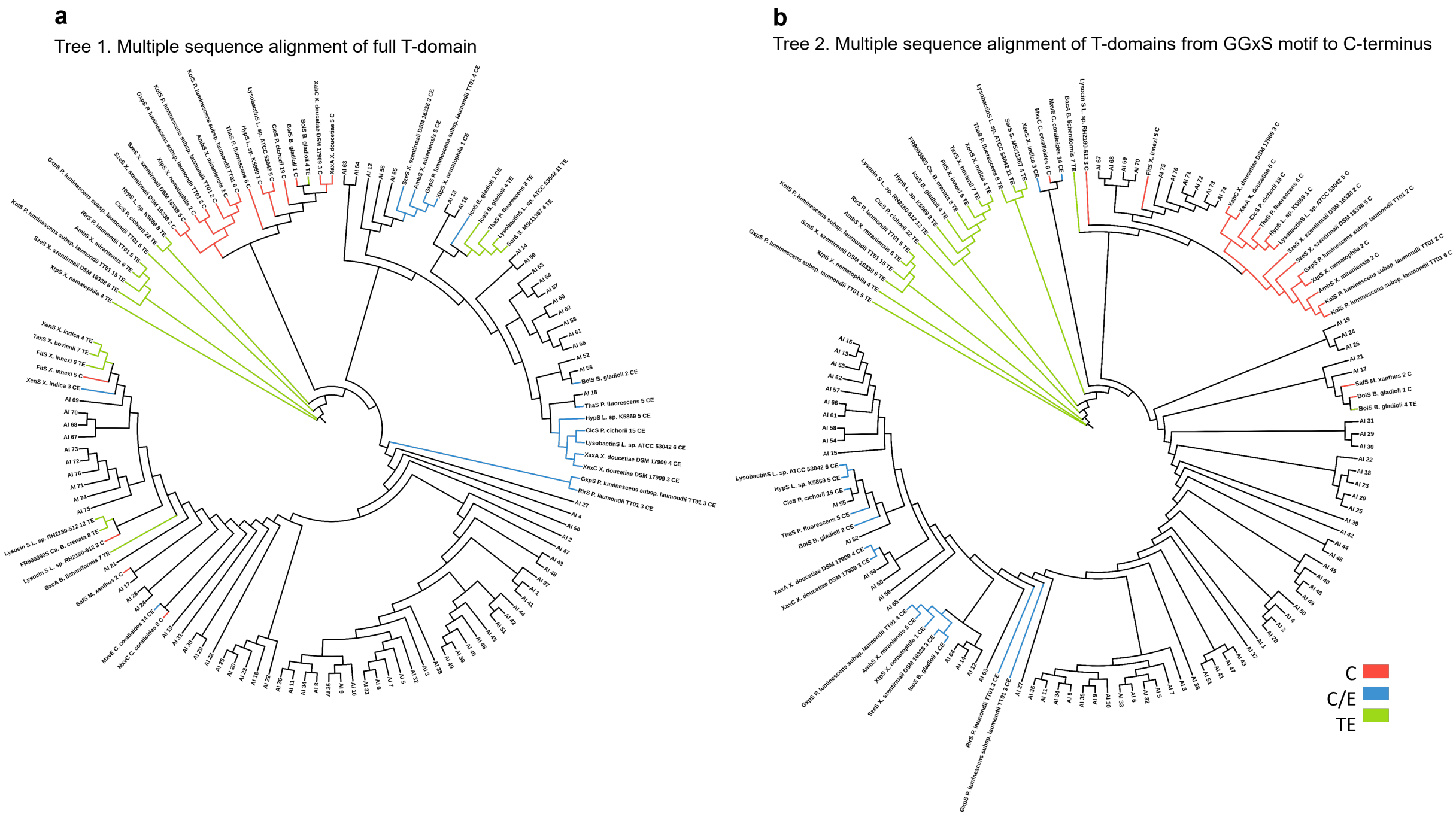

Supplementary Figure 10. Phylogenetic analysis of AI-designed and reference T-domains with downstream context. **a**, Tree 1 inferred from the full-length T-domain. **b**, Tree 2 inferred from the alignment positions beginning at the conserved GGXS motif (within FFXXGGXS sequence) to the C terminus, corresponding to the downstream-facing region of the T-domain. AI-generated T-domain sequences are labeled as AI-n, where n denotes the variant identifier assigned during the design workflow, whereas native NRPS sequences are annotated with the NRPS name, taxon name, module number, and the downstream domain. T-domains are color-coded by the downstream domain type: C (red), C/E (blue), and TE (green). The T-domain sequences used for both trees are provided in Supplementary Data 1. Source data are provided as a Source Data file.

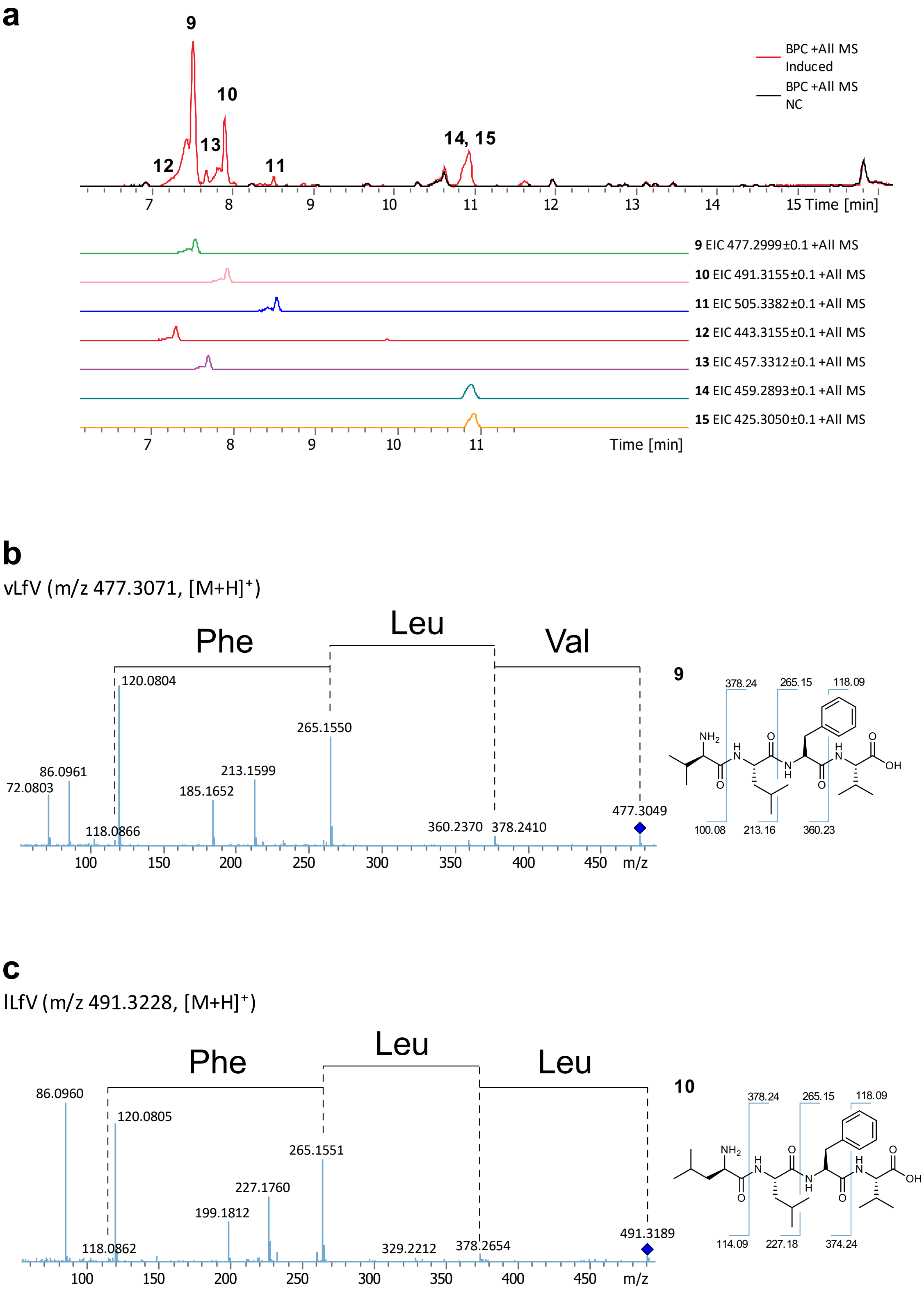
a
9
10
13
12
11
14, 15
BPC +All MS Induced
BPC +All MS NC
7 8 9 10 11 12 13 14 15 Time [min]
9 EIC 477.2999±0.1 +All MS
10 EIC 491.3155±0.1 +All MS
11 EIC 505.3382±0.1 +All MS
12 EIC 443.3155±0.1 +All MS
13 EIC 457.3312±0.1 +All MS
14 EIC 459.2893±0.1 +All MS
15 EIC 425.3050±0.1 +All MS
7 8 9 10 11 Time [min]
b
vLfV (m/z 477.3071, [M+H]+)
Phe
Leu
Val
120.0804
265.1550
86.0961
72.0803
213.1599
185.1652
118.0866
360.2370
378.2410
477.3049
100 150 200 250 300 350 400 450 m/z
9
378.24
265.15
118.09
100.08
213.16
360.23
c
lLfV (m/z 491.3228, [M+H]+)
Phe
Leu
Leu
86.0960
120.0805
265.1551
227.1760
199.1812
118.0862
329.2212
378.2654
491.3189
100 150 200 250 300 350 400 450 m/z
10
378.24
265.15
118.09
114.09
227.18
374.24

d

vLfV-OEt (m/z 505.3384, [M+H]+)

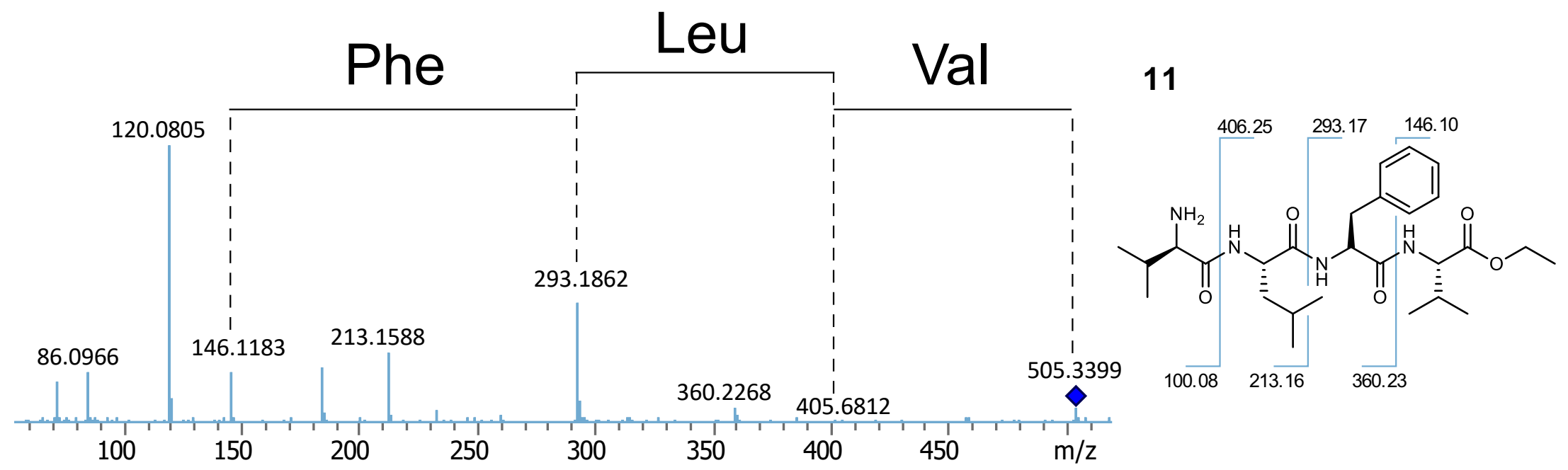

e

vLlV (m/z 443.3228, [M+H]+)

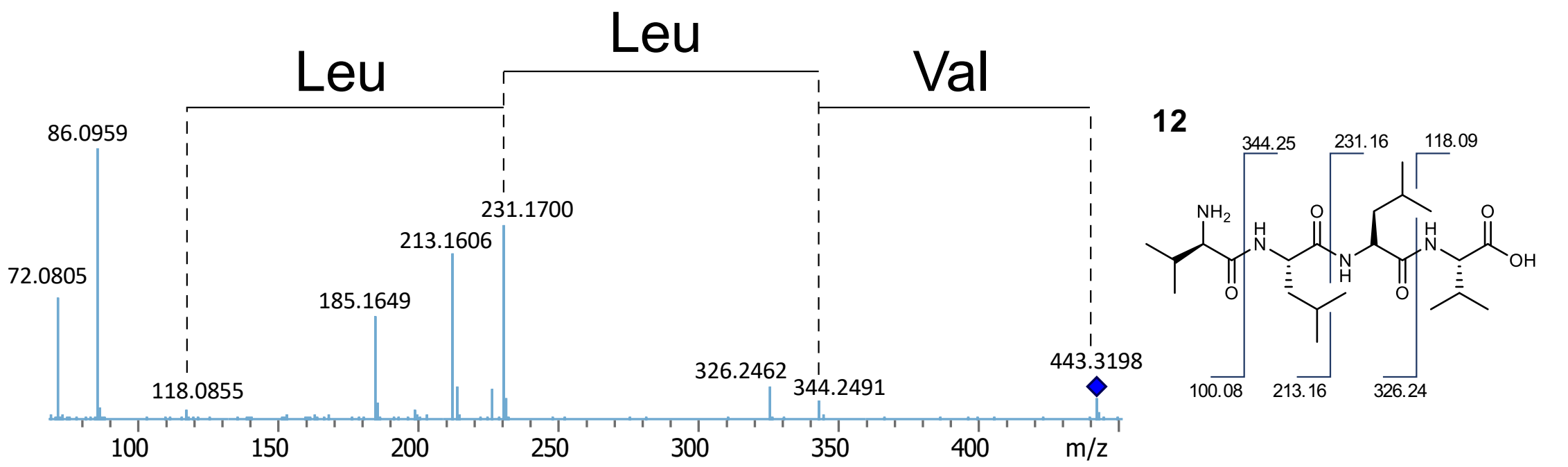

f

lLlV (m/z 457.3384, [M+H]+)

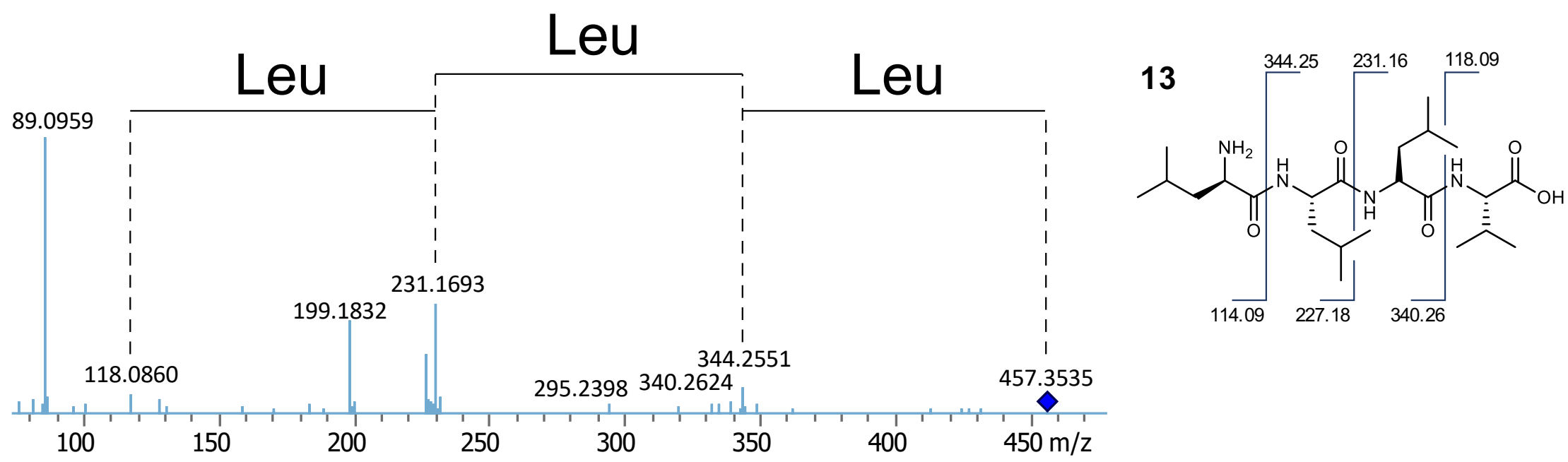

g

cyclo vLfV (m/z 459.2966, [M+H]⁺)

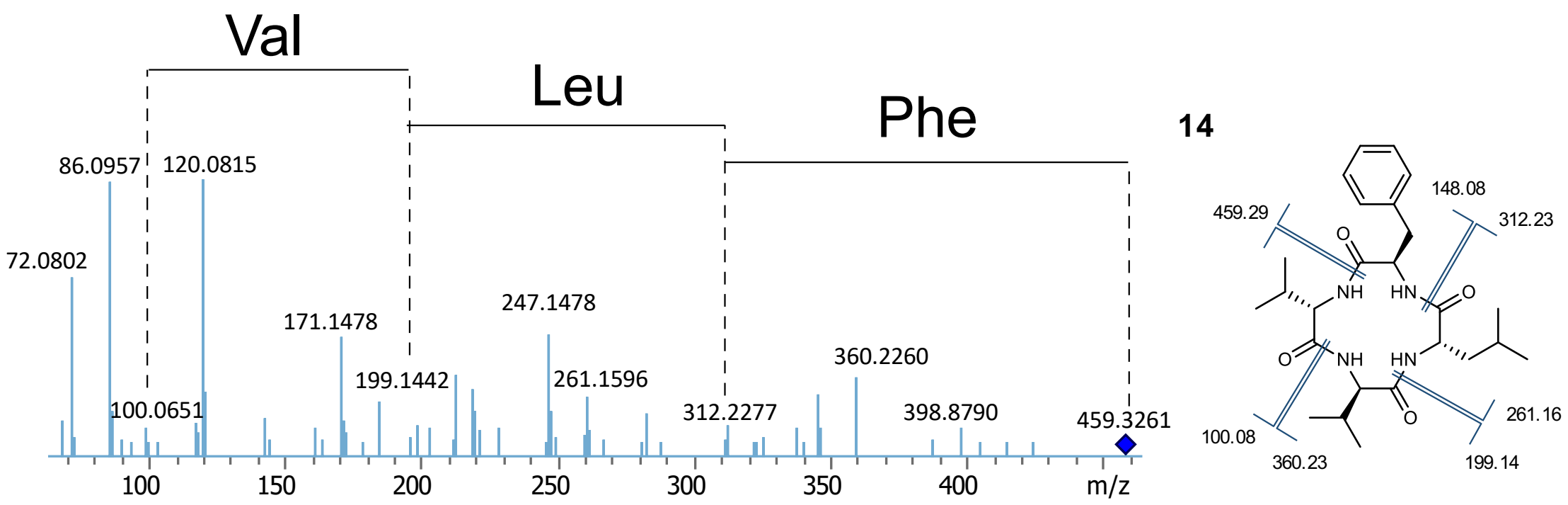

h

cyclo vLlV (m/z 425.3122, [M+H]⁺)

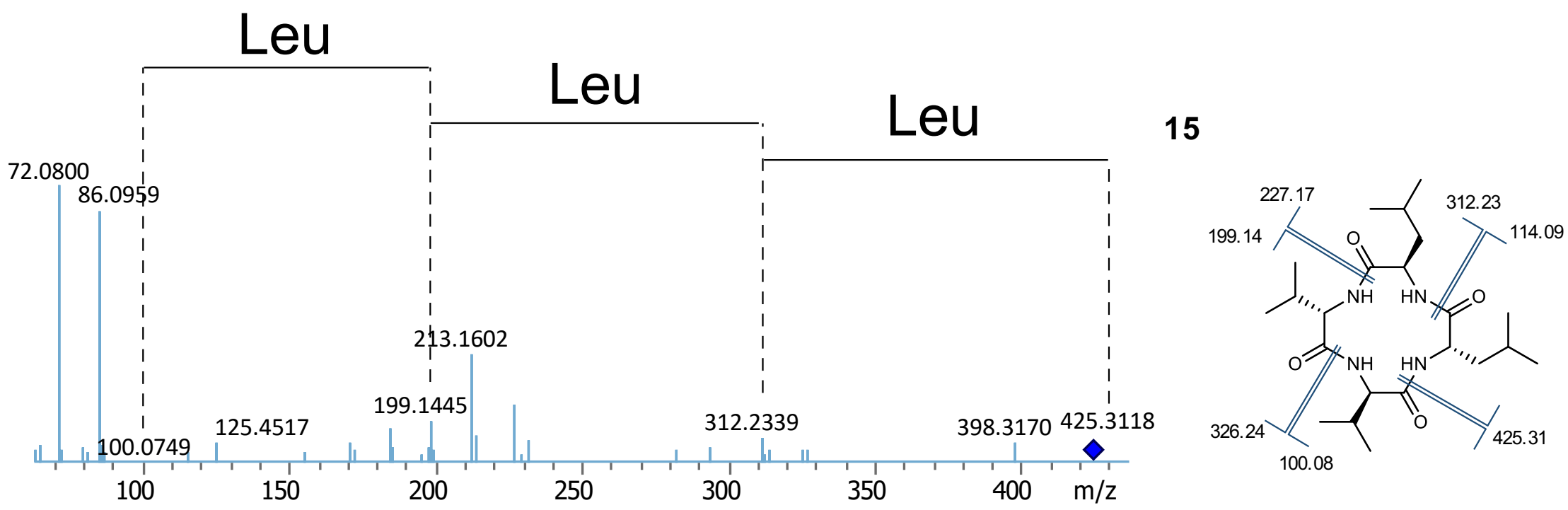

Supplementary Figure 11. a, Base peak chromatograms (BPCs) and extracted ion chromatograms (EICs) of GxhS-1 expressed peptides. Distinct peaks corresponding to peptide products are numbered as **9–15**. **b–h**, MS/MS spectrum and structural annotations of **9–15**.

a

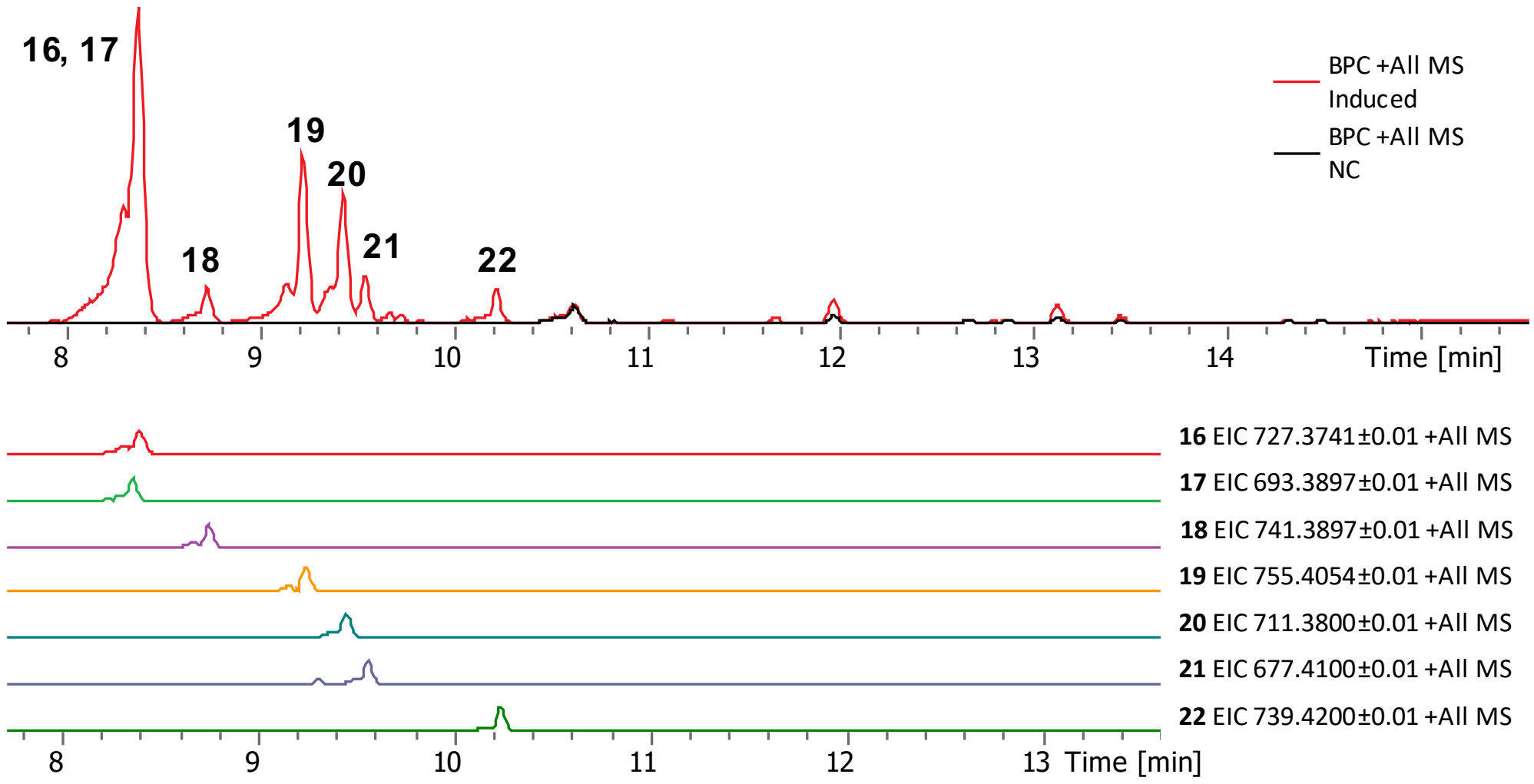

b

vLfYW (m/z 727.3814, [M+H]⁺)

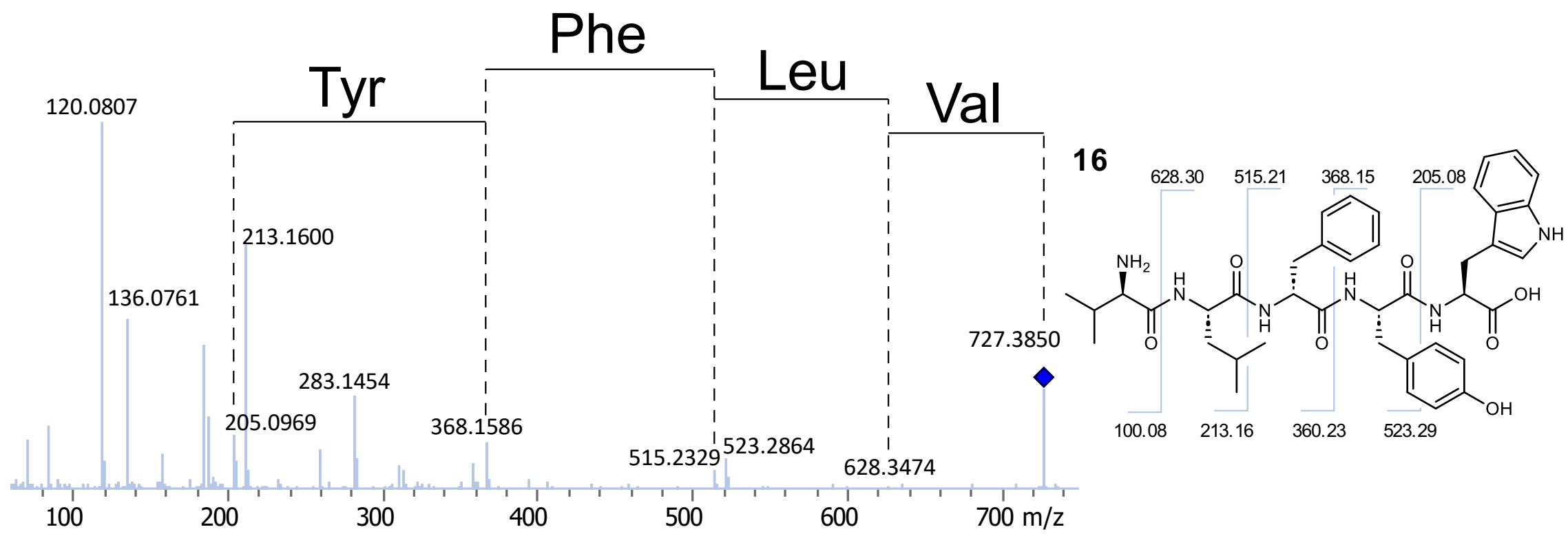

c

vLlYW (m/z 693.3970, [M+H]⁺)

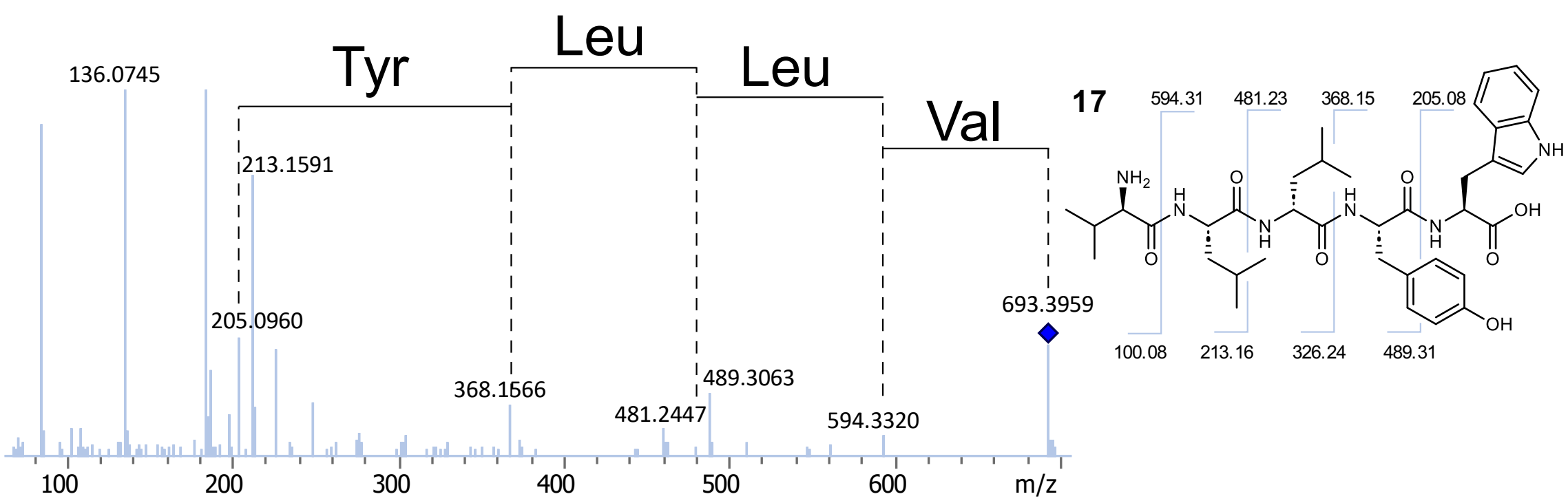

g

vLlFW (m/z 677.4021, [M+H]⁺)

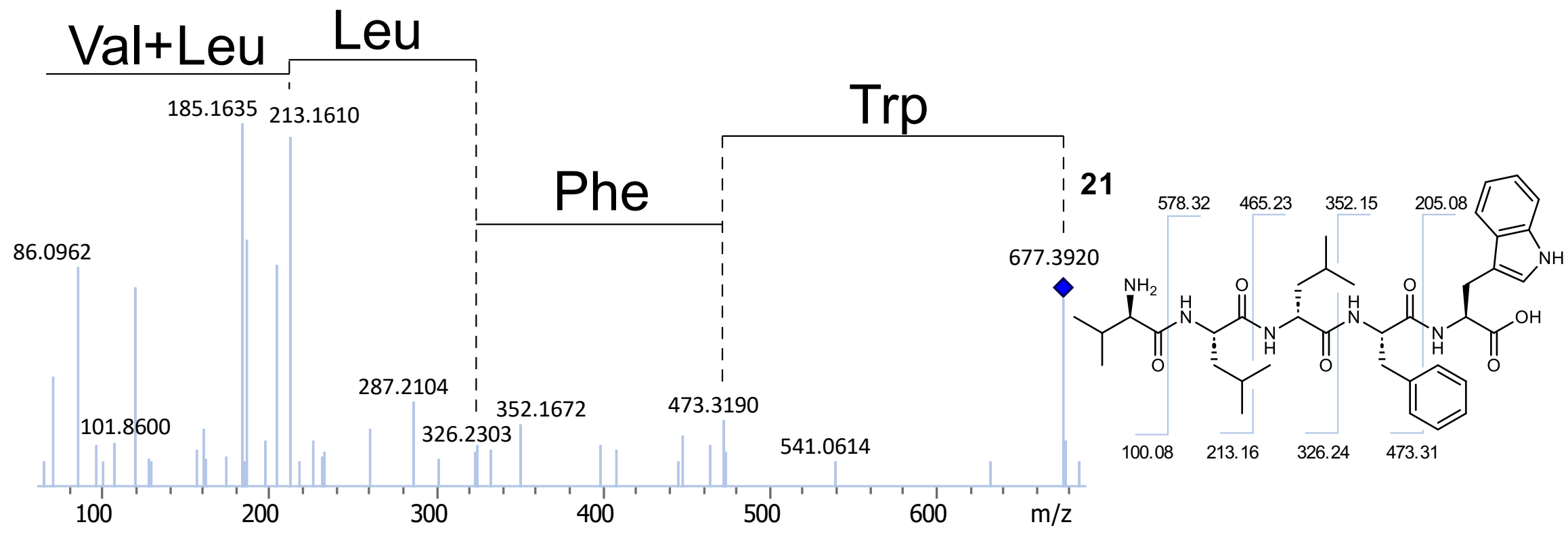

h

vLfFW-OEt (m/z 739.4178, [M+H]⁺)

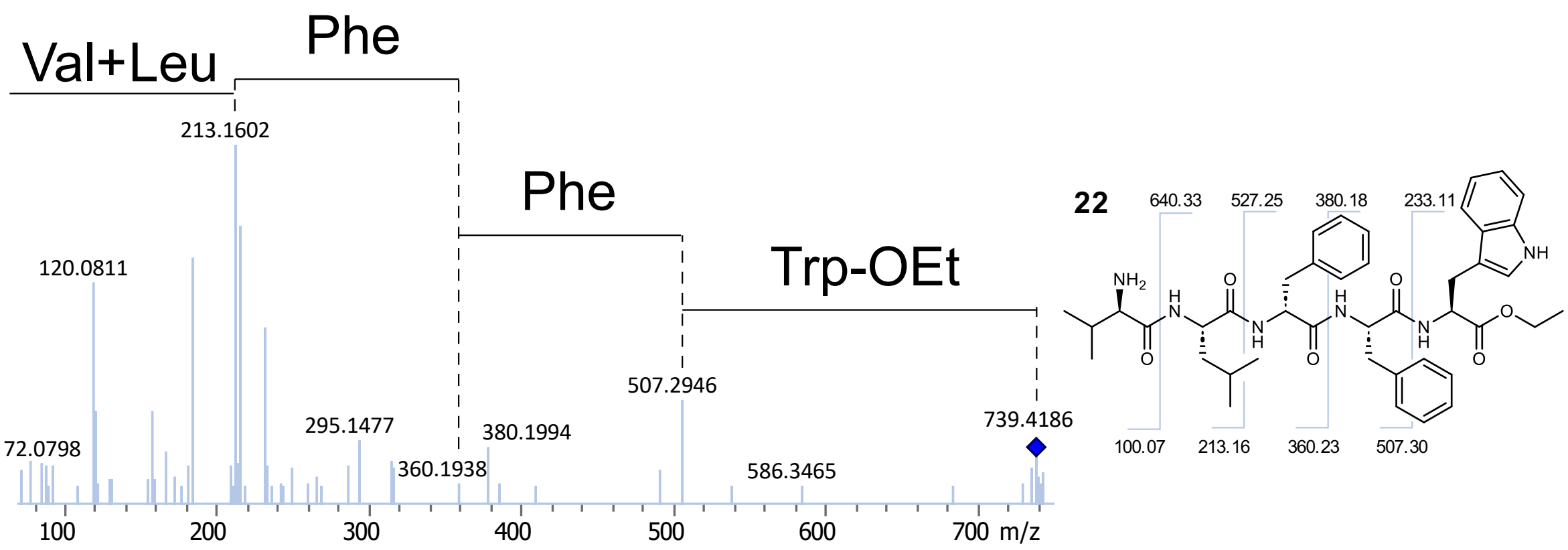

Supplementary Figure 12. **a,** Base peak chromatograms (BPCs) and extracted ion chromatograms (EICs) of GshS expressed peptides. Distinct peaks corresponding to peptide products are numbered as **16–22**. **b–h**, MS/MS spectrum and structural annotations of **16– 22**.

a

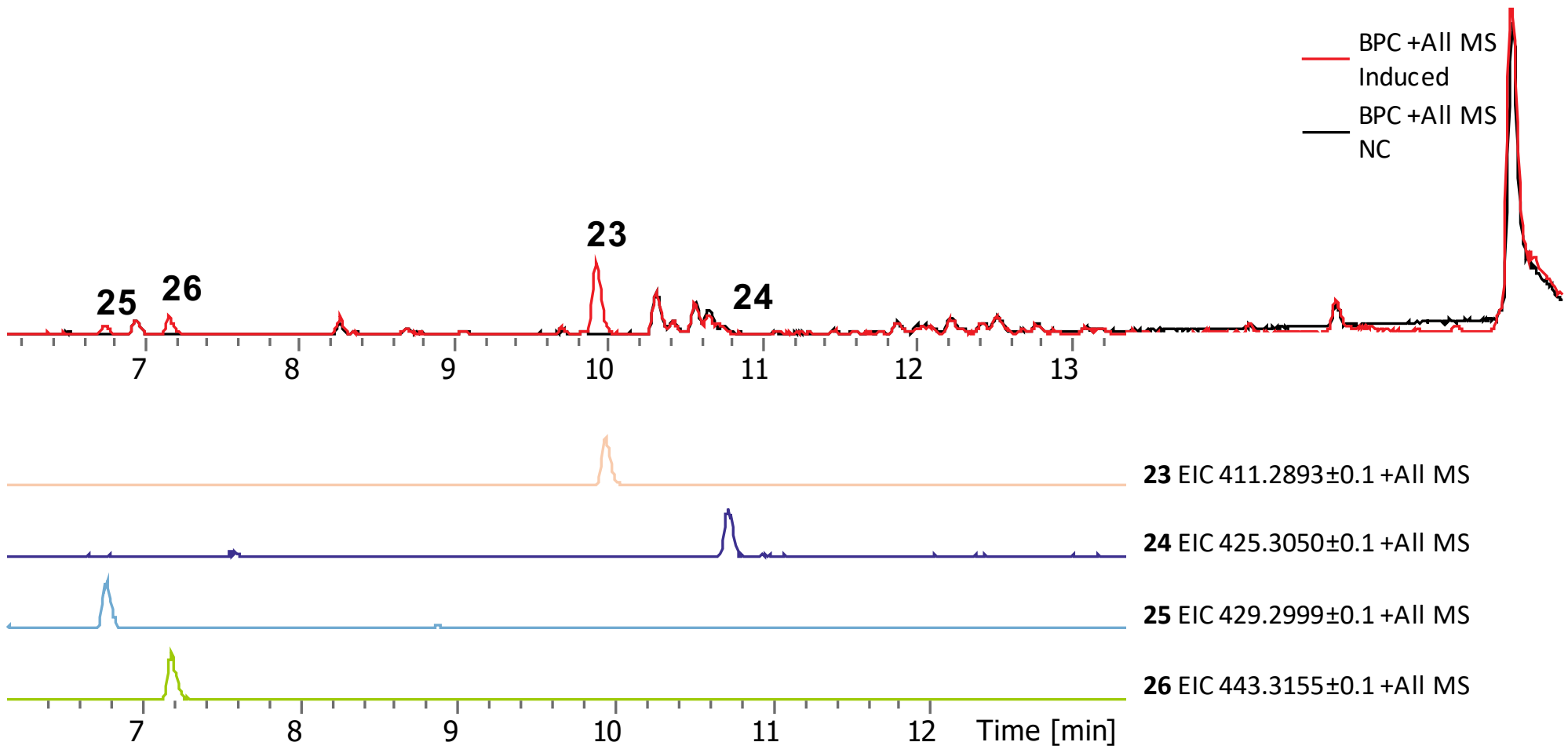

b

cyclo vLvV (m/z 411.2966, $[M+H]^+$)

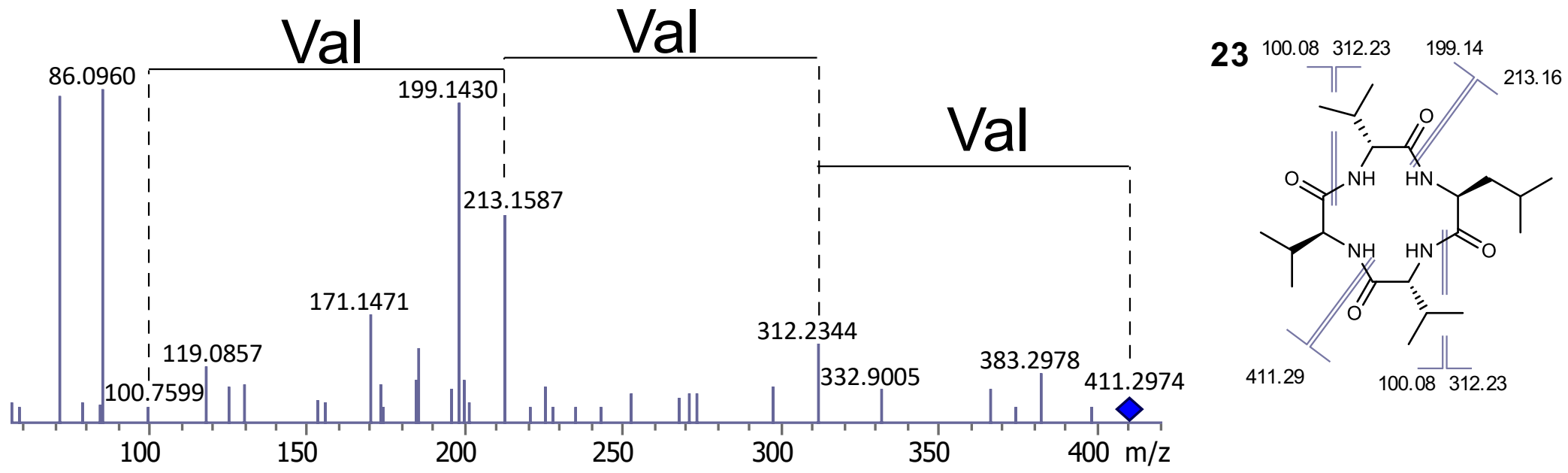

c

cyclo lLvV (m/z 425.3122, $[M+H]^+$)

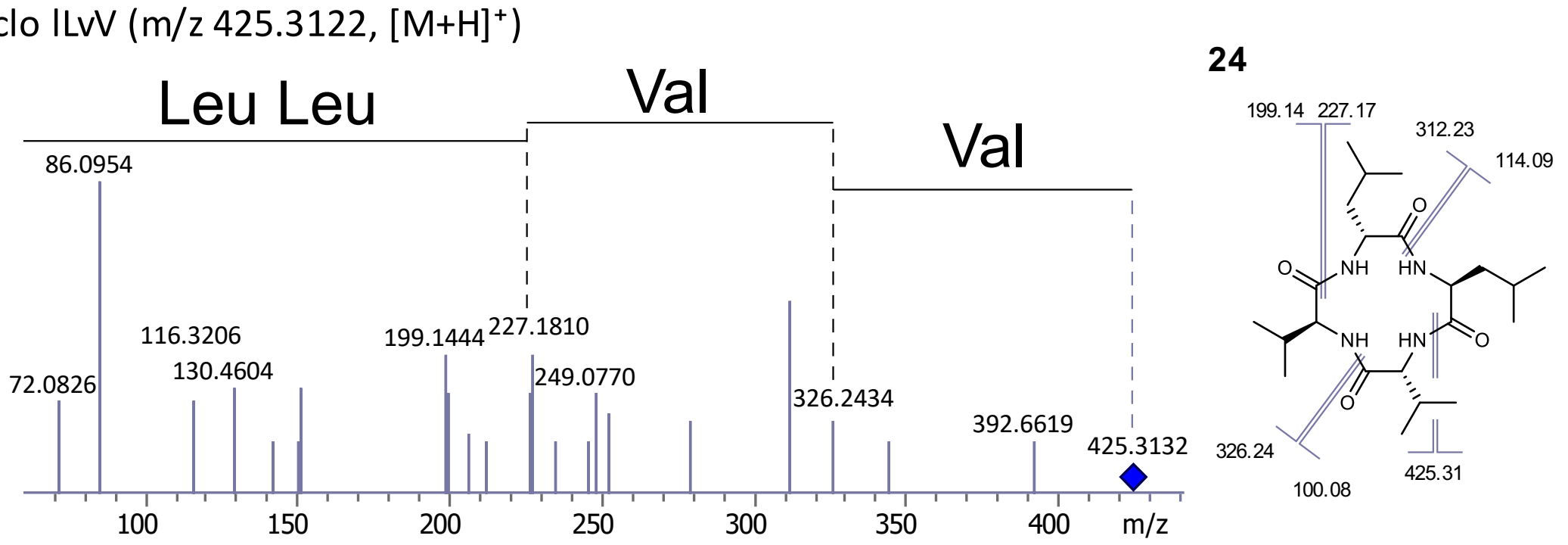

d

vLvV (m/z 429.3071, $[M+H]^+$)

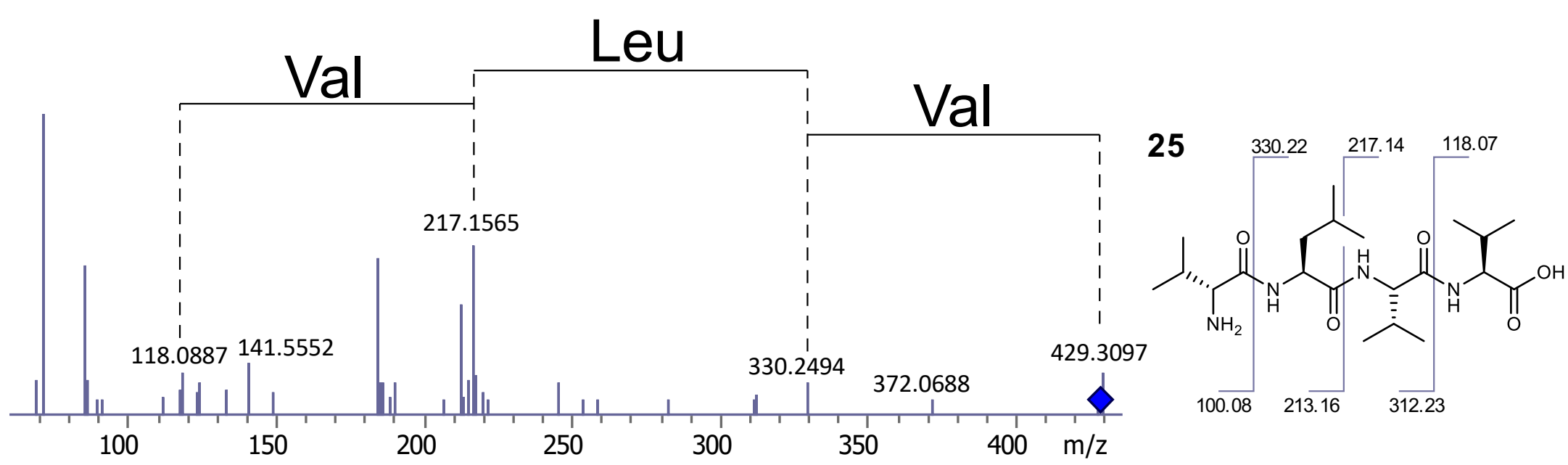

e

lLvV (m/z 443.3228, [M+H]⁺)

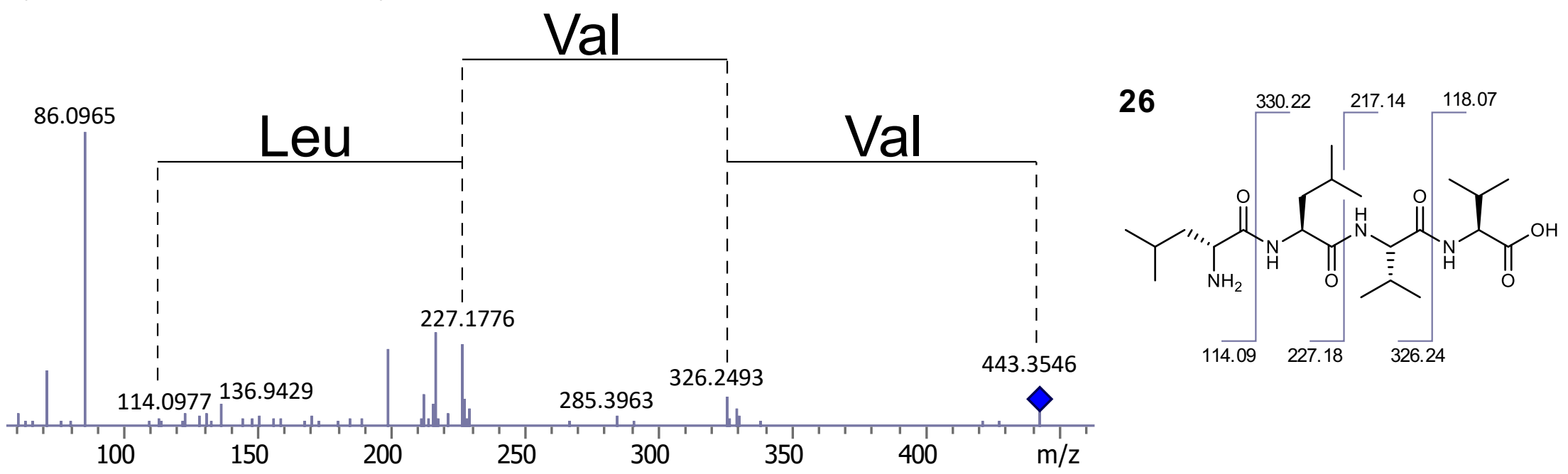

Supplementary Figure 13. **a,** Base peak chromatograms (BPCs) and extracted ion chromatograms (EICs) of GxhS-2 expressed peptides. Distinct peaks corresponding to peptide products are numbered as **23–26. b - e**, MS/MS spectrum and structural annotations of **23–26**.

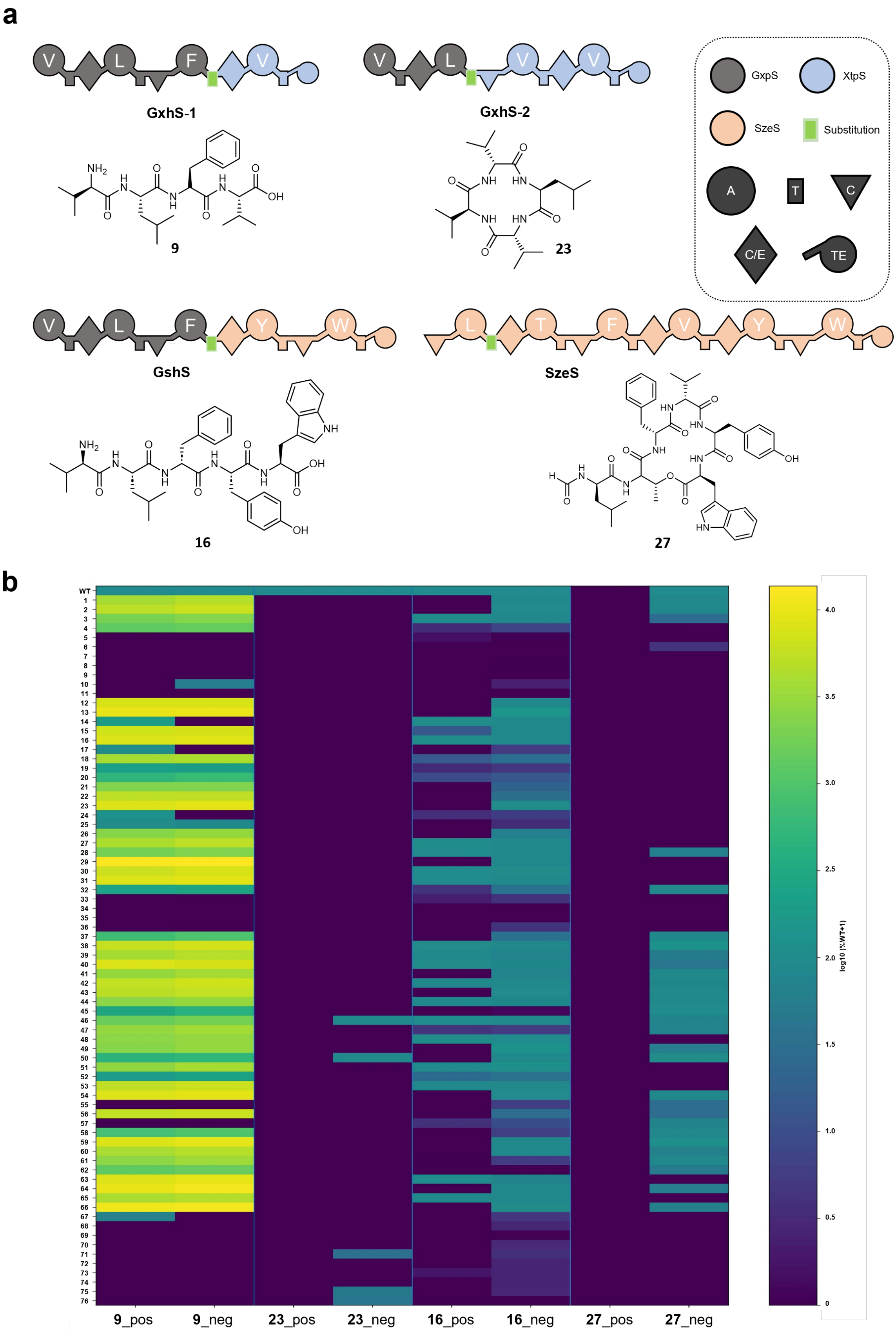

Supplementary Figure 14. RapidFire LC–MS screening of T-domain variants across GxhS-1, GxhS-2, GshS, and SzeS systems. **a,** Schematic representation of the native and hybrid NRPS architecture used for screening (GxhS-1, GxhS-2, GshS, and SzeS) and the corresponding major peptide products detected in each system. **b**, Heatmap shows WT-normalized product formation (%WT) for the main product detected in each system: vLfV (**9**) (GxhS-1), cyclo(vLvV) (**23**) (GxhS-2), vLfYW (16) (GshS), and cyclo(lTfvYW) (**27**) (SzeS), across all T-domain variants. Only the dominant product in each system is shown; additional minor

side products detected by RapidFire LC–MS are summarized in Supplementary Data 1. Positive- and negative-ionization mode peak heights were normalized independently to the corresponding wild-type (WT) signal for each product (WT = 100 %) after background subtraction and setting negative values to zero. For SzeS, the WT signal was detected only in negative ionization mode; therefore, normalization and visualization are shown exclusively for the negative mode, and positive-mode data are omitted. Columns represent system–ionization-mode pairs (e.g., GshS vLfYW_positive_mode and GshS vLfYW_negative_mode), and rows represent T-domain variants labeled by variant number (y-axis). The color scale represents log10(%WT + 1) to compress dynamic range while preserving quantitative relationships (100 % WT corresponds to log10(101) ≈ 2.0). Values of 0 indicate no detectable product above the background. Source data are provided as a Source Data file.

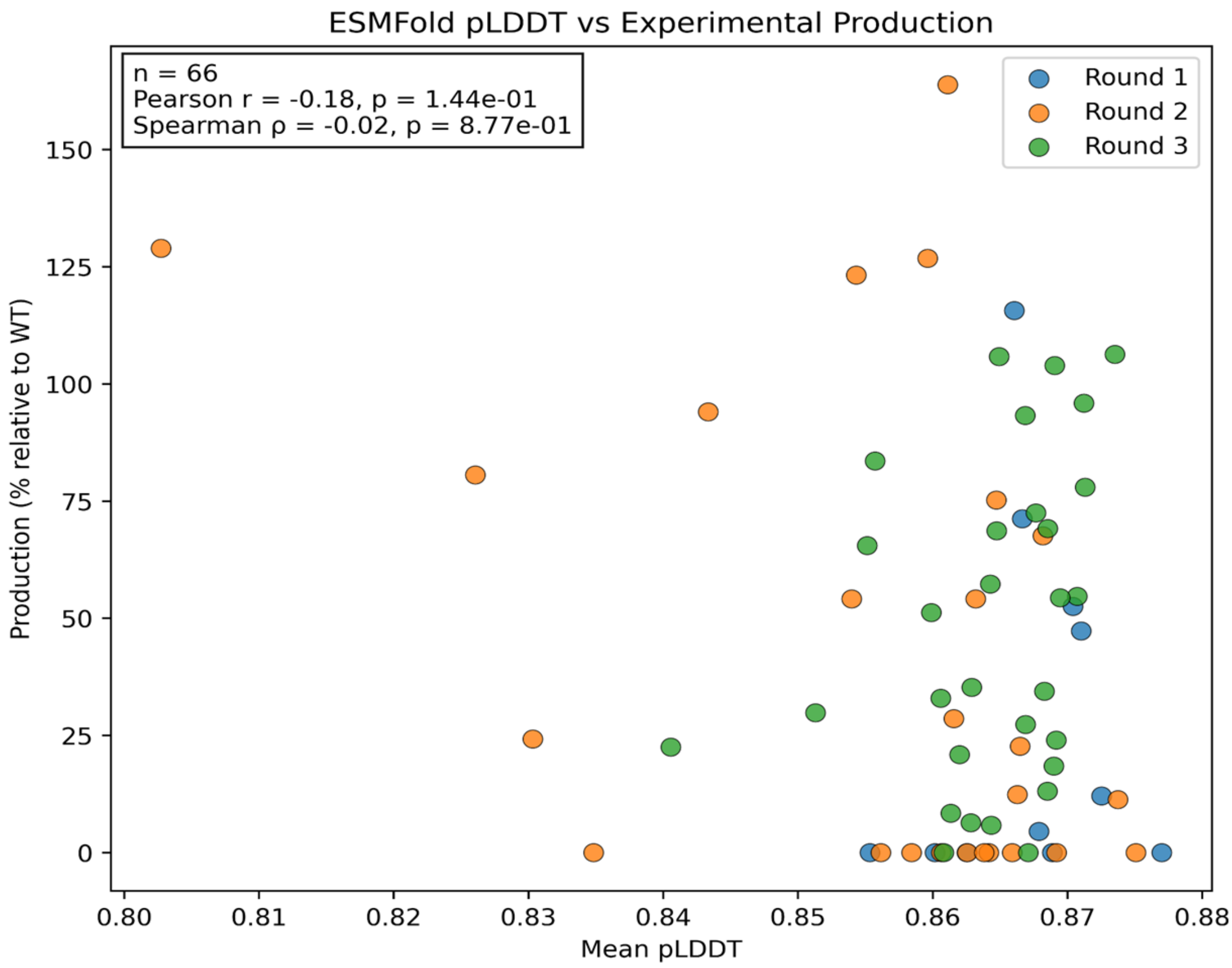

Supplementary Figure 15. Relationship between ESMFold prediction confidence and experimental production. Scatter plot of mean pLDDT scores predicted by ESMFold versus experimentally measured production levels (% relative to WT) for 66 AI-designed T-domain variants. Points are colored according to the three design rounds. No significant correlation was observed between mean pLDDT and production (Pearson $r = -0.18$, $p = 0.144$; Spearman $\rho = -0.02$, $p = 0.877$), indicating that pLDDT is not predictive of production performance in this dataset and should be interpreted as a measure of structure prediction confidence rather than functional activity. Source data are provided as a Source Data file.

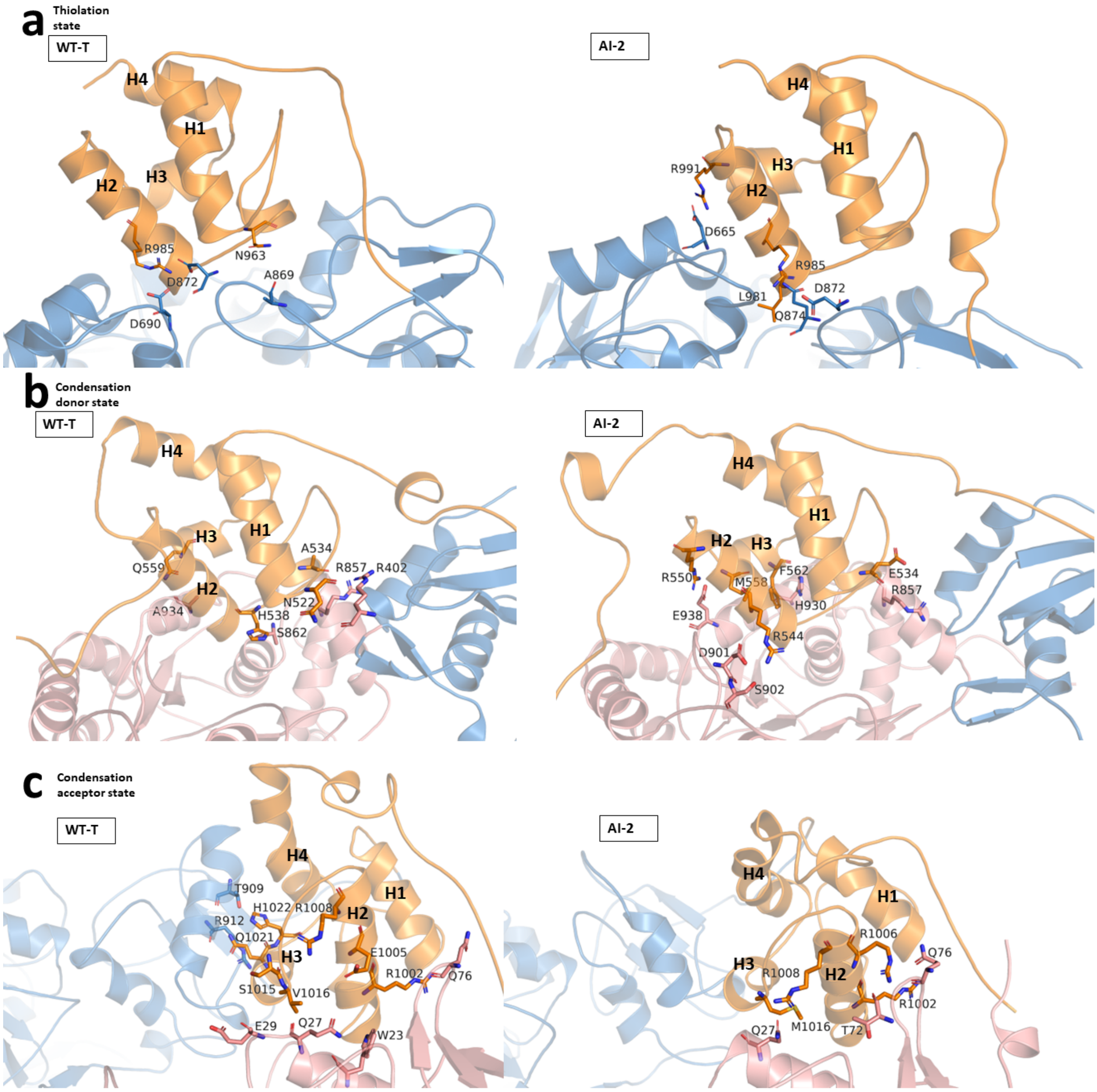

Supplementary Figure 16. State-dependent interdomain interactions of WT GxpS_T3 and AI-2 T-domains across the NRPS catalytic cycle. Detailed views of representative persistent hydrogen-bond interactions identified from molecular dynamics simulations are shown for the thiolation state (a), condensation donor state (b), and condensation acceptor state (c). The T-domain is shown in orange, the adenylation (A) domain in blue, and the condensation (C) domain in salmon. Residues participating in persistent interdomain interactions are displayed as sticks, with T-domain residues shown in orange, A-domain residues in blue, and C-domain residues in salmon. Major structural elements of the T-domain are labeled H1–H4. Interactions were selected based on hydrogen-bond occupancies of ≥30% during the MD simulations. Complete hydrogen-bond occupancy data and interaction statistics are provided in Supplementary Data 1.

Supplementary Note 1: AI workflow for generation of T-domains

Library generation using generative protein models

T-domain sequences were generated using three pretrained generative models: ESM3[5], EvoDiff[6] and ProteinMPNN[7]. For all models, we followed a conditional sequence design strategy within the native GxpS A3–T3–C/E4 tridomain context. The amino acid sequences of the flanking GxpS A3 and C/E4 domains were kept fixed and used as conditioning context, while the T3-domain sequence was masked and regenerated. This ensured that each method produced T-domain variants explicitly conditioned on their intended architectural neighborhood.

ProteinMPNN

We used the openly available 0.02 Å ProteinMPNN model (*v_48_002,* https://github.com/dauparas/ProteinMPNN/tree/main/vanilla_model_weights) for T-domain design, chosen for its minimal structural perturbation. Empirically, this version performed best among the ProteinMPNN variants for our application. We set the sampling temperature to 0.3 to balance sequence diversity and sequence recovery. The remaining parameters were kept at their default values. We provided a homology model of the GxpS A3–T3–C/E4 tri-domain, created using homology modelling via SWISS-MODEL[8] based on crystal structures (PDB IDs 6MFY[9], 6MFZ[9], and 6MG0[9]), as input to the model. During decoding, the amino acid residues of the A- and C- domains were fixed, ensuring that only the T-domain was generated.

ESM3

ESM3 is a multi-modal large language model that can reason across sequence, structure, and function modalities. We used the publicly available (https://github.com/evolutionaryscale/esm) 1.4B parameter version for our design tasks. Two types of designs were generated. In the first setting, both sequence and structure tracks were used for T-domain generation. An AlphaFold3-predicted GxpS A3–T3–C/E4 tridomain structure was supplied through the ESM3 structure track together with the corresponding A3–T3–C/E4 sequence, in which the T3-domain region was masked using the mask token. These designs are labelled with _str_ (see Supplementary Data 1). In the second setting, only the sequence track was used. A decoding temperature of 0.5 was used for all generations. Decoding was performed iteratively, and the number of steps was set to half the length of the T-domain. The quality of the input structures was assessed prior to design. Both the SWISS-MODEL A3–T3–C/E4 homology model used for ProteinMPNN and the AlphaFold3 A3–T3–C/E4 model used for the ESM3 structure track reproduced the canonical T-domain (PCP) fold and the expected positioning of the conserved catalytic residues, including the phosphopantetheine-accepting serine. These observations supported their use for structure-conditioned sequence generation.

EvoDiff

We used the publicly available (https://github.com/microsoft/evodiff) 640M parameter order-agnostic autoregressive diffusion (OADM) model. The A-T-C tri-domain sequence was provided as input, with the T-domain region masked. All parameters were kept at their default values.

Candidate selection across design rounds

Round 1 was designed as an exploratory assessment of out-of-the-box model performance. We generated 100 sequences each using ESM3[5] and ProteinMPNN[7]. From this pool of 200 sequences we selected 11 candidate T-domains (AI-1–AI-11; four ESM3- and seven ProteinMPNN-derived) by manual inspection, without activity prediction, perplexity/likelihood filtering, structural ranking, or surrogate-model guidance. Selection was guided by two

considerations: representation of both generative models, and coverage of the diversity of the phosphopantetheinylation core motif (the FFxxGGxS region, and specifically the residue immediately preceding the catalytic serine) present in the generated designs. All ESM3 designs uniformly retained the canonical His–Ser motif (GGHS), whereas the ProteinMPNN designs varied the residue at this position. We therefore selected four ESM3 designs to sample sequence diversity within the conserved-motif background, and one ProteinMPNN design for most of the distinct core-motif variants produced, representing the majority of the core-motif variation among the ProteinMPNN designs: His (GGHS, AI-7) and the non-canonical residues Ala (in the GGHAS context, AI-5), Gly (AI-6), Glu (AI-8), Asn (AI-9), Asp (AI-10) and Thr (AI-11). This selection, spanning the observed phosphopantetheinylation core-motif diversity, was intended to probe motif tolerance in an out-of-the-box setting. Only designs retaining a naturally occurring residue at this position (His in AI-7 and Asp in AI-10) supported production. This functional split directly motivated the FFxxGGxS motif filter introduced in Round 2. To visualize the diversity of the generated sequence pool, all 200 sequences were embedded using the ESM2 (650M) protein language model. The resulting embeddings were partitioned by k-means clustering, with the number of clusters estimated using the elbow method, and visualized by PCA and UMAP projection (Supplementary Fig. 17). The selected variants are distributed across the resulting embedding space while representing both generative models. Consistent with the known correlation between the residue preceding the phosphopantetheinylation serine and downstream C-domain identity[10], this position distinguished functional from non-functional Round 1 ProteinMPNN designs: in the C/E-type context of GxpS_T3, functional designs retained a naturally occurring residue (His, or Asp in AI-10), whereas non-functional designs introduced residues absent from natural T-domains at this position (AI-6, AI-8, AI-11), shifted the motif register (AI-5), or used a natural but non-C/E-associated residue (Asn; AI-9).

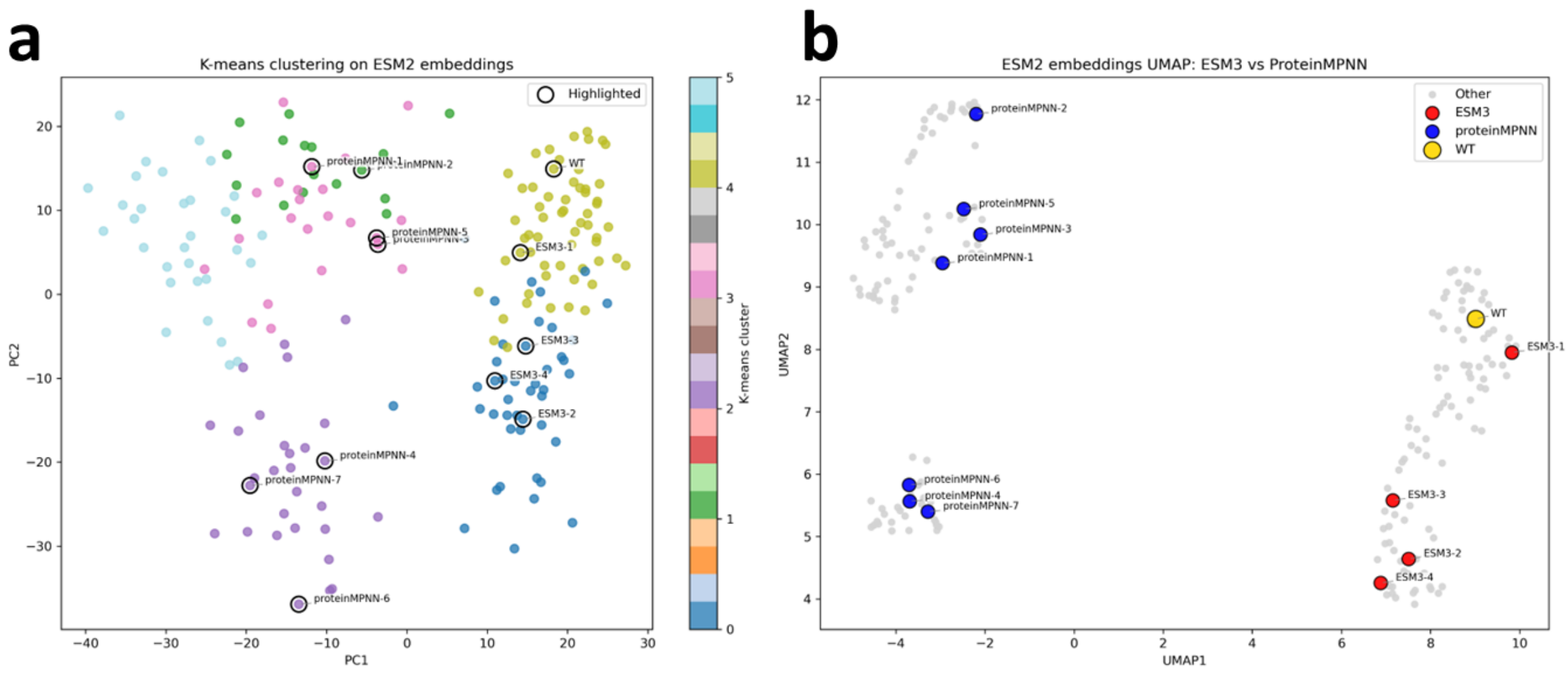

Supplementary Figure 17. ESM2 embedding of the Round 1 generated pool, illustrating the diversity of the selected AI-designed T-domain variants. **a,** Principal component analysis (PCA) of ESM2 sequence embeddings for the 200 generated T-domain variants (100 generated by ESM3 and 100 by ProteinMPNN). Sequences are colored by k-means cluster, with the number of clusters determined from the data using the elbow method; the clustering is shown only to illustrate the diversity of the generated pool. The 11 experimentally tested variants are highlighted (AI-1 to AI-4, ESM3-derived; AI-5 to AI-11, ProteinMPNN-derived) and were selected to span both generative models and the full range of core-motif variants, not as one representative per cluster. **b,** Uniform Manifold Approximation and Projection (UMAP) of the same ESM2 embeddings. The selected ESM3-derived variants (AI-1 to AI-4, red), ProteinMPNN-derived variants (AI-5 to AI-11, blue), and the wild-type T domain (yellow) are highlighted, illustrating the distribution of the selected candidates across the learned sequence embedding space while representing both generative models. Source data are provided as a Source Data file.

Round 2 introduced sequence-based filtering. We generated 400 sequences per model using the following three models: ESM3 with structural conditioning, ESM3 without structural conditioning, and EvoDiff[6]. Sequences were required to retain the conserved FFxxGGxS motif,

exhibit >50% sequence identity to WT GxpS_T3, and have an ESM2 perplexity score below 4.8. We computed ESM2 embeddings for the remaining sequences and, for each generation method separately (ESM3 with structural tokens, ESM3 without structural tokens, and EvoDiff), clustered the sequences into five groups. The sequence with the lowest perplexity from each cluster was selected as a representative candidate for experimental testing. In addition, five ProteinMPNN-derived sequences from Round 1 were revisited after restoration of the conserved motif, and five likelihood-guided optimized sequences were included, resulting in 25 experimentally tested variants.

Round 3 employed surrogate-model-guided prioritization using the experimental data generated in Rounds 1 and 2 together with the expanded error-prone mutagenesis dataset, for details, see the section Surrogate Models. To generate the Round 3 candidate library, ESM3 and EvoDiff were used to produce 5,000 T-domain sequences conditioned on the A- and C-domain context. Subsequent filtering and prioritization procedures are described below.

Generation of $^{L}C_{L}$-context T-domains (AI-67–AI-76). To evaluate context-conditioned design for an $^{L}C_{L}$ -type downstream interface, T-domains were generated with ESM3 and EvoDiff, conditioned on an A–T–C context comprising the GxpS A2 domain and the XtpS C3 domain (an $^{L}C_{L}$ -type condensation domain), with the T-domain masked and regenerated. Candidate selection followed the same procedure as in Round 2: sequences were filtered for retention of the conserved FFxxGGxS motif, a minimum sequence-identity threshold, and ESM2 perplexity, after which ESM2 embeddings were clustered into five groups per generation method and the lowest-perplexity representative of each cluster was selected, yielding 10 variants (AI-67–AI-76; five per model).

In silico evaluation

To evaluate the generated T-domains, we assessed the sequences using multiple *in silico* metrics. Detailed descriptions are provided below.

Sequence Identity: This metric provides a measure of similarity between the generated sequences and the wildtype T-domain. To calculate sequence identity, we first align the two sequences using *hmmalign* from HMMER[11]. The HMM profile for the T-domain was retrieved from InterPro (PFAM Entry PF00550)[12]. Using sequence identity as an evaluation criterion, we assess the ability of the models to generate plausible T-domain sequences for the given context. Higher sequence identity values suggest that a model produces sequences that are more structurally and functionally aligned with the wildtype.

Structural Confidence and Structural Similarity: To assess structural plausibility, we refold the generated sequences and evaluate their predicted structures. We use ESMFold[13] to predict the structures of the designed T-domains.

- For structural confidence assessment, we used the pLDDT scores reported by ESMFold. For each generated sequence, the mean pLDDT was calculated as an indicator of structural prediction confidence and compatibility with the expected T-domain fold.
- For structural similarity, we compare the predicted structure to the ground-truth structure. We use TM-align to compute the TM-score[14,15]. Higher TM-scores indicate that the generated sequences fold into structures closely resembling the original, potentially retaining similar functionality.

Perplexity: Perplexity is commonly used in natural language processing (NLP) to evaluate how well a probabilistic model predicts a sequence[16]. In our design setting, we use perplexity

to assess the biological plausibility of the T-domain sequences generated by our models. We use the 650M ESM2 model[13] to calculate perplexity. We estimate perplexity as follows (Equation 1):

$$perplexity = exp\left(-\frac{1}{L}\sum_{i=1}^{L} log\, p(x_i|x_{-i})\right) \qquad (1)$$

where $L$is the length of the amino acid sequence and $p(x_i|x_{-i})$is retrieved from the softmax probabilities of ESM2 at position i, when position $i$is masked.

Surrogate Models

For round 3, we implemented a surrogate model to guide the selection of candidate T-domains for experimental testing. We used the T-domain sequences and their corresponding activity values from rounds 1 and 2 as the training set. In total, this dataset comprised 85 labeled sequences evaluated in the SU2-only GxpS assay, using fIL (**1**) and FIL (**2**) production as the quantitative fitness readout. We evaluated multiple model architectures based on their ability to rank T-domain sequences. Specifically, we considered three categories: (i) models using pretrained protein language model (PLM) embeddings as protein representations, (ii) zero-shot fitness scores derived from PLMs, and (iii) fine-tuned PLMs. We used the ESMC 600M[17] as the base PLM. We also evaluated ESM2, but ESMC consistently provided better performance.

For models using pretrained representation, we adopted two strategies to obtain the final representation: (i) mean pooling of embeddings across the residue dimension, and (ii) unrolling the residue-level embeddings into a single concatenated vector. On top of these representations, we evaluated three prediction heads: a linear regression head, a shallow multi-layer perceptron (MLP), and a random forest regressor. In all cases, only the prediction head was trained, while the PLM parameters remained frozen. Models were trained to predict T-domain activity using mean squared error (MSE) loss.

For mean-pooled embeddings, the random forest and shallow MLP achieved comparable performance and outperformed the linear head. We therefore selected the random forest for subsequent experiments. This was motivated both by its strong empirical performance and by its successful application in the recent EvolvePro[18] framework. In contrast, for the unrolled embedding representation, a linear head performed best and was used in subsequent experiments. We hypothesize that the random forest and MLP were more prone to overfitting in this high-dimensional setting, whereas the simpler linear model generalized better.

Meier et al. (2021) demonstrated that PLM-derived likelihoods can be used for zero-shot fitness prediction and outlined multiple approaches for scoring protein variants based on these likelihoods[19]. In this study, we used the masked marginal score, estimated by computing the log-odds ratio of the mutant amino acid relative to the wildtype amino acid at each mutated position. Let Mdenote the set of mutated positions in a variant relative to the wildtype sequence. The masked marginal fitness score is defined in (Equation 2):

$$\widehat{f_\theta}(x) = \sum_{i\in M} log\, p(x_i^{mt}|x_{-M}) - log\, p(x_i^{wt}|x_{-M}) \qquad (2)$$

where x_{-M}denotes the sequence with masked tokens at the mutated positions, and $x_i^{mt}$and x_i^{mt}represent the amino acid identities at position $i$in the mutant and wildtype sequences, respectively.

For fine-tuning ESMC, we adopted two strategies. The first followed a conventional setup, in which we extracted the class token (<CLS>) embedding and applied a linear layer to predict activity values[20]. To enable efficient fine-tuning and reduce overfitting, we use Low-Rank Adaptation (LoRA) to update only a fraction of the total model parameters[21]. This model was

trained using MSE loss. The second approach employed a contrastive fine-tuning strategy using a ranking objective[22]. The loss function was based on the Bradley–Terry (BT) model, which enforces pairwise ranking consistency. Given two sequences, $x^{(i)}$ and $x^{(j)}$, with corresponding fitness values $y^{(i)}$ and $y^{(j)}$, where $y^{(i)} > y^{(j)}$, the BT loss is defined in (Equation 3):

$$L_{BT} = \sum_{y^{(i)}>y^{(j)}} log \left[1 + e^{-\left(\widehat{f_\theta}(x^{(i)}) - \widehat{f_\theta}(x^{(j)})\right)}\right] \qquad (3)$$

where $\widehat{f_\theta}$ represents the fitness function parameterized by θ. We used the masked marginal score described above as the fitness function. To prevent overfitting, we introduced a Kullback–Leibler (KL) divergence regularization term (Equation 4):

$$L_{KL} = D_{KL}\left(P_\theta || P_{\theta_0}\right) = \sum_i P_\theta(x_i|x_{-i}) \, log \frac{P_\theta(x_i|x_{-i})}{P_{\theta_0}(x_i|x_{-i})} \qquad (4)$$

where P_θ represents the probability distribution of the fine-tuned PLM and P_{θ_0} corresponds to the distribution of the pretrained PLM. The final training objective is formulated in (Equation 5):

$$L = L_{BT} + \lambda L_{KL} \qquad (5)$$

where λ is a hyperparameter controlling the contribution of the KL divergence regularization term. LoRA was also used in this setting to fine-tune ESMC.

Training details

For models using pretrained embeddings, we first extracted sequence embeddings from the final layer of ESMC for all sequences and stored them. These embeddings were then used as input to downstream predictive models. We implemented the linear model using the Ridge module from scikit-learn (version 1.4.2) with default parameters and random forest using RandomForestRegressor module.

For fine-tuning ESMC in both settings described above, we employed LoRA with rank 8, alpha 8, and a dropout rate of 0.1. LoRA adapters were applied to the key, query, and value projection layers, as well as to the feed-forward network following the Multi-Head Attention block. For contrastive fine-tuning, LoRA was additionally applied to the sequence head and the KL divergence regularization weight (λ) was set to 0.1. Training was performed with early stopping using a patience of 10 epochs. A batch size of 8 was used with gradient accumulation over 4 steps, resulting in an effective batch size of 32. Experiments were conducted using PyTorch version 2.3.1 and PyTorch Lightning version 2.5.

Results

The surrogate models were benchmarked using 85 T-domain sequences from rounds 1 and 2. These were split into training (n = 55) and test (n = 30) sets. To minimize data leakage and ensure a rigorous evaluation, we performed the split based on Hamming distance, such that sequences in the test set were maximally distant from those in the training set. In the final split, the nearest test sequence was at least 25 mutations away from any training sequence. Models were trained on the training set, and performance was evaluated on the test set using Spearman rank correlation between predicted and experimentally measured activity (Supplementary Table 5). We see that the unrolled pretrained ESMC embedding with a linear head achieved the highest mean performance, followed by contrastive fine-tuning.

Supplementary Table 5. Mean values and 95% confidence intervals (CI) for Spearman correlation on the T-domain test set. CI was estimated using bootstrapping. RF: Random Forest. The code used to generate these benchmarks is available as described in the Code Availability statement.

Model	T-domain
Mean pooling + RF head	0.25 (-0.10, 0.59)
Unrolled + linear head	**0.57** (0.26, 0.83)
Masked marginal score	0.35 (-0.05, 0.74)
MSE-based finetuning	0.38 (0.00, 0.72)
Contrastive finetuning	0.43 (0.11, 0.73)

To have a broader comparison, we further evaluated our models on two widely used protein fitness prediction datasets. First, we used the GB1 dataset, comprising variants of the first binding domain of protein G with one to four mutations at defined positions[23]. We adopted the three-vs-rest split, in which variants containing up to three mutations were used for training and four-mutation variants were reserved for testing[20,24]. Second, we evaluated the TEM1 β-lactamase dataset, which captures the fitness effects of single amino acid substitutions across the protein[25]. Here, we applied a contiguous split scheme[26], dividing the protein sequence into ten segments, with two segments designated as test data and the remaining eight used for training. Results are reported in Supplementary Table 6. On GB1, MSE-based fine-tuning achieved the best performance, whereas contrastive fine-tuning performed best on TEM1. Notably, the masked marginal score performed comparatively well on TEM1 but poorly on GB1. This shows that zero-shot likelihood-based scores may be dataset dependent and that task-specific adaptation, either via fine-tuning or training a predictive head, is necessary for optimal performance.

Supplementary Table 6. Mean values and 95% confidence intervals (CI) for spearman correlation on both the GB1 and TEM1 dataset. CI was estimated using bootstrapping. RF: Random Forest The code used to generate these benchmarks is available as described in the Code Availability statement.

Model	GB1	TEM1
Mean pooling + RF head	0.5 (0.48, 0.52)	0.74 (0.71, 0.76)
Unrolled + linear head	0.74 (0.73, 0.76)	0.84 (0.82, 0.85)
Masked marginal score	0.29 (0.26, 0.31)	0.82 (0.81, 0.84)
MSE-based finetuning	**0.81** (0.80, 0.82)	0.71 (0.69, 0.74)
Contrastive finetuning	0.77 (0.76, 0.78)	**0.88** (0.87, 0.89)

To better mimic the low-data regime of the T-domain setting, we simulated low-data training scenarios on both GB1 and TEM1 while retaining their full test sets for robust evaluation. Specifically, we constructed training subsets of size 50, 100, and 200, sampled such that their fitness distributions closely matched that of the T-domain training set. This analysis enabled us to assess model robustness under limited data and examine scaling behavior with

increasing training size (see Supplementary Table 7). In these low-data regimes, MSE-based fine-tuning performed poorly in most settings. In contrast, except for GB1 at N = 200, contrastive fine-tuning consistently outperformed alternative approaches across datasets.

Supplementary Table 7. Performance on both the GB1 and TEM1 dataset under low-data setting. Each model was trained five times with different random initializations and the reported values correspond to the mean and standard deviation across these five runs. RF: Random Forest. The code used to generate these benchmarks is available as described in the Code Availability statement.

Dataset	Models	Low-data Setting		
		N = 50	N = 100	N = 200
GB1	Mean pooling + RF head	0.49±0.04	0.51±0.05	0.56±0.02
	Unrolled + linear head	0.61±0.05	0.7±0.03	**0.75±0.01**
	Masked marginal score	0.29±0.00	0.29±0.00	0.29±0.00
	MSE-based finetuning	0.12±0.03	0.12±0.04	0.15±0.02
	Contrastive finetuning	**0.63±0.04**	**0.71±0.03**	0.74±0.03
TEM1	Mean pooling + RF head	0.58±0.04	0.6±0.01	0.68±0.02
	Unrolled + linear head	0.64±0.01	0.68±0.01	0.73±0.03
	Masked marginal score	**0.82±0.00**	**0.82±0.00**	**0.82±0.00**
	MSE-based finetuning	-0.13±0.05	-0.09±0.05	-0.12±0.02
	Contrastive finetuning	0.78±0.01	0.79±0.01	0.81±0.01

Based on these combined results, we selected contrastive fine-tuning as the primary surrogate model for subsequent rounds. Given the strong performance of the unrolled pretrained embeddings with a linear head, we also retained this approach to provide complementary predictions and increase the robustness of T-domain selection.

Generation and filtering of Round 3 candidates

For round 3, we used ESMC with unrolled embeddings and ESMC with contrastive fine-tuning as our surrogate models. For each model, we trained three ensemble members by bootstrapping the training data. To generate a candidate library for round 3, we used ESM3 and EvoDiff to produce 5,000 T-domain sequences conditioned on the given A- and C-domain context.

First, the generated sequences were first filtered using the same *in silico* metrics applied in round 2. We then removed sequences exhibiting high variance across ensemble predictions. In addition, sequences with a masked marginal score lower than −10 were excluded. Although the masked marginal score did not strongly correlate with activity in rounds 1 and 2, we observed that it was effective in filtering out low-performing sequences.

The remaining sequences were divided into five bins based on the fitness scores predicted by each surrogate model. We then identified sequences that were assigned to the same bin by both models. This voting-like mechanism allowed us to prioritize sequences that were consistently placed in similar fitness categories by both surrogates, thereby increasing confidence in their predicted performance. From each bin, we selected sequences with the

highest contrastive fine-tuning scores for experimental evaluation. The final set of selected sequences is presented in Supplementary Data 1.

Supplementary Methods

Supplementary Method 1. Peptide synthesis and purification

General Remarks

Fmoc-L-Leu-OH, Fmoc-D-Leu-OH, Fmoc-D-Phe-OH, Fmoc-L-Leu-OH, Fmoc-D-Val-OH and O-(7-azabenzotriazol-1-yl)-N,N,N',N'-tetramethyluronium hexafluorophosphate (HATU) were purchased from BLD Pharma. 2-chlorotrityl chloride resin (200–400 mesh, 1% DVB, 0.4–3 mmol g^{-1} capacity), O-(1H-6-Chlorobenzotriazole-1-yl)-1,1,3,3-tetramethyluronium hexafluorophosphate (HCTU), hexafluoroisopropanol (HFIP) and diisopropylethylamine (DIEA) were purchased from abcr GmbH. Dichloromethane (DCM) p.a. and dimethylformamide (DMF) p.a. from VWR International, and anhydrous solvents (DMF, DCM) from Sigma Aldrich. NMR analysis ^{1}H, COSY, HSQC and HMBC spectra were recorded on a Bruker Avance Neo 500 MHz (^{1}H, 500 MHz; ^{13}C, 126 MHz) with prodigy cryoprobe system. Chemical shifts were recorded as δ values in ppm units and referenced against the residual solvent peak (DMSO-d6, δ= 2.50, 39.52 and $CDCl_3$: δ=7.26, 77.16). High-resolution mass spectra were recorded on a ThermoFisher Scientific (TF, Dreieich, Germany) Q Exactive Focus system equipped with heated electrospray ionization (HESI)-II source. Syntheses were carried out manually in PP syringes equipped with PE frits and PTFE stopcocks (Roth Selection).

Peptide synthesis

General procedure 1: Determination of amino acids loading on the resin

Fmoc-L-Leu-OH was loaded on 2CT resin and was determined by spectrophotometric quantification at λ = 301 nm of the dibenzofulvene liberated after deprotection of the Fmoc group with piperidine. To a precisely weighed amount of resin (5 mg) 1 mL of a solution of piperidine/DMF (2:8) was added. After 20 min under shaking, 100 µL were transferred in a quartz cuvette and the volume made up to 1 mL with DMF. The UV absorbance was measured at λ = 280 nm against a control of a solution of 100 µL piperidine/DMF (2:8) diluted with DMF to a volume of 1 mL. Loading was calculated by $L = 1000*D*(A_s-A_b)*V/(\varepsilon_{290}*m)*l$, where D is the dilution factor, A_s the absorbance of the sample at 290 nm and A_b the absorbance of the blank at 280 nm, V is the volume, ε_{290} is a molar extinction coefficient of dibenzofulvene at 290 nm (L mol^{-1} cm^{-1}) and l is the path length of the cuvette.

General procedure 2: Fmoc deprotection

The resin was agitated for 20 min with a solution of piperidine/DMF (2:8), after which the supernatant was removed. The resin was washed subsequently with DMF (3x), methanol (MeOH) (1x) and DCM (3x).

General Procedure 3: Coupling of amino acids to resin-bound peptide

To a solution of Fmoc protected amino acids (3 equiv.) and DIEA (6 equiv.) in DMF (0.2M), HCTU (4 equiv.) was added. The mixture was agitated for 2 min and subsequently added to the pre-swollen resin in DMF. The resin was agitated at rt for 1h, after which the supernatant was removed (completion of coupling checked by treating a small sample of the resin with HFIP to obtain a sample peptide for HPLC/MS analysis) and the resin was washed subsequently by DMF (3x), MeOH (1x) and DCM (3x).

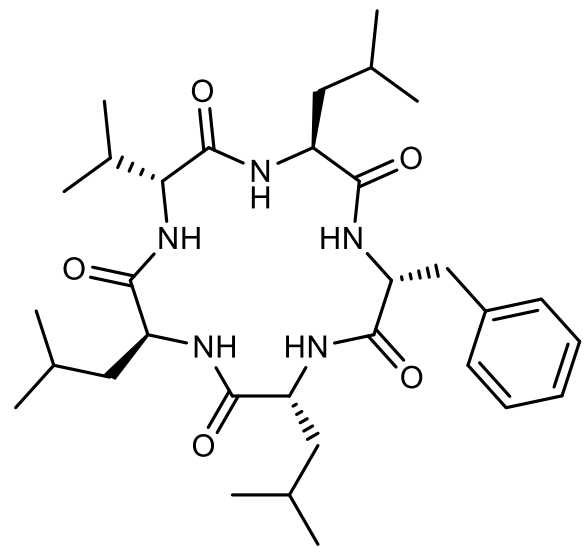

(3R,6S,9R,12S,15R)-3-Benzyl-6,12,15-triisobutyl-9-isopropyl-1,4,7,10,13-pentaazacyclopentadecane-2,5,8,11,14-pentaone (6)

HRMS (ESI) m/z: calculated for $[M+H]^+$: 586.3963; found: 586.3925.

For the synthesis of **6**, Fmoc–SPPS procedure was adapted from the literature.[1] To 1 gr of 2CT resin, 10 mL of DCM were added and agitated for 30 min. The solvent was drained off, and a suspension of Fmoc-L-Leu-OH (1.6 gr, 3 equiv.) and DIEA (1.5 mL, 6 equiv.) in DCM (10 mL) was added to the resin. The resin was shaken overnight, drained off and washed 3xDCM and dried. The loading was calculated as General Procedure 1 (0.75 mmol g^{-1}). The sequent deprotection and coupling steps were carried out following General procedure 2 and General Procedure 3.

After completing the amino acids sequence, the resin was suspended in 1:1 DCM/HFIP (20 mL) solution and agitated for 1 h. This step was repeated twice. The solution was drained off in a flask and evaporated under reduced pressure. The crude was triturated in cold MTB to obtain peptide NH_2-D-Val-Leu-D-Phe-D-Leu-Leu-OH in sufficient purity to perform the macrocyclization step.

The macrocyclization was adapted from reported procedure[2]. The linear peptide was dissolved in DCM (3 mM), followed by the addition of DIEA (2 equiv.). The reaction mixture was cooled to 0 °C, and HATU (1.5 equiv.) was added. The mixture was then subjected to microwave irradiation at 70 °C for 30 min. After completion, the crude reaction mixture was concentrated under reduced pressure and purified by reverse-phase HPLC (solvent A: water containing 0.05% formic acid (FA); solvent B: acetonitrile (ACN) containing 0.05% FA; gradient 10–80% B over 30 min, held at 80% B for 5 min, then increased to 100% B over 10 min), affording **6** as a white lyophilized powder (27%).

The NMR spectroscopic data were consistent with those previously reported[2,27].

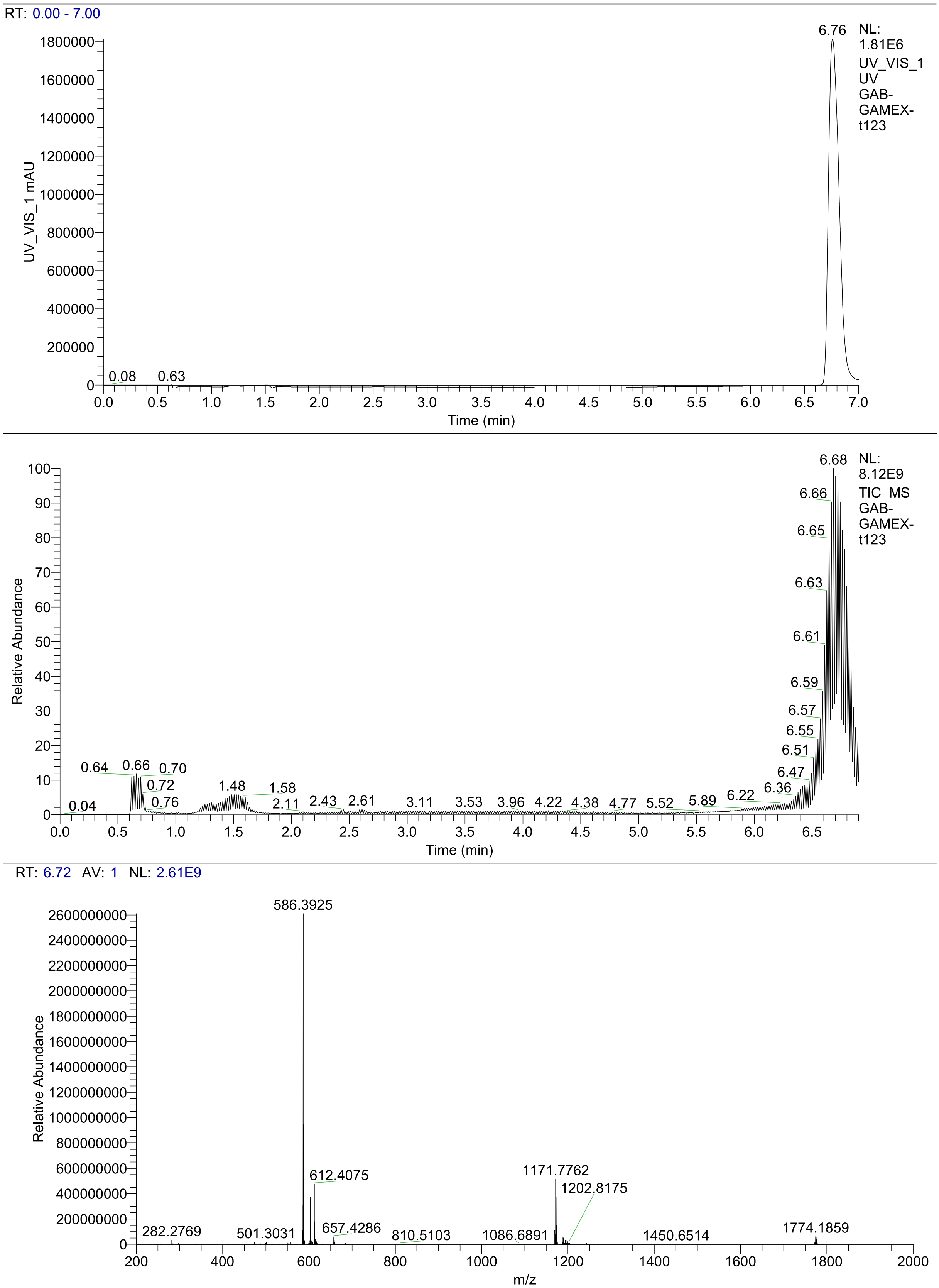

Supplementary Figure 18. HRMS (ESI+) spectrum and UV chromatogram of **6.**

Supplementary Method 2. Biochemical Characterization of AI-2 and WT GxpS_T3 variants

Cloning:

The genes encoding the proteins WT-ATC, WT-AT, WT-TC, and WT GxpS_T3 were amplified from the plasmid pJW76_pCOLA_ara_tacI_II_GxpS using the primer pairs 1/2, 1/3, 4/2, and 4/3, respectively. Corresponding AI-modified variants (AI-ATC, AI-AT, AI-TC, and AI-T3) were amplified from the plasmid pJW76_mod_sacB using the primer pairs 1/2, 1/5, 4/2, and 4/5, respectively. The vector backbone was amplified from a pET28a vector with primers 3 and 4. The resulting fragments were assembled by Gibson assembly to generate the final constructs. In each construct, the expressed protein contains an N-terminal hexa-histidine tag followed by a TEV protease site. The exact sequence added at the N-terminal of each protein is, MGSSHHHHHHSSGENLYFQG.

Expression and solubility test:

For checking the protein expression, the plasmids were transformed into *E. coli* BL21 Star (DE3) and plated on LB agar containing 30 µg mL^{-1} kanamycin, followed by overnight at 37 °C. Single colonies were used to inoculate 2 mL secondary cultures (1% inoculum), which were grown in glass tubes at 37 °C and 130 rpm, until the OD600 reached 0.6–0.8. Cultures were then cooled to 18 °C and induced with isopropyl-β-d-thiogalactopyranoside (IPTG) at final concentrations of 0.02, 0.05, 0.1 and 0.2 mM and grown further for 16 h at 18 °C and 130 rpm. The OD_{600} values of induced and uninduced samples were normalized prior to analysis of protein expression using SDS-PAGE.

Protein solubility was assessed using a similar protocol. Briefly, 50 mL of cultures were induced with 0.05 mM IPTG and grown for 16 h at 18 °C and 130 rpm. Cells were harvested by centrifugation and resuspended in an ice-cold lysis buffer (buffer A) containing 50 mM Tris pH 8.0, 300 mM NaCl, 5% glycerol. Cell densities were normalized, and lysis was performed by sonication (BANDELIN SONOPLUS; MS72 microtip probe; at 50% Amplitude with pulse of 1 sec on and 8 sec off and total 15 min). Unlysed cells were removed by centrifugation at 6,000 × g for 10 min and the remaining lysate was centrifuged at 20,000 × g. The resulting supernatant (soluble fraction) and pellet (insoluble fraction) were separated and analyzed by SDS-PAGE.

Protein expression and purification:

Large-scale purification of AI-T3 was performed from the soluble fraction, whereas WT GxpS_T3 was purified from the insoluble fraction. Two liters of LB broth supplemented with 30 µg mL^{-1} kanamycin was inoculated with a 1% (v/v) overnight *E. coli* culture and grown at 37 °C with shaking at 130 rpm to an OD_{600} of approximately 0.8. Protein expression was induced with IPTG, followed by harvesting at 6000 × g for 10 min and storage of cell pellets at -20 °C until further use.

For AI-T3, expression was induced with 0.2 mM IPTG and cultures were incubated at 18 °C and 130 rpm for 16 h. Thawed cell pellets were resuspended in 40 mL of ice-cold buffer A supplemented with a tablet of protease inhibitors (cOmplete™ EDTA-free Protease-Inhibitor-Cocktail, Roche). Cells were lysed by sonication (BANDELIN SONOPLUS, TS 113 probe) at 58% Amplitude using 1 s on/8 s off pulses for a total time of 30 min. Cell debris were removed by centrifugation at 30,000 × g for 1 h. The clarified supernatant was supplemented with 20 mM imidazole and incubated with 2 mL of HisPure™ Ni-NTA resin (Thermo Fischer Scientific) for 30 min with gentle mixing. The resins were washed with 200 mL of buffer A containing 20 mM Imidazole, and bound protein was eluted using buffer A supplemented with 300 mM

imidazole. Eluted fractions were concentrated by 10 kDa molecular weight cut-off Amicon® Ultra Centrifugal Filters (Merck) and further purified using size-exclusion chromatography (SEC) on a Superdex 200 Increase 100/30 GL column with buffer A. Peak fractions from SEC were pooled, concentrated, flash frozen in liquid nitrogen and stored at -80 °C until further use.

For WT GxpS_T3 expression, expression was induced with 1 mM IPTG followed by incubation at 37 °C and 130 rpm for 3 h. Cell lysis was performed as described above. Insoluble material was collected by centrifugation at 30,000 × g for 1 h and the proteins were extracted using 20 mL buffer A supplemented with 8 M urea with gentle shaking for 2 h at room temperature (rt). The extracted proteins were clarified by centrifugation at 30,000 × g for 1 h. Subsequent purification steps were identical to those used for AI-T3 except all the buffers additionally contained 6 M urea. Eluted fractions were concentrated and stored at rt until further use.

Protein refolding:

The purified proteins were refolded using either a rapid or a gradual refolding approach. Rapid refolding was achieved by immediate removal of urea through SEC, in which protein was loaded onto a Superdex 200 Increase 100/30 GL column equilibrated with buffer A. In contrast, slow refolding was performed by stepwise dialysis. For this method, the eluted protein, initially in a buffer composed of 50 mM Tris pH 8.0, 300 mM NaCl, 5% glycerol, 300 mM imidazole and 6M urea, was dialyzed sequentially against buffer containing 50 mM Tris pH 8.0, 300 mM NaCl, 5% glycerol, 0.4 M L-Arginine, and decreasing concentrations of urea (4 M, 2 M, 1 M, and 0 M). Following dialysis, the protein was concentrated and subjected to SEC with buffer A.

Thermal denaturation and melting temperature (T_m) determination:

Protein samples were prepared at a final concentration of approximately 30–40 µM in buffer A. Samples were centrifuged at 20,000 × g for 15 min to remove particulates prior to measurements. Aliquots of approximately 10 µL were loaded into the capillaries (Prometheus standard capillaries, Nanotemper), ensuring the absence of air bubbles.

Thermal denaturation experiments were performed using a Prometheus Panta nanoDSF instrument (NanoTemper Technologies, 2023) controlled by Panta control software (v1.9). Intrinsic protein fluorescence was monitored as a function of temperature, measuring emission at 330 nm and 350 nm, upon excitation at 280 nm. Samples were heated from 25 °C to 100 °C with a constant heating rate of 3.0 °C min^{-1}. Fluorescence signals were recorded continuously throughout the temperature ramp. Each condition was measured in three technical replicates.

Thermal unfolding curves were analyzed using Panta analysis software (v1.8). The ratio of fluorescence intensities (350nm/330nm) was calculated and plotted as a function of temperature. The apparent T_m was determined as the inflection point of the unfolding transition, obtained by the first derivative curve. Data are presented as mean ± SD of three biological replicates where each biological replicate represents the mean of three technical measurements.

List of primers:

1. 5'-GAGAATCTTTATTTTCAGGGCGTATGTGTCCATCAGTTGTTTG-3'
2. 5'-GTTAGCAGCCGGATCTCACGCCTGCACCAAACTTGC-3'
3. 5'-GTTAGCAGCCGGATCTCACAAAGATTGGGCCAATAC-3'
4. 5'-GAGAATCTTTATTTTCAGGGCGGTTTCCGCATTGAGCCG-3'
5. 5’-GTTAGCAGCCGGATCTCATAACCGTGTCGCCAGTTC-3’
6. 5’-TGAGATCCGGCTGCTAACAAAG-3’
7. 5’-GCCCTGAAAATAAAGATTCTCGCC-3’

Supplementary References

1 Bozhueyuek, K. A. J., Watzel, J., Abbood, N. & Bode, H. B. Synthetic Zippers as an Enabling Tool for Engineering of Non-Ribosomal Peptide Synthetases. *Angew Chem Int Ed Engl* **60**, 17531–17538 (2021).

2 Nollmann, F. I. *et al.* Insect-specific production of new GameXPeptides in *Photorhabdus luminescens* TTO1, widespread natural products in entomopathogenic bacteria. *Chembiochem* **16**, 205–208 (2015).

3 Abbood, N., Duy Vo, T., Watzel, J., Bozhueyuek, K. A. J. & Bode, H. B. Type S Non-Ribosomal Peptide Synthetases for the Rapid Generation of Tailormade Peptide Libraries. *Chemistry – A European Journal* **28**, e202103963 (2022).

4 Ohlendorf, B., Simon, S., Wiese, J. & Imhoff, J. F. Szentiamide, an N-formylated cyclic depsipeptide from *Xenorhabdus szentirmaii* DSM 16338T. *Nat Prod Commun* **6**, 1247–1250 (2011).

5 Hayes, T. *et al.* Simulating 500 million years of evolution with a language model. *Science* **387**, 850–858 (2025).

6 Alamdari, S. *et al.* Protein generation with evolutionary diffusion: sequence is all you need. Preprint at https://doi.org/10.1101/2023.09.11.556673 (2023).

7 Dauparas, J. *et al.* Robust deep learning-based protein sequence design using ProteinMPNN. *Science* **378**, 49–56 (2022).

8 Waterhouse, A. *et al.* SWISS-MODEL: homology modelling of protein structures and complexes. *Nucleic Acids Res* **46**, W296–W303 (2018).

9 Reimer, J. M. *et al.* Structures of a dimodular nonribosomal peptide synthetase reveal conformational flexibility. *Science* **366**, eaaw4388 (2019).

10 Bozhuyuk, K. A. J. *et al.* Evolution-inspired engineering of nonribosomal peptide synthetases. *Science* **383**, eadg4320 (2024).

11 Finn, R. D., Clements, J. & Eddy, S. R. HMMER web server: interactive sequence similarity searching. *Nucleic Acids Res* **39**, W29–W37 (2011).

12 Paysan-Lafosse, T. *et al.* InterPro in 2022. *Nucleic Acids Res* **51**, D418–D427 (2023).

13 Lin, Z. *et al.* Evolutionary-scale prediction of atomic-level protein structure with a language model. *Science* **379**, 1123–1130 (2023).

14 Zhang, Y. & Skolnick, J. Scoring function for automated assessment of protein structure template quality. *Proteins* **57**, 702–710 (2004).

15 Zhang, Y. & Skolnick, J. TM-align: a protein structure alignment algorithm based on the TM-score. *Nucleic Acids Res* **33**, 2302–2309 (2005).

16 Jelinek, F., Mercer, R. L., Bahl, L. R. & Baker, J. K. Perplexity—a measure of the difficulty of speech recognition tasks. *J Acoust Soc Am* **62**, S63 (1977).

17 ESMTeam. ESM Cambrian: Revealing the mysteries of proteins with unsupervised learning. https://evolutionaryscale.ai/blog/esm-cambrian (2024).

18 Jiang, K. *et al.* Rapid in silico directed evolution by a protein language model with EVOLVEpro. *Science* **387**, eadr6006 (2025).

19 Meier, J. *et al.* Language models enable zero-shot prediction of the effects of mutations on protein function. Preprint at https://doi.org/10.1101/2021.07.09.450648 (2021).

20 Schmirler, R., Heinzinger, M. & Rost, B. Fine-tuning protein language models boosts predictions across diverse tasks. *Nat Commun* **15**, 7407 (2024).

21 Hu, E. J. *et al.* LoRA: Low-Rank Adaptation of Large Language Models. Preprint at https://arxiv.org/abs/2106.09685 (2021).

22 Zhao, J., Zhang, C. & Luo, Y. Contrastive Fitness Learning: Reprogramming Protein Language Models for Low-*N* Learning of Protein Fitness Landscape. Preprint at https://doi.org/10.1101/2024.02.11.579859 (2024).

23 Wu, N. C., Dai, L., Olson, C. A., Lloyd-Smith, J. O. & Sun, R. Adaptation in protein fitness landscapes is facilitated by indirect paths. *eLife* **5**, e16965 (2016).

24 Dallago, C. *et al*. FLIP: Benchmark tasks in fitness landscape inference for proteins. Preprint at https://doi.org/10.1101/2021.11.09.467890 (2021).
25 Gray, V. E., Hause, R. J., Luebeck, J., Shendure, J. & Fowler, D. M. Quantitative Missense Variant Effect Prediction Using Large-Scale Mutagenesis Data. *Cell Syst* **6**, 116–124.e3 (2018).
26 Notin, P. *et al*. ProteinGym: Large-Scale Benchmarks for Protein Design and Fitness Prediction. Preprint at https://doi.org/10.1101/2023.12.07.570727 (2023).
27 Bode, H. B. *et al*. Determination of the absolute configuration of peptide natural products by using stable isotope labeling and mass spectrometry. *Chemistry – A European Journal* **18**, 2342–2348 (2012).

Uncropped Gels for Supplementary Figure 6

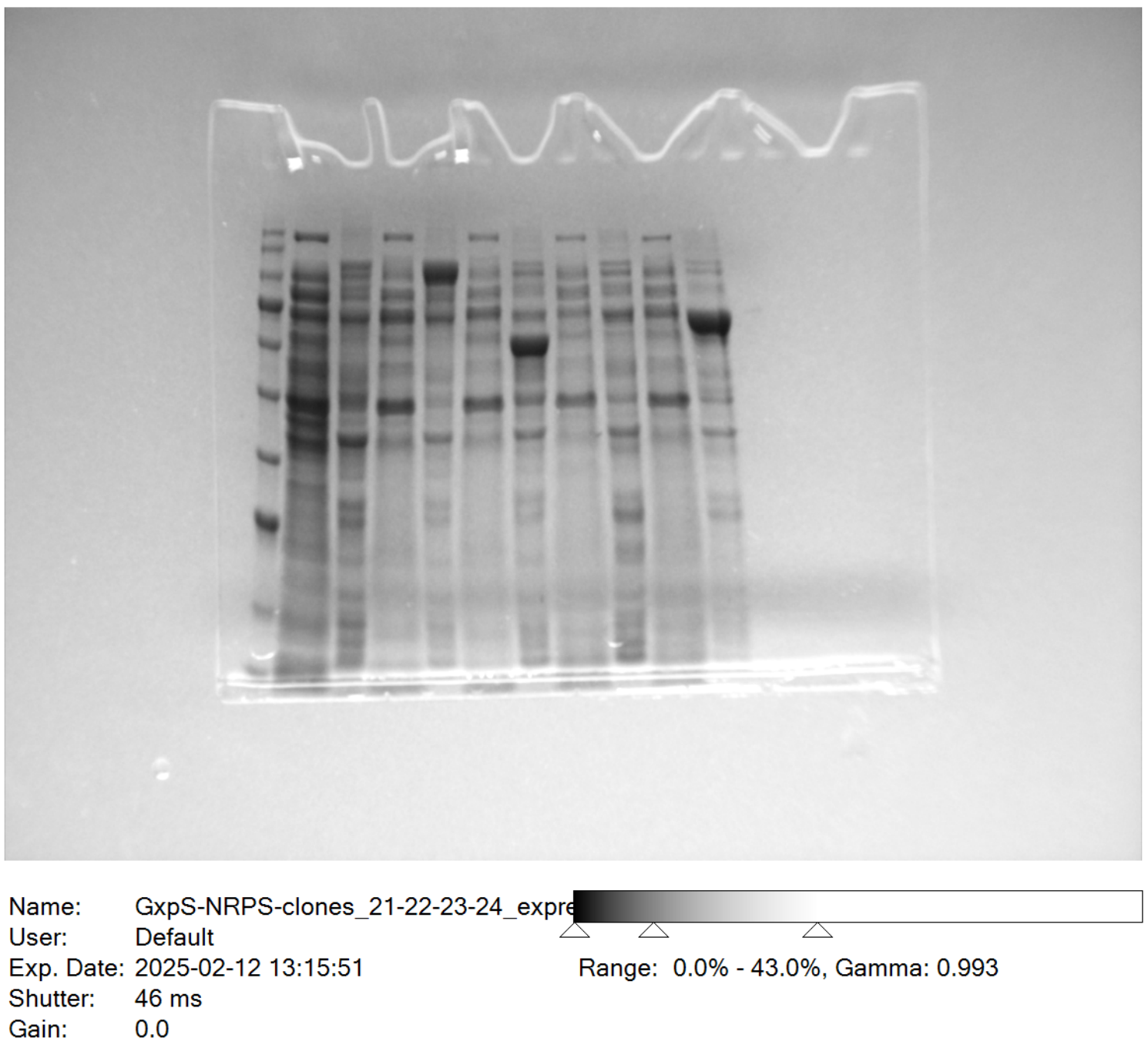

Uncropped gel corresponding to Supplementary Fig. 6a.

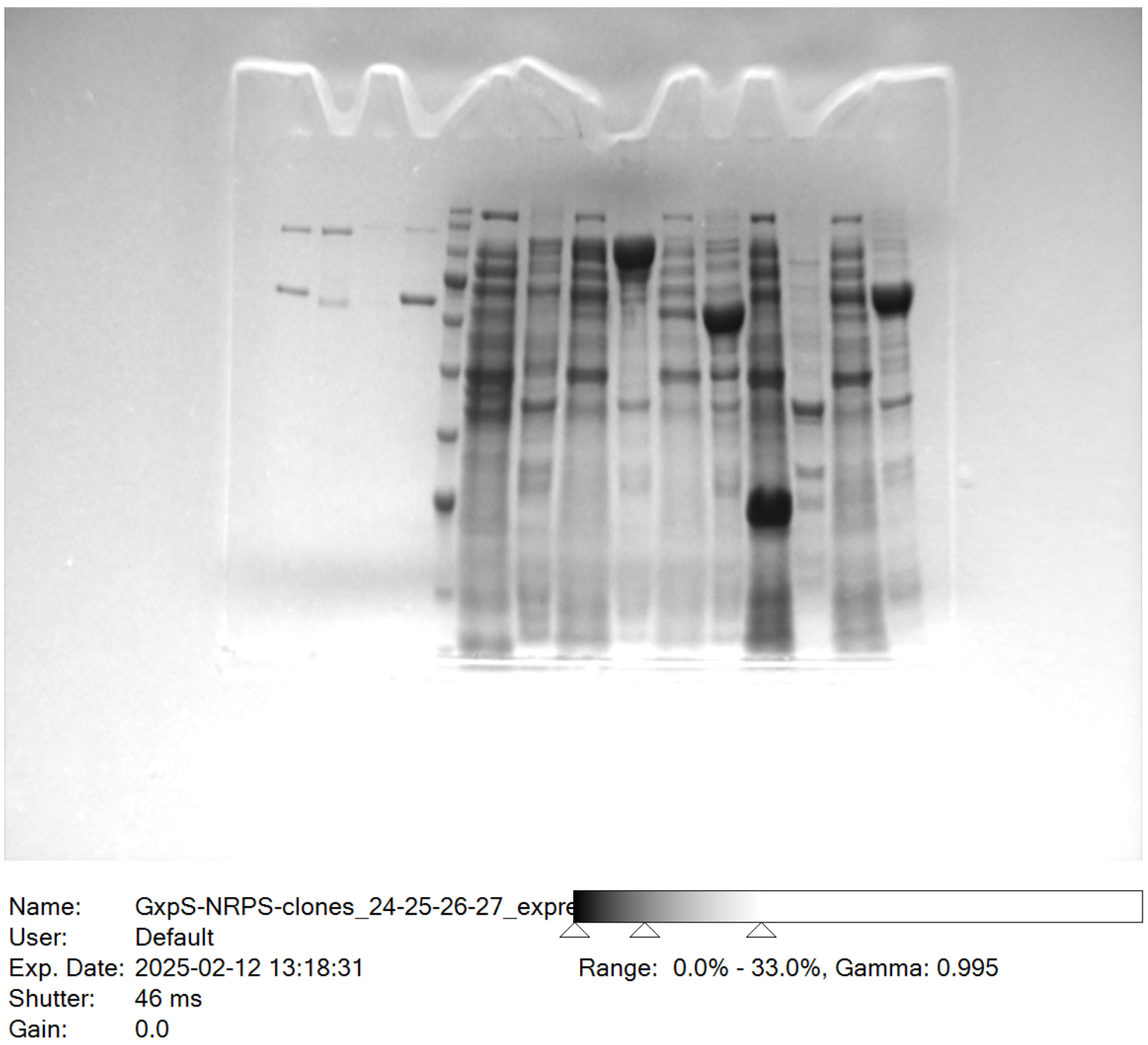

Uncropped gel corresponding to Supplementary Fig. 6b.
