# Supplementary material for: Generative AI designs functional thiolation domains for reprogramming non-ribosomal peptide synthetases
Source: Nat Commun. 2026 Sep 22;17:10084. doi: 10.1038/s41467-026-77963-6 (PMC13598123; doi:10.1038/s41467-026-77963-6)
Supplement: Supplementary file 2 [file 41467_2026_77963_MOESM2_ESM.pdf]

Description of Additional Supplementary Files

Supplementary Data 1: Supplementary Data file containing amino-acid sequences and production data, constructs, primers, strains, phylogenetic-analysis data, and MD contact-analysis data.
