# Supplementary material for: Generative AI designs functional thiolation domains for reprogramming non-ribosomal peptide synthetases
Source: Nat Commun. 2026 Sep 22;17:10084. doi: 10.1038/s41467-026-77963-6 (PMC13598123; doi:10.1038/s41467-026-77963-6)
Supplement: Supplementary file 3 [file 41467_2026_77963_MOESM4_ESM.pdf]

Corresponding author(s): Kenan A. J. Bozhüyük

Last updated by author(s): Aug 11, 2026

Reporting Summary

Nature Portfolio wishes to improve the reproducibility of the work that we publish. This form provides structure for consistency and transparency in reporting. For further information on Nature Portfolio policies, see our Editorial Policies and the Editorial Policy Checklist.

Statistics

For all statistical analyses, confirm that the following items are present in the figure legend, table legend, main text, or Methods section.

n/a	Confirmed	
☐	☒	The exact sample size (n) for each experimental group/condition, given as a discrete number and unit of measurement
☐	☒	A statement on whether measurements were taken from distinct samples or whether the same sample was measured repeatedly
☐	☒	The statistical test(s) used AND whether they are one- or two-sided<br>*Only common tests should be described solely by name; describe more complex techniques in the Methods section.*
☒	☐	A description of all covariates tested
☒	☐	A description of any assumptions or corrections, such as tests of normality and adjustment for multiple comparisons
☐	☒	A full description of the statistical parameters including central tendency (e.g. means) or other basic estimates (e.g. regression coefficient) AND variation (e.g. standard deviation) or associated estimates of uncertainty (e.g. confidence intervals)
☐	☒	For null hypothesis testing, the test statistic (e.g. F, t, r) with confidence intervals, effect sizes, degrees of freedom and P value noted<br>*Give P values as exact values whenever suitable.*
☒	☐	For Bayesian analysis, information on the choice of priors and Markov chain Monte Carlo settings
☒	☐	For hierarchical and complex designs, identification of the appropriate level for tests and full reporting of outcomes
☐	☒	Estimates of effect sizes (e.g. Cohen's d, Pearson's r), indicating how they were calculated

Our web collection on statistics for biologists contains articles on many of the points above.

Software and code

Policy information about availability of computer code

Data collection	Agilent RapidFire software (G9532A) was used for RapidFire LC-MS data acquisition. Bruker Compass HyStar v5.1 and Thermo Scientific Xcalibur 4.5.474.0 were used for LC-MS/MS acquisition. Panta Control software (v1.9) was used for nanoDSF measurements.
Data analysis	Data analysis used MZmine v4.0.367, EESM3 1.4B, EvoDiff 640M OADM, ProteinMPNN v_48_002, ESMFold, ESM2 650M, ESMC, scikit-learn v1.4.2, MODELLER v10.5, MOE 2024.0601, AMBER24/cpptraj, Geneious Prime v2026.0.2, Bruker Compass DataAnalysis v5.3, Thermo Scientific Xcalibur 4.5.474.0, IQ-TREE v2.0.7 and iTOL v7. Custom code for generative design, surrogate modeling and downstream analyses is available via the GitHub repository listed in the manuscript.

For manuscripts utilizing custom algorithms or software that are central to the research but not yet described in published literature, software must be made available to editors and reviewers. We strongly encourage code deposition in a community repository (e.g. GitHub). See the Nature Portfolio guidelines for submitting code & software for further information.

Data

Policy information about availability of data

All manuscripts must include a data availability statement. This statement should provide the following information, where applicable:

- Accession codes, unique identifiers, or web links for publicly available datasets
- A description of any restrictions on data availability
- For clinical datasets or third party data, please ensure that the statement adheres to our policy

Data availability: All plasmids, primers, strains, construct sequences and peptide lists are provided in Supplementary Data 1. Annotated GenBank files (.gb) for the plasmid constructs used in this study are provided in the Source Data. Source data are provided with this paper. Protein structures used in this study include Protein Data Bank (PDB) entries 5T3D (https://doi.org/10.2210/pdb5T3D/pdb), 6MFY (https://doi.org/10.2210/pdb6MFY/pdb), 6MFZ (https://doi.org/10.2210/pdb6MFZ/pdb), 6MG0 (https://doi.org/10.2210/pdb6MG0/pdb), 8JBR (https://doi.org/10.2210/pdb8JBR/pdb), and 9BE3 (https://doi.org/10.2210/pdb9BE3/pdb). Molecular dynamics simulation trajectories, associated input files, and raw LC–MS data are available from the corresponding author upon request due to their large file size. Code availability: Custom code was used for generative design, surrogate modeling, candidate prioritization and downstream analyses, as described in the Methods and Supplementary Methods. The code is available via GitHub (https://github.com/uds-lsv/T-domain-design) and has been archived in Zenodo (https://doi.org/10.5281/zenodo.21829302). All third-party software and relevant parameters are detailed in the Methods and Supplementary Methods.

Research involving human participants, their data, or biological material

Policy information about studies with human participants or human data. See also policy information about sex, gender (identity/presentation), and sexual orientation and race, ethnicity and racism.

Reporting on sex and gender	n.a.
Reporting on race, ethnicity, or other socially relevant groupings	n.a.
Population characteristics	n.a.
Recruitment	n.a.
Ethics oversight	n.a.

Note that full information on the approval of the study protocol must also be provided in the manuscript.

Field-specific reporting

Please select the one below that is the best fit for your research. If you are not sure, read the appropriate sections before making your selection.

☒ Life sciences ☐ Behavioural & social sciences ☐ Ecological, evolutionary & environmental sciences

For a reference copy of the document with all sections, see nature.com/documents/nr-reporting-summary-flat.pdf

Life sciences study design

All studies must disclose on these points even when the disclosure is negative.

Sample size	Sample sizes are reported in the relevant figure legends, Methods, Supplementary Methods and Supplementary Data. Most experimental comparisons were performed with n = 3 independent biological replicates. Thermal unfolding measurements used 3 biological replicates, each summarized from 3 technical replicates. Variant-set sizes are reported explicitly where relevant. MD analyses used 2 independent simulations per construct/state. Phylogenetic analysis used 129 sequences. No formal power calculation was performed.
Data exclusions	No data was excluded.
Replication	Key findings were reproduced across biological replicates, and major conclusions were supported across multiple constructs, assay formats and NRPS architectures.
Randomization	The experiments were not randomized. Randomization was not relevant to this study: samples are not allocated to experimental groups, since each group is defined by the identity of the engineered construct itself. Constructs were assayed in parallel under identical conditions, with wild-type controls included in every batch.
Blinding	The investigators were not blinded to allocation during experiments and outcome assessment. Blinding was not relevant to this study: group allocation corresponds to the identity of the DNA construct being assayed and is therefore necessarily known during cloning, expression and measurement. All readouts were quantitative and instrument-derived (RapidFire-MS and LC-MS peak areas, nanoDSF thermal unfolding curves, SEC traces) and were processed with identical, pre-defined parameters across all samples, so no subjective assessment step exists at which investigator expectation could influence the outcome.

Reporting for specific materials, systems and methods

We require information from authors about some types of materials, experimental systems and methods used in many studies. Here, indicate whether each material, system or method listed is relevant to your study. If you are not sure if a list item applies to your research, read the appropriate section before selecting a response.

Materials & experimental systems

n/a	Involved in the study
☒	☐ Antibodies
☒	☐ Eukaryotic cell lines
☒	☐ Palaeontology and archaeology
☒	☐ Animals and other organisms
☒	☐ Clinical data
☒	☐ Dual use research of concern
☒	☐ Plants

Methods

n/a	Involved in the study
☒	☐ ChIP-seq
☒	☐ Flow cytometry
☒	☐ MRI-based neuroimaging

Plants

Seed stocks	n.a.
Novel plant genotypes	n.a.
Authentication	n.a.
