# Supplementary material for: Generative AI designs functional thiolation domains for reprogramming non-ribosomal peptide synthetases
Source: Nat Commun. 2026 Sep 22;17:10084. doi: 10.1038/s41467-026-77963-6 (PMC13598123; doi:10.1038/s41467-026-77963-6)
Supplement: Supplementary file 4 [file 41467_2026_77963_MOESM5_ESM.pdf]

nature portfolio

Peer Review File

Generative AI designs functional thiolation domains for reprogramming non-ribosomal peptide synthetases

Corresponding Author: Dr Kenan Bozhüyük

This file contains all reviewer reports in order by version, followed by all author rebuttals in order by version.

Version 0:

Reviewer comments:

Reviewer #1

(Remarks to the Author)
This manuscript presents a valuable application of generative AI for engineering thiolation (T) domains in nonribosomal peptide synthetases (NRPSs), demonstrating enhanced catalytic activity and hybrid NRPS functionality. High-throughput T-domain replacement was achieved using the Type S GxpS platform. Sixty-six AI-designed variants were screened in the minimal SU2-only system; variant AI-27, for instance, exhibited 163% of wild-type (WT) activity in this context. Functionality was further validated in the complete assembly line, where AI-27 reached 257% of WT activity. Molecular dynamics simulations revealed that AI-designed T-domains (e.g., AI-2) reorganize state-dependent interfacial contact networks while maintaining global folding stability. Domain transplantation and hybrid NRPS construction showed that functional compatibility is primarily governed by the downstream condensation domain type (LCL-type vs. C/E-type).
Overall, the manuscript is interesting, but I have several concerns:
1 The manuscript mentions using ESM3, ProteinMPNN, and EvoDiff for T-domain design under "local-context constraints" (flanking A/C domains). However, how were the "structural tokens" for ESM3 or "backbone coordinates" for ProteinMPNN specifically defined and validated? Were multiple structural templates tested, and how did template choice affect design outcomes?
2 How was "representative sampling" performed in Round 1? Please specify the clustering algorithm, distance threshold, and sampling strategy (e.g., maximum diversity), and explain why failed ProteinMPNN variants (e.g., AI-5) were excluded from diagnostic analysis.
3 In Figure 3, WT activity fluctuates 17-fold across different media. Why was "% WT activity" reported under non-standardized conditions? Was analysis of covariance performed to rule out culture condition interference with interfacial effect conclusions?
4 What is the statistical basis for the "contact reorganization" conclusion in MD simulations? Please provide significance tests (e.g., t-test p-values) for contact occupancy differences, the number of simulation replicates, and discuss reliability risks associated with templates showing 22–35% sequence identity.
5 For the LC-MS data of the hybrid NRPS system, why are only relative peak areas reported? Please explain the mechanistic hypothesis for why AI-12 succeeded in GshS (Figure 7b) but failed in GxhS-1 (Figure 7a). Was interference from upstream A-domain interactions considered?
6 The Tm value of AI-2 (~52°C) is significantly higher than that of WT (~40°C), but WT was denatured, purified, and refolded. Has consistency between refolded WT (e.g., SEC-refolded) and native WT been validated? Does the enhanced thermal stability fail at non-T3 positions (e.g., T1/T2)?
The study focuses on the GxpS system, leaving broader applicability unclear. Are the "downstream C-domain compatibility rules" (LCL-type vs. C/E) generalizable to other NRPS families? Testing in a distantly related NRPS (e.g., from Streptomyces or fungi) would strengthen claims of "context-conditioned optimization." Additionally, the "positional dependence" (Fig. 6) shows variable performance across T1–T5 positions, but the structural basis (e.g., sequence motifs unique to T5) is not mechanistically explained.

(Remarks on code availability)

Reviewer #2

(Remarks to the Author)

The manuscript submitted by Bülbül and colleagues combines generative protein design approaches (ESM3, ProteinMPNN, EvoDiff) with iterative design cycles to generate de novo thiolation (T) domains for non-ribosomal peptide synthetases (NRPSs). They experimentally evaluate a large number of variants, showing that a substantial fraction of AI-designed T-domains support peptide production and can, in some cases, outperform native domains. I consider that the topic and experimental scope is well chosen and is of interest for Nat Commun readers. However, some key aspects require clarification or strengthening, all of them listed here without any specific order of priority:

•While the manuscript emphasizes context-aware generative design, the actual pipeline appears closer to generate → filter → experimentally screen than to predictive, mechanism-driven design. For me, there is no direct evidence that the models a priori encode or optimize interdomain interface features. I consider that the authors could provide in silico evidence that selected designs are enriched in interface-relevant featuress.

•I recognize the important number of variants that were tested but it is unclear for example how many sequences were initially generated per round, what fraction was filtered out prior to experimental testing, whether selection criteria introduce bias toward sequences already close to functional? This makes it difficult to assess the true success rate of the approach.

•The improved developability of AI-2 (higher soluble expression, efficient refolding, and also higher Tm) is compelling. Howeve I think that the analysis is limited to a single design and it is unclear whether this improvement is representative of AI-designed sequences correlated with functional output. Given the central importance of expression and solubility in NRPS engineering, this is a key gap. I don't know if the authors analyzed a possible correlation between expression/solubility vs thermal stability?

•Stability claims are primarily based on thermal unfolding (Tm) and SEC-based refolding behavior. While informative, these measurements do not fully capture protein stability. Given that stability is used to support mechanistic interpretations, the current level of characterization is somewhat limited and might be rephrased to make clear that the experimental approach is not supporting completely a whole conformational stability analysis.

•MD simulations suggest differences in interdomain contact networks between WT and AI-2. However these observations remain correlative and no experimental validation is provided (eg. mutational testing)… Thus, the mechanistic conclusions drawn from MD are suggestive but not strongly supported. Although experimentally test key MD-derived hypotheses (eeg., targeted mutations disrupting predicted contacts) would be a lot informative, I know that's a lot of work and maybe a different manuscript, so I suggest tone down mechanistic claims.

•Clarify the operational definition of "functional" (detectable vs above threshold).

•I think that the term "context-conditioned design" may be overstated and could be moderated.

•I'm not an expert in LC-MS/MS, but since the manuscript relies heavily on this, could the authors clarify how variability in ionization efficiency across different peptide products is controlled for? In particular, how robust are comparisons across variants producing structurally related but non-identical peptides?

•The authors state that round 1 designs were evaluated "out-of-the-box" without in silico filtering beyond sequence clustering and representative sampling. It would be helpful to clarify what criteria were used for clustering and selection of representative sequences. In particular, what sequence identity thresholds or clustering algorithms were applied? How many sequences were initially generated, and how many were retained after clustering? I was wondering if this step could have implicitly biased the selection toward more "natural-like" or higher-likelihood sequences? This is particularly relevant for interpreting the apparent success of ESM3 designs in round 1, as even minimal preprocessing may substantially enrich for functional sequences relative to a fully unfiltered generative output.

•The authors extend their analysis by testing whether AI-designed T-domains can function as interchangeable parts in hybrid NRPS systems, which is an important and practically relevant question. Maybe I'm wrong but the observed outcomes appear highly context-dependent, with strong variation across hybrid systems and downstream partner domains. This raises the question of whether the current approach enables predictive engineering, or whether it still relies on empirical screening of multiple candidates.

•In this regard, for me it is not entirely clear how transferable individual designs are across different NRPS architectures; for example: do top-performing variants in one hybrid context tend to perform well in others? I think the manuscript would benefit from a more explicit discussion of the limitations of using AI-designed domains as modular engineering parts. For example in particular to what extent can these domains be considered reusable components versus context-specific solutions that require re-optimization for each new assembly line?

•The authors use mean pLDDT scores from ESMFold as a proxy for foldability of generated sequences. While pLDDT is a useful indicator of model confidence in predicted structures, its interpretation as a direct measure of protein foldability or stability warrants further clarification. Could the authors clarify how well mean pLDDT correlates with actual folding, solubility, or stability in their dataset? It would strengthen the manuscript to include an analysis of whether pLDDT scores correlate with experimental outcomes.

•Additionally, using the mean pLDDT across the entire sequence may obscure local regions of low confidence (maybe interface-relevant residues or flexible loops). Have the authors considered residue-level or region-specific analyses,

particularly at interdomain interfaces?

(Remarks on code availability)
I did not independently install or execute the code as part of this review. However, based on the description provided in the manuscript, the computational workflow appears to be reasonably well outlined at a conceptual level

Reviewer #3

(Remarks to the Author)
In their article 'Generative AI designs functional thiolation domains for reprogramming nonribosomal peptide synthetases', the authors prepared novel thiolation (T) domains with the aid of AI and design-build-test-learn cycles. In the best cases, these non-natural carrier protein domains exhibited better solubility and stability and enabled higher non-ribosomal peptide product yields than their native counterparts.

The authors' system of interest, Type S GxpS, consists of two independently expressed NRPS subunits (domains A1-C3 and domains A3-TE5) that interact via synthetic zippers. Functional interaction between the subunits allows for production of the cyclic compounds 5-6 (primarily 6), whereas a lack of interaction only yields the linear compounds 1-4 (primarily 1 + 2), which are detectable by LC-MS/MS. Variants of domain T3 were inserted into subunit 2 using a Golden Gate strategy. These variants were designed using three generative models (ESM3, EvoDiff and ProteinMPNN), with the neighbouring A4 and C3 domain sequences and/or structures as input and the T3 domain masked.

The authors begam with a minimal (subunit 2-only) NRPS. In the three rounds of testing, the authors analyzed progressively more T3 domain variants for production of 1 and 2. In round 1, only product titer was followed. In round 2, phosphopantetheinylation was ensured by preserving the conserved FFxxGGxS motif, a minimum identify of 50% to wild-type T3 domain (WT GxpS_T3) was imposed, and low perplexity was filtered for. In round 3, sequence-activity measurements from rounds 1 and 2 guided the selection of further candidates. Across the three rounds, the number of variants with moderate activity threshold (≥50 %) increased. The authors found a poor correlation between sequence identity to WT GxpS_T3 and product yield.

Following round 2, error-prone PCR was used to expand the sequence space of three AI-generated T3 domains (AI2, AI-12 and AI15) and WT GxpS_T3, with production of 1 and 2 as readout. Here, the AI-2 scaffold was shown to outperform the others significantly.

Subsequently, the authors turned to the full-length (subunit 1 + subunit 2) NRPS. Here, product 6 could be generated only if T3 variants productively interact with intramodular machinery (A3 and C/E4) as well as intermodular machinery (C3). The eight top-performing variants from the SU2-only experiments were tested and found to produce 6, including two that greatly exceeded the yield of WT. Altering the culture volume (1 to 50 mL) and medium (succinate XPP medium to LB medium) revealed that titers of AI-designed variants were strongly dependent on cultivation conditions.

Next, the authors sought to further characterize AI-2, which was a top performer in the SU2-only assay. Compared to the largely insoluble WT GxpS_T3, AI-2 was found to express solubly, with higher thermal stability, in variously-sized constructs from AI-2 alone to A3–AI-2–C/E4. While AI-2 itself did not give maximal product titers, epPCR diversification of AI-2 led to the highest-yielding variant.

MD simulations of WT GxpS_T3 and AI-2 in three catalytic states (thiolation, condensation donor, condensation acceptor) followed. In each state for both T domains, all domains remained structurally stable, as evaluated by several parameters. Interestingly, interaction interfaces showed substantial differences between WT and variant.

Three T domain variants (AI-2, AI-12, AI-38) were next tested at different positions along the NRPS, taking the place of T1, T2, T4 or T5 for production of 6. As expected, most of these constructs showed loss or abolition of yield, although position T5 was found to be fairly permissive. The authors rationalized this according to downstream C domain partner type.

The authors sought to engineer functional hybrid NRPSs containing their AI-designed T domains. These hybrids consisted of parts from GxpS and XtpS (GxpS-XtpS; GxhS-1) or GxpS and SzeS (GxpS-SzeS; GshS) and introduced non-cognate interaction interfaces. By monitoring production of hybrid compounds 9 and 16, the authors found that T domain variants significantly affected yields: for Gxhs-1, three variants improved production relative to native GxpS_T3, while two abolished production; for GshS, three variants showed improved production relative to native Sze_T4, while two showed reduced production. The authors state that, in general, the hybrids designed using the XUT strategy were more successful. Testing a second GxpS-XtpS hybrid (GxhS-2), in which the fusion site was moved from T3 to T2 (in front of an LCL domain instead of a C/E domain) with T domain variants that were optimized for this C domain type showed either no or markedly improved production of 23 relative to GxpS_T2.

Finally, the authors analyzed product formation after installing 76 AI-designed T domains into GxhS-1, GxhS-2, and GshS and the native SzeS assembly line. They found that downstream C domain type (i.e. LCL vs. C/E) was the most crucial factor in preparing active assembly lines.

In general, I find the manuscript excellent and original. It is well written and the figures are clear. However, before the article is accepted for publication in Nature Communications, I recommend a number of points be addressed.

PRIMARY COMMENTS

- The constructs modeled in MD simulations are unclear in the results section. The authors should explicitly state which domains were present in each catalytic state tested (thiolation, condensation donor, condensation acceptor). Initially, I expected the presence of four domains: upstream C and ATC. Only upon reading the methods section did I guess that the alternative positioning of the T domain on donor vs. acceptor faces of the C domain translates to condensation donor vs. acceptor states.

- I would like to see improved figures showing major domain-domain interactions in the MD simulations (currently, catalytic domains are only displayed as surfaces). These could be included as supplementary figures.

- In the section 'AI-designed T-domains can be leveraged to generate hybrid NRPSs', C domain type could be included in Fig. 4, or at least explicitly mentioned in the text, for easier comparison.

- Also in this section, the authors write, 'To test interface-specific design, T-domains optimized for LCL-type condensation interfaces were evaluated…'. To my knowledge, no detail is given on how these variants were generated compared to the others that were optimized for C/E type interfaces.

- The authors state: 'Notably, improved production was observed in a hybrid assembled via an XUT-style39 insertion architecture (Fig. 7b), showing that AI-designed carrier domains can outperform established evolution-guided NRPS engineering approaches.' I am not fully convinced by this claim. For direct comparison, the authors should prepare a hybrid GxpS-XtpS NRPS using the XUT approach or a hybrid GxpS-SzeS NRPS using the same whole-domain replacement strategy as for GxhS-1.

SECONDARY COMMENTS

Main section
- The authors have written: 'This has given rise to a rapidly expanding toolkit of generative approaches spanning several model classes. (I) Sequence-based foundation models such as ESM311; (II) Diffusion models such as EvoDiff12; and (III) Structure-conditioned design methods such as ProteinMPNN13.'

Please combine these sentences as follows:
This has given rise to a rapidly expanding toolkit of generative approaches spanning several model classes: (I) Sequence-based foundation models such as ESM311; (II) Diffusion models such as EvoDiff12; and (III) Structure-conditioned design methods such as ProteinMPNN13.

Results section
- Consider rewriting 'A3–TE' as 'A3-TE5' for clarity.

- The authors have written: 'Candidate T-domains were proposed using three complementary generative strategies (ESM311, EvoDiff12, ProteinMPNN13), under an explicit local-context constraint: we specified the intended A–T–C neighborhood by providing the flanking A and C domains (and, where applicable, structural context) while masking the T-domain segment during generation, thereby forcing designs to be compatible with the target junction environment (Supplementary Note 1; Supplementary Data 1).'

Please edit this text to state explicitly that you provided amino acid sequences; e.g., '…by providing the amino acid sequences of flanking A and C domains'.

- The following terms would benefit from definitions or simplified descriptions, as they may be foreign to the general reader (including this reviewer!): structure tokens, lightweight in silico filters, data-driven surrogate models.

Fig. 1
- In panel a, module 4 is missing a horizontal bar beneath it.
- Adding labels of SU1 and SU2 to panel a could improve clarity.
- In panel d, the text at the top should read 'heterologous expression in E. coli'

Fig. 2
- The caption description of panel b would benefit from further explanation. While panel b is useful, not all readers will be familiar with violin plots.

Fig. 4
- The x-axis labels in panel d could use clarification in the caption.

Methods section
- The authors write: 'Separations were performed on a Hypersil GOLD C18 column (1.9 µm, 100 × 2.1 mm; Thermo Fisher Scientific) at 40 °C with a flow rate of 0.700 mL min-1 using solvent A (water supplemented with 0.1 % formic acid) and solvent B (acetonitrile supplemented with 0.1 % formic acid) The gradient for tripeptides 1 and 2 was 10 % B (0-0.2 min), 10-90 % B (0.2-0.4 min), 90 % B (0.4-1.2 min), and 10 % B (1.2-1.6 min).' A period is missing between 'formic acid) The

gradient'.

- The authors write: 'Collision-induced dissociation (CID) energies were set to a basic value of 50 and tune collision energy set to star 7 eV through end 200 eV.' I believe 'star' here should be 'start'.

(Remarks on code availability)

Version 1:

Reviewer comments:

Reviewer #1

(Remarks to the Author)
The authors offer comprehensive replies to all questions and specify concrete revisions to the main text and supplementary materials. They transparently acknowledge study limitations and carefully moderate overinterpretation of modeling and screening results. Most technical uncertainties raised have been satisfactorily clarified. A small number of open mechanistic questions are appropriately framed as future work.
Minor room for improvement remains: a few explanations rely on negative evidence or indirect support rather than dedicated control experiments; some proposed future directions could be briefly highlighted within the main text rather than only in the response letter.

(Remarks on code availability)

Reviewer #2

(Remarks to the Author)
The authors have thoroughly addressed the concerns and general questions raised during the previous round of review. The revised manuscript incorporates the requested analyses and clarifications, and I consider that the changes have substantially strengthened the work. I have no further comments and believe that the manuscript is now suitable for publication in Nature Communications.

(Remarks on code availability)

Reviewer #3

(Remarks to the Author)

(Remarks on code availability)

Open Access This Peer Review File is licensed under a Creative Commons Attribution 4.0 International License, which permits use, sharing, adaptation, distribution and reproduction in any medium or format, as long as you give appropriate credit to the original author(s) and the source, provide a link to the Creative Commons license, and indicate if changes were made.
In cases where reviewers are anonymous, credit should be given to 'Anonymous Referee' and the source.
The images or other third party material in this Peer Review File are included in the article's Creative Commons license, unless indicated otherwise in a credit line to the material. If material is not included in the article's Creative Commons license and your intended use is not permitted by statutory regulation or exceeds the permitted use, you will need to obtain permission directly from the copyright holder.
To view a copy of this license, visit https://creativecommons.org/licenses/by/4.0/

Response to the Reviewers – First Revision

Title: Generative AI designs functional thiolation domains for reprogramming non-ribosomal peptide synthetases

Dear Reviewers,

We thank all three reviewers for their careful and constructive assessment of our manuscript. Their comments have helped us clarify the methodology and strengthen the manuscript. We addressed each point individually below. All changes in the manuscript and Supplementary Information are marked in red and below we provide a point-by-point response to the **comments shown in bold** and our responses following in regular text.

We hope that our manuscript is now suitable for publication in Nature Communications and we look forward to your reply.

Best regards,

Kenan Bozhüyük (in the name of all authors)

REVIEWER COMMENTS

Reviewer #1 (Remarks to the Author):

This manuscript presents a valuable application of generative AI for engineering thiolation (T) domains in nonribosomal peptide synthetases (NRPSs), demonstrating enhanced catalytic activity and hybrid NRPS functionality. High-throughput T-domain replacement was achieved using the Type S GxpS platform. Sixty-six AI-designed variants were screened in the minimal SU2-only system; variant AI-27, for instance, exhibited 163% of wild-type (WT) activity in this context. Functionality was further validated in the complete assembly line, where AI-27 reached 257% of WT activity. Molecular dynamics simulations revealed that AI-designed T-domains (e.g., AI-2) reorganize state-dependent interfacial contact networks while maintaining global folding stability. Domain transplantation and hybrid NRPS construction showed that functional compatibility is primarily governed by the downstream condensation domain type (LCL-type vs. C/E-type).

We thank Reviewer 1 for the careful and positive assessment of our manuscript, and we address the specific points raised below.

Overall, the manuscript is interesting, but I have several concerns:

1. The manuscript mentions using ESM3, ProteinMPNN, and EvoDiff for T-domain design under "local-context constraints" (flanking A/C domains). However, how were the "structural tokens" for ESM3 or "backbone coordinates" for ProteinMPNN specifically defined and validated? Were multiple structural templates tested, and how did template choice affect design outcomes?

We have now clarified both the structural conditioning and its validation in Supplementary Note 1 (page 25). For ProteinMPNN, backbone coordinates were taken from a SWISS-MODEL homology model of the GxpS A3–T3–C4 tridomain based on the LgrA structures (PDB 6MFY, 6MFZ, 6MG0); for structure-conditioned ESM3 generation, an AlphaFold3-predicted A3–T3–C4 structure was supplied through the structure track with the T3 region masked. In both cases the flanking A3 and C4 domains were held fixed as conditioning context and only T3 was regenerated. Model quality was assessed prior to design, and both the SWISS-MODEL homology model and the AlphaFold3 model reproduced the canonical T-domain fold with the expected positioning of the conserved catalytic residues, including the phosphopantetheine-accepting serine. These observations supported their use for structure-conditioned sequence generation. We did not perform a systematic benchmark of alternative structural templates, in part because experimentally determined A–T–C multidomain structures suitable as templates are scarce and the LgrA structures represent the principal available reference. We note, however, that structure conditioning was applied through two independent structural sources — a SWISS-MODEL/LgrA model for ProteinMPNN and an AlphaFold3 model for ESM3 — and that both routes yielded functional designs, while sequence-only routes (ESM3 without structural conditioning and EvoDiff) also produced functional T-domains. This indicates that the approach does not depend on a single template or structural input, although a systematic analysis of template choice remains an interesting direction for future work.

2. How was "representative sampling" performed in Round 1? Please specify the clustering algorithm, distance threshold, and sampling strategy (e.g., maximum diversity), and explain why failed ProteinMPNN variants (e.g., AI-5) were excluded from diagnostic analysis.

Round 1 was designed as a deliberately exploratory, out-of-the-box assessment without activity prediction, perplexity filtering, structural ranking, or surrogate-model guidance. We generated 100 sequences each with ESM3 and ProteinMPNN. No distance or sequence-identity threshold or automated sampling algorithm was applied. From the 200 generated sequences, we selected 11 candidates (AI-1–AI-11) by manual inspection. The

selection was guided by two criteria: representation of both generative models and coverage of the diversity of the phosphopantetheinylation core motif, specifically the residue immediately preceding the catalytic serine. To visualize representative sampling of the generated sequence space, all 200 sequences were embedded using ESM2 and visualized by k-means clustering, PCA and UMAP. The clustering was used only to aid visualization and did not determine candidate selection. All ESM3 designs retained the canonical His–Ser motif (GGHS), whereas the ProteinMPNN designs varied the residue at this position. We therefore selected the four ESM3 designs (AI-1–AI-4) to sample sequence diversity within the conserved GGHS background and one ProteinMPNN design for each of the major core-motif variants generated (His in AI-7, and the non-canonical Ala-, Gly-, Glu-, Asn-, Asp-, and Thr-containing contexts in AI-5, AI-6, AI-8, AI-9, AI-10, and AI-11), thereby representing the majority of the motif variation produced by ProteinMPNN. This intentionally unfiltered, motif-spanning design provided an unbiased assessment of out-of-the-box model performance while probing tolerance at this functionally important position. The ESM2 embedding of the generated sequence pool, visualized by k-means clustering (with the number of clusters estimated using the elbow method), PCA, and UMAP, has now been added as **Supplementary Fig. S17**, and the selection procedure is described in detail in **Supplementary Note 1 ("Candidate selection across design rounds")**.
None of the ProteinMPNN variants were excluded from analysis: all seven variants (AI-5–AI-11) are reported, five of which were non-functional. Their behaviour is best understood in the context of the residue immediately preceding the invariant phosphopantetheinylation serine (position X in the FFxxGGxS motif), which is known to correlate with downstream C-domain identity. In natural T-domains this position is occupied predominantly by histidine, aspartate or asparagine, with histidine being characteristic of GxpS_T3. Glutamate, glycine and threonine are usually not observed at this position in natural T-domains. Consistent with this, the functional designs retained a naturally occurring residue (histidine in the four ESM3 variants and AI-7; aspartate in AI-10), whereas the non-functional designs introduced non-natural residues (glutamate in AI-8/GGES, glycine in AI-6/GGGS, and threonine in AI-11/GGTS) or, in AI-5, shifted the motif register through an inserted residue (GGHAS), disrupting the conserved phosphopantetheinylation motif. AI-9 represents an informative exception: it contains asparagine, an often-occurring residue at this position, and was nevertheless non-functional. Together, these observations from the ProteinMPNN variants directly motivated the FFxxGGxS motif-retention filter introduced in Round 2.

Importantly, non-functional variants were not excluded from subsequent analyses. All sequence–activity outcomes, including inactive designs, were retained as negative labels during training of the surrogate models used in Round 3.

We revised the manuscript accordingly. In the Results section (page 4-5), the Round 1 selection procedure now explicitly states that candidates were selected to represent both generative models and to span the phosphopantetheinylation core-motif diversity of the generated designs, without additional *in silico* filtering. In addition, **Supplementary Fig. S17** now shows the ESM2 embedding of the generated pool, confirming that the selected variants are distributed across the ESM2 embedding space across both models, and **Supplementary Note 1** has been expanded to describe the complete Round 1 selection workflow.

3. In Figure 3, WT activity fluctuates 17-fold across different media. Why was "% WT activity" reported under non-standardized conditions? Was analysis of covariance performed to rule out culture condition interference with interfacial effect conclusions?

In Figure 3, the y-axis reports absolute production titers (mg L^{-1}), not % WT activity; the ~17-fold difference the reviewer refers to is the effect of culture medium on WT titer (~2.9 mg L^{-1} in succinate XPP versus ~50 mg L^{-1} in LB), which we deliberately characterised in this experiment (Fig. 3b) to quantify the condition dependence of the readout. Where percentages relative to WT are quoted in the text, they are always normalised to a WT control grown in the same experiment under identical conditions (WT = 100 %), so these values are not derived across non-standardised conditions. Importantly, our conclusions about interfacial and variant-specific effects do not rely on comparisons across media. Within every experiment, all variants and the WT control were cultured under identical conditions and normalised to that internal WT control; the medium- and scale-comparison in Fig. 3b was a separate, deliberate robustness assessment rather than the basis for any interfacial conclusion. This is stated explicitly in the manuscript (Results, page 7; Fig. 3): *"…absolute titers, and in some cases relative differences between variants, depend strongly on cultivation conditions. Accordingly, production titers should be interpreted as condition-dependent functional labels rather than context-independent measures of sequence performance. These findings highlight the need for standardized conditions to ensure internal consistency across DBTL rounds."* For these reasons we did not apply analysis of covariance: the experiments were not designed as a factorial variant x medium screen but as matched, WT-normalised comparisons within fixed conditions, which control for cultivation effects by design rather than post hoc.

4. What is the statistical basis for the "contact reorganization" conclusion in MD simulations? Please provide significance tests (e.g., t-test p-values) for contact occupancy differences, the number of simulation replicates, and discuss reliability risks associated with templates showing 22–35% sequence identity.

We thank the reviewer for this important comment and have added the requested information. Each catalytic state and variant was simulated in duplicate (two independent 100-ns trajectories per system), now stated explicitly in the Methods (page 20): "*For each tri-domain system and catalytic state, two independent 100-ns simulations were performed*." Replicate-resolved residue–residue contact-persistence statistics for all systems are provided in Supplementary Table S3 ("*Quantification of interface contact persistence across catalytic states … two independent replicates each*"), allowing the consistency and magnitude of the observed contact differences to be assessed directly.

Regarding template reliability, we now report the underlying sequence identities in Supplementary Table S4: full-construct identities range from 28.4 % to 34.8 % and domain-level identities from 19.5 % to 44.1 %. We agree that homology models at this level of identity carry uncertainty in absolute inter-domain geometry, and we therefore restrict our interpretation to relative, within-framework comparisons between WT GxpS_T3 and AI-2 rather than to absolute structural accuracy. This is now explicit in the Methods (page 20): *"…the MD simulations were used to compare relative conformational stability, dynamic behavior, and interdomain interaction trends between tri-domain constructs containing either WT GxpS_T3 or AI-2, rather than to infer atomistic mechanistic detail or absolute structural accuracy…*"

On formal significance testing: with two independent replicates per system, per-contact t-tests would rest on n = 2 and would yield unreliable p-values, so we deliberately refrained from reporting them to avoid conveying a false impression of statistical power. Instead, we report the full per-replicate contact-persistence values (Supplementary Table S3) so that the reproducibility and effect size of the differences can be judged transparently.

To reflect these limitations, we have softened the language throughout the MD section so that it is explicitly comparative and hypothesis-generating, for example (Results, page 9): *"…comparative contact analysis suggested state-dependent differences in interdomain interaction organization…"* and "*Together, these simulations suggest that AI-2 preserves global fold stability while altering the distribution, persistence and chemical nature of interdomain contacts in a state-dependent manner*." The central conclusions of the study rest on the experimental data, with the MD analysis presented as a supporting, mechanistic hypothesis.

5. For the LC-MS data of the hybrid NRPS system, why are only relative peak areas reported? Please explain the mechanistic hypothesis for why AI-12 succeeded in GshS (Figure 7b) but failed in GxhS-1 (Figure 7a). Was interference from upstream A-domain interactions considered?

Regarding the use of relative peak areas: for the hybrid products (compounds 9–26) authentic synthetic standards were not available, so absolute quantification by external

calibration — as used for compounds 1, 2 and 6 — was not possible. We therefore report extracted-ion peak areas as a semi-quantitative measure of relative product abundance. Crucially, every comparison is made only between variants producing the same peptide, each relative to the corresponding native-T-domain control; we do not compare peak areas between different peptide products, whose ionization efficiencies may differ. We have clarified this within-peptide basis in the revised Results (page 12): "*Because peak areas for a given peptide were compared under matched LC-MS conditions, these differences reflect relative changes in its production*" and made it fully explicit in the Methods (page 18): "*Peak areas were integrated from extracted-ion chromatograms (EICs) and used for relative comparison between samples producing the same peptide product; peak areas were not compared between different peptides, and no absolute quantification was performed*"; it was already indicated in the Fig. 7 legend: "*LC-MS peak areas were used as a semi-quantitative measure of relative peptide abundance across matched experimental conditions*". Accordingly, all comparisons in Fig. 7 are made within a single product species relative to the corresponding native-T-domain control, never across different peptides.

Concerning the differential behavior of AI-12, both GxhS-1 and GshS retain the same GxpS A-domain upstream of the transplanted T-domain (in both hybrids the GxpS A-domain and the transplanted T-domain are joined to a partner-derived downstream module). Upstream A-domain interactions therefore cannot account for the difference. The two hybrids differ downstream: the transplanted T-domain is followed by a C/E-type domain in both cases — the primary determinant of compatibility in our dataset — but by different specific C/E domains, derived from XtpS (GxhS-1) versus SzeS (GshS). AI-12 thus appears compatible with the SzeS-derived C/E junction (~6.5 x 10^6 peak area, ~65 % above native Sze_T4) but not with the XtpS-derived one (no detectable product). We do not currently have a definitive mechanistic explanation for this single-variant observation and are cautious not to over-interpret it: the other variants do not track AI-12 — for example, AI-27 increased production in GxhS-1 but was reduced in GshS, the opposite pattern — indicating that individual-variant outcomes are partner-specific and not fully predictable. The robust, generalizable signal in our data is therefore at the level of the downstream C-domain class rather than the individual T-domain x specific-partner combination. We have accordingly refrained from asserting a specific mechanism for the AI-12 case and present it as an example of the variant-specific context dependence that motivates our screening-based strategy and our framing of AI-designed T-domains as context-specific rather than universally transferable parts (see also our response to Reviewer 2 on transferability).

6. The Tm value of AI-2 (~52°C) is significantly higher than that of WT (~40°C), but WT was denatured, purified, and refolded. Has consistency between refolded WT

(e.g., SEC-refolded) and native WT been validated? Does the enhanced thermal stability fail at non-T3 positions (e.g., T1/T2)?
The native WT GxpS_T3 could not be obtained in soluble form — the protein partitioned entirely into the insoluble fraction after lysis (Fig. 4b) — so a direct native-versus-refolded comparison of WT is experimentally impossible. To keep the comparison fair, the WT Tm in Fig. 4d was measured on WT purified under denaturing conditions and refolded, and AI-2 was subjected to the same denaturation/refolding treatment, so that the ~12 °C difference reflects like-treated material rather than a native-versus-refolded artifact. Importantly, the requested consistency check can be provided for AI-2, the protein that can be obtained natively: Fig. 4d reports Tm for both untreated (natively purified) AI-2 and urea-denatured/refolded AI-2, and AI-2 recovers a native SEC profile after refolding (Supplementary Fig. S5b). Untreated and urea-refolded AI-2 gave essentially identical melting temperatures (~52 °C in both cases), indicating that the refolding procedure itself does not distort the measured thermal stability. WT, by contrast, largely aggregated upon refolding and recovered only a minor native-like fraction (Supplementary Fig. S5a) — a difference in refolding behaviour that is itself part of the developability contrast we report.

Regarding other positions: thermal stability was characterised for the isolated T-domain and for multi-domain T3-context constructs (isolated T3, A3–T3, T3–C/E4 and A3–T3–C/E4; Fig. 4, Supplementary Fig. S6), and the improved developability of AI-2 was consistent across these multi-domain constructs (Results, page 8: “Similar trends were observed for multi-domain constructs containing AI-2, which consistently showed improved expression and solubility relative to WT counterparts”). We did not measure thermal stability for the designs when transplanted to other positions (e.g., T1/T2). We would note that intrinsic domain thermal stability is a biophysical property of the folded T-domain, whereas the position-dependent behaviour observed *in vivo* (Fig. 6) is governed by interface compatibility with the neighbouring catalytic domains; these are distinct properties, and the positional experiments in Fig. 6 assess the latter. A systematic biophysical comparison across positions would be an informative extension.

7. The study focuses on the GxpS system, leaving broader applicability unclear. Are the "downstream C-domain compatibility rules" (LCL-type vs. C/E) generalizable to other NRPS families? Testing in a distantly related NRPS (e.g., from Streptomyces or fungi) would strengthen claims of "context-conditioned optimization." Additionally, the "positional dependence" (Fig. 6) shows variable performance across T1–T5 positions, but the structural basis (e.g., sequence motifs unique to T5) is not mechanistically explained.

We agree that generality across NRPS families is an important question, and we do not claim that the ${}^{L}C_{L}$-type versus C/E-type preferences are universally applicable across all NRPS families. We would emphasise, however, that the downstream-C-domain-class

dependence we exploit is already supported well beyond the single GxpS system, on two levels. Experimentally, the rule held across three non-cognate biosynthetic systems (GxpS, XtpS and SzeS), their hybrids, and the native SzeS assembly line, where compatibility was "*primarily conditioned by the class of the immediate downstream C-domain ($^{L}C_{L}$ -type versus C/E)*" (Results, page 14; Fig. 7). At the sequence level, our phylogenetic analysis (Supplementary Fig. S10) already spans a taxonomically broad set of natural NRPS T-domains — including *Pseudomonas, Bacillus, Burkholderia* and myxobacterial (e.g., *Myxococcus, Corallococcus*) sequences in addition to *Xenorhabdus* and *Photorhabdus* — and shows that AI-designed and natural T-domains partition according to downstream-partner identity across these lineages. This is further corroborated by our previous large-scale analysis of 130,870 natural bacterial T-domains, in which the residue preceding the phosphopantetheinylation serine covaries with the class of the downstream C-domain (**ref. 39**). Together, these analyses indicate that the downstream-partner dependence is a general property of natural T-domains rather than a peculiarity of GxpS. We fully agree that direct experimental validation of de novo T-domain design in a phylogenetically distant assembly line (e.g., from Streptomyces or fungi) would further strengthen this conclusion and is a clear next step; such experiments are beyond the scope of the present proof-of-concept study.

Regarding the positional dependence in Fig. 6, we do not identify a T5-specific sequence motif, and we prefer not to over-interpret these data, which are based on three variants. Our designs were generated in the T3 (C/E) context, and the $^{L}C_{L}$ -type position T2 was restrictive; however, the pattern is not a simple function of downstream C-domain class, since position T1 (also C/E) was likewise incompatible with AI-2 and AI-12, whereas the terminal position T5 (followed by a thioesterase rather than a C-domain) was highly permissive, and individual variants differed in their positional preferences. The mechanistic basis of these position-specific effects is not resolved by our current data, and we refrain from speculating beyond noting that they likely reflect the specific local context at each junction; a residue-level structural and biochemical account would be required and is an interesting direction for future work. We note that our conclusion — that the class of the immediate downstream C-domain is the dominant determinant of compatibility — is drawn from the larger hybrid screen (Fig. 7) rather than from this positional experiment.

Reviewer #2 (Remarks to the Author):

The manuscript submitted by Bülbül and colleagues combines generative protein design approaches (ESM3, ProteinMPNN, EvoDiff) with iterative design cycles to generate de novo thiolation (T) domains for non-ribosomal peptide synthetases (NRPSs). They experimentally evaluate a large number of variants, showing that a

substantial fraction of AI-designed T-domains support peptide production and can, in some cases, outperform native domains. I consider that the topic and experimental scope is well chosen and is of interest for Nat Commun readers. However, some key aspects require clarification or strengthening, all of them listed here without any specific order of priority:

We thank Reviewer 2 for the positive and constructive assessment of our manuscript, and we address each point raised below.

1. While the manuscript emphasizes context-aware generative design, the actual pipeline appears closer to generate → filter → experimentally screen than to predictive, mechanism-driven design. For me, there is no direct evidence that the models a priori encode or optimize interdomain interface features. I consider that the authors could provide in silico evidence that selected designs are enriched in interface-relevant featuress.

We agree that our study does not show that the generative models explicitly optimize interdomain interfaces or that interface compatibility can be predicted a priori from model outputs alone, and we have moderated the manuscript accordingly. Our claim is narrower: T-domains were generated conditionally within their intended A–T–C neighbourhood — the flanking A- and C-domains retained as sequence and/or structural context, the T-domain masked and regenerated — so the models had access to the surrounding catalytic context, and the resulting designs are functional in that context.

We also agree that *in silico* evidence for enrichment of interface-relevant features is valuable, and such evidence is in fact already present in the manuscript. In the phylogenetic analysis of AI-designed and natural T-domains annotated by downstream partner (Supplementary Fig. S10), the designs segregate by downstream-partner identity: AI-1–AI-66 (generated in the C/E context) cluster with C/E-associated natural T-domains, whereas AI-67–AI-76 (generated for an LCL-type context) cluster with LCL-associated T-domains. This separation is most pronounced in Tree 2, which is built from the downstream-facing region of the T-domain — the segment contacting the downstream C-domain — indicating that the designs are enriched in sequence features associated with their intended interface, consistent with the established covariation between downstream-facing T-domain features and downstream C-domain identity across natural T-domains (**ref. 39**).

Consistent with this, compatibility tracks neighbouring-domain identity *in vivo*: T-domains generated for LCL-type interfaces (AI-67–AI-76) supported activity with LCL-type C-domains but were inactive with downstream C/E-domains, whereas designs generated in the native C/E context showed the opposite preference (Fig. 7).

We have therefore expanded the Discussion and moderated language that could be interpreted as implying direct interface optimization by the underlying models as follows (page 15): "*More broadly, our results show that generative design can be applied to dynamic megasynth(et)ases through engineering of carrier domains that contribute to interdomain communication during catalysis. Importantly, the present study does not demonstrate that the underlying generative models explicitly learn or optimize interdomain interfaces. Rather, the design strategy conditions sequence generation on the surrounding A–T–C context, allowing the models to access information associated with the intended catalytic neighborhood during sequence generation. The observed context dependence therefore reflects successful context-conditioned generation rather than direct evidence of explicit interface optimization. This interpretation is supported most directly by the differential behavior of T-domains designed for LCL-type versus C/E-type downstream C-domains — designs generated for an LCL-type context supported activity only with $^{L}C_{L}$-type partners, whereas C/E-context designs showed the reciprocal preference (Fig. 7) — and by the phylogenetic segregation of AI-designed T-domains by downstream-partner identity, most pronounced in the downstream-facing region of the domain (Supplementary Fig. S10). The pronounced positional dependence within GxpS (Fig. 6) is consistent with this picture and further indicates that the designs behave as context-specific rather than position-independent parts.* "

2. I recognize the important number of variants that were tested but it is unclear for example how many sequences were initially generated per round, what fraction was filtered out prior to experimental testing, whether selection criteria introduce bias toward sequences already close to functional? This makes it difficult to assess the true success rate of the approach.

The per-round numbers are consolidated in Supplementary Note 1 ("Candidate selection across design rounds"; see also our response to Reviewer 1 point 2): Round 1 — 200 sequences generated (100 each with ESM3 and ProteinMPNN), 11 tested (AI-1–AI-11) with no activity- or plausibility-based filtering; Round 2 — 1,200 sequences generated (400 from each of three generation methods: ESM3 with structural conditioning, ESM3 without structural conditioning, and EvoDiff), reduced by motif-retention (FFxxGGxS), ≥50 % identity to WT and ESM2-perplexity filters plus embedding-based clustering to the tested subset (AI-12–AI-36); Round 3 — ~5,000 sequences generated, prioritized by a surrogate model trained on the Round 1–2 activity data (AI-37–AI-66).

The reviewer is correct that the later rounds enrich for functional candidates; this is by design, and the manuscript reports the resulting increase in the functional fraction across rounds (Fig. 2b,c). It does not, however, prevent assessment of the true success rate: Round 1 was deliberately unfiltered and therefore gives the unbiased, out-of-the-box success rate. Under a detectable-product criterion, 6 of the 11 Round 1 designs (~55 %)

were functional, and this performance was strongly model-dependent — 4 of 4 ESM3 designs versus 2 of 7 ProteinMPNN designs. Rounds 2–3 are explicit enrichment steps: the Round 2 filters are generic sequence-plausibility criteria (motif retention, a minimum identity threshold and perplexity) rather than activity predictors, and only Round 3 uses an activity-trained surrogate model. Because sequence identity to WT had little predictive value for activity in our data (Supplementary Fig. S3), even the identity filter does not presuppose function. Round 1 therefore provides the naive baseline against which the enrichment in later rounds should be interpreted.

3. The improved developability of AI-2 (higher soluble expression, efficient refolding, and also higher Tm) is compelling. However I think that the analysis is limited to a single design and it is unclear whether this improvement is representative of AI-designed sequences correlated with functional output. Given the central importance of expression and solubility in NRPS engineering, this is a key gap. I don't know if the authors analyzed a possible correlation between expression/solubility vs thermal stability?

We agree that our quantitative biophysical characterization of developability (expression, solubility, refolding and thermal stability) was performed for a single design, AI-2. We therefore present AI-2 as a proof-of-concept that generative design can yield a more tractable T-domain scaffold than WT GxpS_T3, and we do not claim that all AI designs share this property; because these biophysical data exist only for AI-2 (n = 1), a correlation between expression/solubility and thermal stability across designs cannot be computed. A systematic developability panel across multiple designs, together with its correlation to functional output, is a worthwhile next step.

4. Stability claims are primarily based on thermal unfolding (Tm) and SEC-based refolding behavior. While informative, these measurements do not fully capture protein stability. Given that stability is used to support mechanistic interpretations, the current level of characterization is somewhat limited and might be rephrased to make clear that the experimental approach is not supporting completely a whole conformational stability analysis.

We agree these measurements do not fully capture conformational stability. Tm (nanoDSF) and SEC-monitored refolding were used to compare the relative biophysical properties of WT GxpS_T3 and AI-2 under matched conditions, not to map their full thermodynamic or conformational stability landscapes.

We have therefore revised the manuscript to clarify that these measurements assess relative developability and biochemical robustness of the variants under the tested

conditions and should not be interpreted as a comprehensive analysis of global conformational stability as follows (page 8): "*Thermal denaturation further indicated enhanced thermal robustness of AI-2 (Tm ~52 °C) compared with refolded WT GxpS_T3 (Tm ~40 °C) (Fig. 4d). … . Together, these data provide a relative, operational comparison of the foldability and biochemical robustness of WT GxpS_T3 and AI-2 rather than a comprehensive measure of their global thermodynamic or conformational stability."*

5. MD simulations suggest differences in interdomain contact networks between WT and AI-2. However these observations remain correlative and no experimental validation is provided (eg. mutational testing)… Thus, the mechanistic conclusions drawn from MD are suggestive but not strongly supported. Although experimentally tested key MD-derived hypotheses (e.g., targeted mutations disrupting predicted contacts) would be a lot informative, I know that's a lot of work and maybe a different manuscript, so I suggest tone down mechanistic claims.

We agree, and have toned down the mechanistic interpretation of the MD results throughout the manuscript so that they read as hypothesis-generating rather than as a demonstrated mechanism. The simulations were intended to compare WT GxpS_T3 and AI-2 across catalytic states and to flag possible interdomain-interaction differences that might underlie the observed context dependence, not to prove a mechanism.

The Results now use deliberately cautious wording, e.g. *(page 9): "…comparative contact analysis suggested state-dependent differences in interdomain interaction organization…"* and *"Together, these simulations suggest that AI-2 preserves global fold stability while altering the distribution, persistence and chemical nature of interdomain contacts in a state-dependent manner."*

We have also added an explicit caveat to the Discussion (page 15):

"However, the MD-derived interaction patterns should be viewed as hypothesis-generating, and future studies combining targeted mutagenesis with biochemical and structural characterization will be required to establish causal relationships between specific interdomain contacts and NRPS activity."

As the reviewer notes, direct validation — e.g., targeted mutagenesis of predicted contacts — would be informative but is beyond the scope of this study, which centres on demonstrating context-conditioned generative design of functional T-domains; we now flag such experiments as the necessary next step to establish causal contact–activity relationships.

6. Clarify the operational definition of "functional" (detectable vs above threshold).

We thank the reviewer for this suggestion. We have now explicitly defined functional T-domains in the Results section (page 4) as follows: *"Throughout this study, T-domain variants were classified as functional if they supported detectable production of the expected peptide product under the corresponding assay conditions. Functional variants producing <50% of WT levels were classified as low-activity variants, those producing ≥ 50% but <80% of WT levels were classified as moderate-activity variants, and those producing ≥80% of WT levels were classified as high-activity variants."*

7. I think that the term "context-conditioned design" may be overstated and could be moderated.

We take the reviewer's point and have made the intended meaning of the term explicit rather than dropping it, since generation was in fact conditioned on the surrounding NRPS context: in every workflow the flanking A- and C-domains were provided as fixed sequence and/or structural context while only the T-domain was masked and regenerated (Supplementary Note 1). We use "context-conditioned" in this specific, procedural sense.

To prevent over-reading, the term is now defined operationally at first use in the Results (page 3): *"…an explicit local-context constraint: we specified the intended A–T–C neighborhood by providing the amino acid sequences of the flanking A and C domains … while masking the T-domain segment during generation…"*, and the Discussion explicitly bounds it (page 15): *"the present study does not demonstrate that the underlying generative models explicitly learn or optimize interdomain interfaces … The observed context dependence therefore reflects successful context-conditioned generation rather than direct evidence of explicit interface optimization"*.

The following additional changes have been introduced into the manuscript:

- Results (page 12): We clarified that the hybrid NRPS experiments evaluate T-domains generated in a different catalytic context rather than by explicit interface optimization: *"To test context-conditioned design for a different C-domain class, T-domains generated in an LCL-type C-domain context were evaluated in a second GxpS–XtpS hybrid (GxhS-2), in which GxpS_T2 was fused upstream of the C3-domain of XtpS to position the engineered carrier directly before an LCL-type C-domain."*

- Supplementary Note 1 (pages 25–26): We expanded the sections "Library generation using generative protein models" and "Candidate selection across design rounds" to provide a detailed description of the sequence- and structure-conditioned generation workflow, how the local A–T–C context was incorporated during AI sequence generation, and the candidate-selection strategy used across the three DBTL rounds.

8. I’m not an expert in LC-MS/MS, but since the manuscript relies heavily on this, could the authors clarify how variability in ionization efficiency across different peptide products is controlled for? In particular, how robust are comparisons across variants producing structurally related but non-identical peptides?

For compounds 1, 2 and 6, ionization variability was controlled using authentic standards and external calibration curves, allowing absolute quantification. The hybrid-system products (9–26) had no authentic standards available and were therefore assessed semi-quantitatively from extracted-ion peak areas under matched LC-MS conditions; crucially, peak areas were compared only between variants producing the same peptide, each against its native-T-domain control, never between different peptide structures, which can differ in ionization efficiency. Because we make no cross-peptide comparisons, differences in ionization efficiency between structurally distinct peptides do not enter any comparison or conclusion. This within-peptide, semi-quantitative basis is stated in the revised Results (page 12): "*Because peak areas for a given peptide were compared under matched LC-MS conditions, these differences reflect relative changes in its production*", made explicit in the Methods (page 18) *"…used for relative comparison between samples producing the same peptide product; peak areas were not compared between different peptides, and no absolute quantification was performed",* and was already indicated in the Fig. 7 legend ("*a semi-quantitative measure of relative peptide abundance across matched experimental conditions*").

9. The authors state that round 1 designs were evaluated “out-of-the-box” without in silico filtering beyond sequence clustering and representative sampling. It would be helpful to clarify what criteria were used for clustering and selection of representative sequences. In particular, what sequence identity thresholds or clustering algorithms were applied? How many sequences were initially generated, and how many were retained after clustering? I was wondering if this step could have implicitly biased the selection toward more “natural-like” or higher-likelihood sequences? This is particularly relevant for interpreting the apparent success of ESM3 designs in round 1, as even minimal preprocessing may substantially enrich for functional sequences relative to a fully unfiltered generative output.

We agree this is important for interpreting the Round 1 success rates and have clarified the complete selection procedure in Supplementary Note 1 ("Candidate selection across design rounds"). In Round 1, we generated 200 sequences (100 each with ESM3 and ProteinMPNN). From this pool we selected 11 candidates (AI-1–AI-11; four ESM3- and seven ProteinMPNN-derived) by manual inspection, applying no sequence-identity threshold, perplexity filtering, structural ranking, activity prediction, or surrogate-model guidance. Selection was guided by representation of both generative models and

coverage of the phosphopantetheinylation core-motif diversity of the generated designs: ESM3 designs uniformly retained the canonical His–Ser motif, whereas ProteinMPNN designs varied the residue preceding the catalytic serine, and we selected one ProteinMPNN design for most of the distinct core-motif variants, representing the majority of the observed variation. To visualize the diversity of the generated sequence space, we have added an ESM2 embedding of all 200 generated sequences together with k-means clustering and PCA/UMAP projections as **Supplementary Fig. S17**.

Importantly, the purpose of this selection was to sample the generated sequence space broadly rather than to enrich for functional candidates. Consequently, the Round 1 workflow should be viewed as an unbiased exploratory evaluation of the two generative models rather than a filtered optimization procedure (see also our response to Reviewer 1, point 2).

10. The authors extend their analysis by testing whether AI-designed T-domains can function as interchangeable parts in hybrid NRPS systems, which is an important and practically relevant question. Maybe I'm wrong but the observed outcomes appear highly context-dependent, with strong variation across hybrid systems and downstream partner domains. This raises the question of whether the current approach enables predictive engineering, or whether it still relies on empirical screening of multiple candidates.

We agree, and would characterise the current approach honestly as enrichment-based rather than fully deterministic: conditioning generation on the intended NRPS context substantially raises the frequency of functional T-domains and reduces the experimental search space, but it does not predict which specific variant will suit a specific partner, so screening of a candidate set is still required. Our data are, however, predictive at a coarser level — the class of the immediate downstream C-domain (LCL-type versus C/E-type) reliably determines compatibility — while remaining screening-based at the level of the individual variant.

The hybrid screen shows both aspects. Across architectures the success rate was high but strongly context-dependent: in C/E-type contexts most functional AI-designed T-domains retained high activity (GxhS-1: 53 high-activity, 2 moderate-activity, and 19 non-functional variants; GshS: 33 high-activity, 2 moderate-activity, 32 low-activity, and 9 non-functional variants). By contrast, in the LCL-type context (GxhS-2), only five variants supported detectable product formation (2 high-activity and 3 low-activity variants, with 71 non-functional variants), and the LCL-context designs (AI-67–AI-76) were functional only in this LCL-type context. This combination of class-level predictability and variant-level context dependence is what one expects from context-conditioned enrichment, and

we present it as a step toward more predictive NRPS engineering rather than as fully predictive design.

We have accordingly expanded the Discussion (page 15) to distinguish context-conditioned generation from fully predictive engineering and to frame the workflow as reducing, not eliminating, experimental validation: "Taken together, our data argue for complementing post hoc screening of hybrids with the design of carrier domains for the intended local catalytic neighborhood — reducing, rather than eliminating, the experimental search — as a route to context-conditioned NRPS engineering." The Discussion further outlines how partner-aware predictors trained on expanding sequence–context–function datasets "should progressively reduce reliance on iterative DBTL and enable increasingly prospective NRPS engineering."

11. In this regard, for me it is not entirely clear how transferable individual designs are across different NRPS architectures; for example: do top-performing variants in one hybrid context tend to perform well in others? I think the manuscript would benefit from a more explicit discussion of the limitations of using AI-designed domains as modular engineering parts. For example, in particular to what extent can these domains be considered reusable components versus context-specific solutions that require re-optimization for each new assembly line?

Our data show that individual designs are largely context-specific: strong performance in one architecture does not reliably transfer to another. Among the top SU2 performers tested in both C/E-type hybrids, AI-2 improved production in both GxhS-1 and GshS, whereas AI-27 improved GxhS-1 but fell below the native reference in GshS, and AI-12 showed the opposite pattern (inactive in GxhS-1, best-performing in GshS); the LCL-context designs (AI-67–AI-76) were likewise active only with LCL-type partners. Even top-ranked variants therefore do not transfer uniformly, and we regard AI-designed T-domains as context-conditioned solutions rather than universally reusable modular parts — although, because many designs do function across non-cognate architectures, generative design still raises the likelihood of finding compatible carrier domains and narrows the screening space.

The reviewer is right that the manuscript should state this limitation explicitly, and we have revised the Discussion (page 15) accordingly. It now makes the point on two levels: first, that "*Interface compatibility is inherently context dependent, so the goal is not universal interchangeability but reliable, junction-conditioned design*"; and, added in revision, that "*the top-performing designs did not transfer uniformly between hybrid architectures (Fig. 7), so AI-designed T-domains are best regarded as context-specific solutions that may require re-optimization for each new assembly-line context rather than as universally reusable modular parts.*"

12. The authors use mean pLDDT scores from ESMFold as a proxy for foldability of generated sequences. While pLDDT is a useful indicator of model confidence in predicted structures, its interpretation as a direct measure of protein foldability or stability warrants further clarification. Could the authors clarify how well mean pLDDT correlates with actual folding, solubility, or stability in their dataset? It would strengthen the manuscript to include an analysis of whether pLDDT scores correlate with experimental outcomes.

We agree pLDDT should not be read as a direct measure of foldability, stability, solubility, or activity, and we now treat it explicitly as a structure-prediction confidence metric (Suppementary note 1, page 27). As requested, we tested whether it predicts experimental outcome: across all 66 AI-designed T-domains, mean ESMFold pLDDT showed no significant correlation with production (Pearson $r = -0.18$, $p = 0.14$; Spearman $\rho = -0.02$, $p = 0.88$; new Supplementary Fig. S15). Panel-wide solubility and thermal-stability data are not available (these were measured only for AI-2), so production — quantified for all 66 variants — served as the functional readout. This confirms that pLDDT is not predictive of experimental performance here and justifies its use solely as a confidence metric.

13. Additionally, using the mean pLDDT across the entire sequence may obscure local regions of low confidence (maybe interface-relevant residues or flexible loops). Have the authors considered residue-level or region-specific analyses, particularly at interdomain interfaces?

We agree that mean pLDDT can mask locally low-confidence regions. However, pLDDT served only as a coarse prioritization metric during generation and was not used to infer interface properties, interdomain compatibility, or any mechanistic conclusion — those rest on experimental validation and the MD analysis. Given that mean pLDDT does not even correlate with experimental outcome (Supplementary Fig. S15; see previous point), a residue-level analysis would not alter any conclusion, and we therefore did not perform one. The intended, limited use of pLDDT is now stated in the Supplementary note 1 (page 27): “the mean pLDDT was calculated as an indicator of structural prediction confidence and compatibility with the expected T-domain fold.”

Reviewer #2 (Remarks on code availability):

14. I did not independently install or execute the code as part of this review. However, based on the description provided in the manuscript, the computational workflow appears to be reasonably well outlined at a conceptual level

We thank the reviewer for assessing the computational workflow. All custom code for generative design, surrogate modeling, candidate prioritization and downstream analyses is publicly available at https://github.com/uds-lsv/T-domain-design (Code Availability),

with a README describing installation and usage; all third-party software and parameters are specified in the Methods and Supplementary Methods. We have also completed the journal's code and software submission checklist and are happy to provide any further detail or example data needed to reproduce the analyses.

Reviewer #3 (Remarks to the Author):

In their article 'Generative AI designs functional thiolation domains for reprogramming nonribosomal peptide synthetases', the authors prepared novel thiolation (T) domains with the aid of AI and design-build-test-learn cycles. In the best cases, these non-natural carrier protein domains exhibited better solubility and stability and enabled higher non-ribosomal peptide product yields than their native counterparts.

The authors' system of interest, Type S GxpS, consists of two independently expressed NRPS subunits (domains A1-C3 and domains A3-TE5) that interact via synthetic zippers. Functional interaction between the subunits allows for production of the cyclic compounds 5-6 (primarily 6), whereas a lack of interaction only yields the linear compounds 1-4 (primarily 1 + 2), which are detectable by LC-MS/MS. Variants of domain T3 were inserted into subunit 2 using a Golden Gate strategy. These variants were designed using three generative models (ESM3, EvoDiff and ProteinMPNN), with the neighbouring A4 and C3 domain sequences and/or structures as input and the T3 domain masked.

The authors begam with a minimal (subunit 2-only) NRPS. In the three rounds of testing, the authors analyzed progressively more T3 domain variants for production of 1 and 2. In round 1, only product titer was followed. In round 2, phosphopantetheinylation was ensured by preserving the conserved FFxxGGxS motif, a minimum identify of 50% to wild-type T3 domain (WT GxpS_T3) was imposed, and low perplexity was filtered for. In round 3, sequence-activity measurements from rounds 1 and 2 guided the selection of further candidates. Across the three rounds, the number of variants with moderate activity threshold (≥50 %) increased. The authors found a poor correlation between sequence identity to WT GxpS_T3 and product yield.

Following round 2, error-prone PCR was used to expand the sequence space of three AI-generated T3 domains (AI2, AI-12 and AI15) and WT GxpS_T3, with production of 1 and 2 as readout. Here, the AI-2 scaffold was shown to outperform the others significantly.

Subsequently, the authors turned to the full-length (subunit 1 + subunit 2) NRPS. Here, product 6 could be generated only if T3 variants productively interact with

intramodular machinery (A3 and C/E4) as well as intermodular machinery (C3). The eight top-performing variants from the SU2-only experiments were tested and found to produce 6, including two that greatly exceeded the yield of WT. Altering the culture volume (1 to 50 mL) and medium (succinate XPP medium to LB medium) revealed that titers of AI-designed variants were strongly dependent on cultivation conditions.

Next, the authors sought to further characterize AI-2, which was a top performer in the SU2-only assay. Compared to the largely insoluble WT GxpS_T3, AI-2 was found to express solubly, with higher thermal stability, in variously-sized constructs from AI-2 alone to A3–AI-2–C/E4. While AI-2 itself did not give maximal product titers, epPCR diversification of AI-2 led to the highest-yielding variant.

MD simulations of WT GxpS_T3 and AI-2 in three catalytic states (thiolation, condensation donor, condensation acceptor) followed. In each state for both T domains, all domains remained structurally stable, as evaluated by several parameters. Interestingly, interaction interfaces showed substantial differences between WT and variant.

Three T domain variants (AI-2, AI-12, AI-38) were next tested at different positions along the NRPS, taking the place of T1, T2, T4 or T5 for production of 6. As expected, most of these constructs showed loss or abolition of yield, although position T5 was found to be fairly permissive. The authors rationalized this according to downstream C domain partner type.

The authors sought to engineer functional hybrid NRPSs containing their AI-designed T domains. These hybrids consisted of parts from GxpS and XtpS (GxpS-XtpS; GxhS-1) or GxpS and SzeS (GxpS-SzeS; GshS) and introduced non-cognate interaction interfaces. By monitoring production of hybrid compounds 9 and 16, the authors found that T domain variants significantly affected yields: for Gxhs-1, three variants improved production relative to native GxpS_T3, while two abolished production; for GshS, three variants showed improved production relative to native Sze_T4, while two showed reduced production. The authors state that, in general, the hybrids designed using the XUT strategy were more successful. Testing a second GxpS-XtpS hybrid (GxhS-2), in which the fusion site was moved from T3 to T2 (in front of an LCL domain instead of a C/E domain) with T domain variants that were optimized for this C domain type showed either no or markedly improved production of 23 relative to GxpS_T2.

Finally, the authors analyzed product formation after installing 76 AI-designed T domains into GxhS-1, GxhS-2, and GshS and the native SzeS assembly line. They

found that downstream C domain type (i.e. LCL vs. C/E) was the most crucial factor in preparing active assembly lines.

In general, I find the manuscript excellent and original. It is well written and the figures are clear. However, before the article is accepted for publication in Nature Communications, I recommend a number of points be addressed.

We thank Reviewer 3 for the careful reading and very positive assessment of our manuscript, and we address the specific recommendations point by point below.

PRIMARY COMMENTS

1. The constructs modeled in MD simulations are unclear in the results section. The authors should explicitly state which domains were present in each catalytic state tested (thiolation, condensation donor, condensation acceptor). Initially, I expected the presence of four domains: upstream C and ATC. Only upon reading the methods section did I guess that the alternative positioning of the T domain on donor vs. acceptor faces of the C domain translates to condensation donor vs. acceptor states.

We agree, and now state the domain composition of each MD model explicitly. Each catalytic state was modeled as a three-domain construct in which the T-domain engages a different partner: the thiolation and condensation-acceptor states as C3–A3–T3 (the T-domain interacting with A3, and with the acceptor face of the upstream C3, respectively), and the condensation-donor state as A3–T3–C/E4 (the T-domain at the donor face of the downstream C/E4). This resolves the donor-versus-acceptor distinction the reviewer noted.

Regarding the reviewer's expectation of a four-domain (upstream C plus A–T–C) model: we deliberately did not build a single C–A–T–C construct. Although experimental four-domain structures are available, our objective was to characterize the behavior of the engineered T-domain within each catalytic state rather than simulate the complete catalytic cycle. Accordingly, we modeled the T-domain separately in the thiolation, donor-state condensation, and acceptor-state condensation states using the corresponding tri-domain construct and the most appropriate state-specific structural template (PDB 5T3D, 9BE3, and 8JBR). In all simulations, the T-domain carried the holo phosphopantetheine (PPant) arm but no amino acid- or peptide-loaded intermediate, allowing us to focus specifically on state-dependent protein–protein interactions while reducing computational cost and maintaining a consistent comparison between WT and AI-designed T-domains.

These compositions are now stated directly in the Results (page 9) and Methods (page 19), with template identities in Supplementary Table S4: "The thiolation-state model

consisted of a C3–A3–T3 construct representing the thiolation state, in which the T-domain interacts primarily with the A3 domain. The condensation donor-state model consisted of an A3–T3–C/E4 construct representing delivery of the tethered intermediate to the donor-binding face of the downstream C/E4 domain. The condensation acceptor-state model consisted of a C3–A3–T3 construct representing positioning of the T-domain at the acceptor-binding face of the upstream C3 domain. Homology models for the thiolation, condensation donor, and condensation acceptor states were based on PDB entries PDB 5T3D, 9BE3, and 8JBR, respectively. Domain-level sequence identities between target and template structures are summarized in Supplementary Table S4.

2. I would like to see improved figures showing major domain-domain interactions in the MD simulations (currently, catalytic domains are only displayed as surfaces). These could be included as supplementary figures.

To address this, we have added a new supplementary figure (Supplementary Fig. S16) that complements Fig. 5 with residue-level views of the major interdomain interactions from the MD simulations in the thiolation, condensation-donor and condensation-acceptor states, showing representative persistent hydrogen-bond contacts between the T-domain and its catalytic partner domains. The underlying residue–residue contact statistics (per state and per replicate) are provided in Supplementary Table S3 and in Supplementary Data 1 (MDcontact_analysis).

3. In the section 'AI-designed T-domains can be leveraged to generate hybrid NRPSs', C domain type could be included in Fig. 4, or at least explicitly mentioned in the text, for easier comparison.

We thank the reviewer for this suggestion. The hybrid NRPS data are shown in Fig. 7, whose legend we have revised to define the domain symbols, including the C-domain types (page 14): "Domain symbols are as follows: A, circle; T, rectangle; C, triangle; C/E, diamond; TE, terminal circle." In addition, the downstream C-domain type is stated explicitly in the main text, where compatibility is described as governed by the class of the immediate downstream C-domain (LCL-type versus C/E). We also now state this where the hybrids are introduced (Results, page 12): *"…in both GxhS-1 and GshS the transplanted T-domain is followed by a C/E-type C-domain."*

4. Also in this section, the authors write, 'To test interface-specific design, T-domains optimized for LCL-type condensation interfaces were evaluated…'. To my knowledge, no detail is given on how these variants were generated compared to the others that were optimized for C/E type interfaces.

We thank the reviewer for prompting this clarification. These T-domains were not produced by a dedicated optimization procedure; the original wording *"optimized for … interfaces"* was misleading, as the designs were simply generated in a different sequence

context rather than optimized. AI-67–AI-76 were generated with the same conditional workflow as the other AI-designed T-domains, using ESM3 and EvoDiff conditioned on an A–T–C context comprising the GxpS A2 and XtpS C3 (LCL-type) domains with the T-domain masked; candidates were then selected exactly as in Round 2 (motif, identity and perplexity filtering, followed by ESM2-embedding clustering into five groups per generation method and selection of the lowest-perplexity representative per cluster), yielding 10 variants. This procedure is now described in Supplementary Note 1 (page 27, "Generation of LCL-context T-domains (AI-67–AI-76)"). To make explicit that no separate optimization was performed, we have replaced "*optimized/tailored for … interfaces*" with "*generated in/for a … context*" throughout the hybrid section, e.g. in the Results (page 12): "*To test context-conditioned design for a different C-domain class, T-domains generated in an LCL-type C-domain context were evaluated in a second GxpS–XtpS hybrid (GxhS-2)…*".

5. The authors state: 'Notably, improved production was observed in a hybrid assembled via an XUT-style39 insertion architecture (Fig. 7b), showing that AI-designed carrier domains can outperform established evolution-guided NRPS engineering approaches.' I am not fully convinced by this claim. For direct comparison, the authors should prepare a hybrid GxpS-XtpS NRPS using the XUT approach or a hybrid GxpS-SzeS NRPS using the same whole-domain replacement strategy as for GxhS-1.

We agree, the original wording overstated the comparison; we have removed the claim that AI-designed carriers "*outperform established evolution-guided NRPS engineering approaches*" (page 12).

We would also clarify how the hybrids were constructed, as this bears directly on the reviewer's suggestion. GshS (Fig. 7b) is a GxpS–SzeS hybrid assembled by XUT-based recombination (ref. 39), fusing GxpS to the T4–TE region of SzeS with the donor's native carrier (Sze_T4) reintroduced — *i.e.* the whole-domain, evolution-guided design the reviewer describes. GxhS-1 and GxhS-2 (Fig. 7a,c), by contrast, are GxpS–XtpS fusions built at the traditional C-A-T module boundary. The control the reviewer requests — a GxpS–SzeS hybrid built by whole-domain replacement — is therefore already present as GshS. Because the native-Sze_T4 GshS construct is precisely the evolution-guided reference, and AI-designed carriers were substituted into that identical scaffold, the native-carrier vs. AI-carrier bars in Fig. 7b constitute the direct head-to-head comparison requested: substitution increased titre of vLfYW (16) by ~25% (AI-2) and ~65% (AI-12) relative to the native carrier.

We nonetheless take the reviewer's underlying point that this is a limited demonstration — a single hybrid system in which the clearest gain comes from one variant — and have toned down the statement accordingly. The revised text now reads (page 12): " *Notably,*

GshS was built with the XUT-based approach (XUT-1[39]) and carries the native Sze_T4 domain. In this particular hybrid, replacing the native domain with AI-designed T-domains increased product formation (Fig. 7b). This is a single, construct-specific example rather than a general rule, but it shows that AI-designed carriers can improve yields relative to the native carrier installed by XUT-1."

SECONDARY COMMENTS

Main section
6. The authors have written: 'This has given rise to a rapidly expanding toolkit of generative approaches spanning several model classes. (I) Sequence-based foundation models such as ESM311; (II) Diffusion models such as EvoDiff12; and (III) Structure-conditioned design methods such as ProteinMPNN13.'

Please combine these sentences as follows:
This has given rise to a rapidly expanding toolkit of generative approaches spanning several model classes: (I) Sequence-based foundation models such as ESM311; (II) Diffusion models such as EvoDiff12; and (III) Structure-conditioned design methods such as ProteinMPNN13.

We have revised the sentence accordingly.

Results section
7. Consider rewriting 'A3–TE' as 'A3-TE5' for clarity.

We have revised the text accordingly.

8. The authors have written: 'Candidate T-domains were proposed using three complementary generative strategies (ESM311, EvoDiff12, ProteinMPNN13), under an explicit local-context constraint: we specified the intended A–T–C neighborhood by providing the flanking A and C domains (and, where applicable, structural context) while masking the T-domain segment during generation, thereby forcing designs to be compatible with the target junction environment (Supplementary Note 1; Supplementary Data 1).'

Please edit this text to state explicitly that you provided amino acid sequences; e.g., '…by providing the amino acid sequences of flanking A and C domains'.

We have revised the text to clarify that the amino acid sequences of the flanking A and C domains were provided as context during sequence generation as follows (page 3): "*we specified the intended A–T–C neighborhood by providing the amino acid sequences of the flanking A and C domains ".*

9. The following terms would benefit from definitions or simplified descriptions, as they may be foreign to the general reader (including this reviewer!): structure tokens, lightweight in silico filters, data-driven surrogate models.

We thank the reviewer for this suggestion. To improve accessibility for a broader readership, we have revised the manuscript to provide brief definitions of the terms *structure tokens*, *lightweight in silico filters*, and *data-driven surrogate models* at their first occurrence in the text: (page 3) *"ProteinMPNN designs were conditioned on backbone coordinates, ESM3 designs leveraged structure tokens (encoded representations of protein structural information used by the model), and EvoDiff provided a sequence-only baseline. As the campaign progressed, we combined generative proposals with progressive candidate prioritization, using lightweight in silico filters (motif, sequence identity, and perplexity-based filtering criteria, for details, see Supplementary Note 1) and, in later rounds, data-driven surrogate models (machine-learning models trained on experimentally measured sequence–activity data) to focus testing on sequences most likely to be functional (details in Supplementary Note 1)."*

10. Fig. 1

- In panel a, module 4 is missing a horizontal bar beneath it.

- Adding labels of SU1 and SU2 to panel a could improve clarity.

- In panel d, the text at the top should read 'heterologous expression in E. coli'

Figure 1 has been revised accordingly. A horizontal bar was added beneath module 4 in panel a, and the text in panel d was corrected to read "*heterologous expression in E. coli*". To improve clarity regarding the split assembly-line architecture, we also expanded the Figure 1 caption to explicitly describe the identities of SU1 and SU2 as follows "*SU1 comprises the upstream VL-producing portion of the assembly line, whereas SU2 comprises the downstream FLL-producing portion. Reconstitution of SU1 and SU2 yields the full-length GxpS assembly line responsible for production of the pentapeptide vLfLL.*".

11. Fig. 2

- The caption description of panel b would benefit from further explanation. While panel b is useful, not all readers will be familiar with violin plots.

We have revised the figure caption to provide a brief explanation of the violin plot representation in panel b for readers who may be unfamiliar with this visualization as follows: "*Violin plots show the distribution of normalized production levels for variants tested in each design round. The shape of the violin plot reflects the probability distribution*

of activity values, i.e. the density of variants exhibiting that level of activity, with wider regions indicating a higher frequency of observations.".

12. Fig. 4

- The x-axis labels in panel d could use clarification in the caption.

We have revised the figure caption to clarify the meaning of the x-axis labels in panel d, including the protein fractions analyzed (untreated, urea-treated, heat-soluble, and heat-insoluble samples) as follows: "*The x-axis labels indicate the analyzed protein fractions: untreated AI-2, AI-2 purified under denaturing (urea) conditions, soluble AI-2 recovered after heat treatment (heat_sup), insoluble AI-2 recovered after heat treatment (heat_pellet), and WT GxpS_T3 purified under denaturing (urea) conditions.".*

Methods section

13. The authors write: 'Separations were performed on a Hypersil GOLD C18 column (1.9 µm, 100 × 2.1 mm; Thermo Fisher Scientific) at 40 °C with a flow rate of 0.700 mL min-1 using solvent A (water supplemented with 0.1 % formic acid) and solvent B (acetonitrile supplemented with 0.1 % formic acid) The gradient for tripeptides 1 and 2 was 10 % B (0-0.2 min), 10-90 % B (0.2-0.4 min), 90 % B (0.4-1.2 min), and 10 % B (1.2-1.6 min).' A period is missing between 'formic acid) The gradient'.

The missing period has been added. Thank you for spotting this.

14. The authors write: 'Collision-induced dissociation (CID) energies were set to a basic value of 50 and tune collision energy set to star 7 eV through end 200 eV.' I believe 'star' here should be 'start'.

The error has been corrected from "star" to "start". Thank you for spotting this.

Response to the Reviewers – Final Revision

Title: Generative AI designs functional thiolation domains for reprogramming non-ribosomal peptide synthetases

Dear Reviewers,

We thank the editor and the reviewers for their continued assessment of our manuscript. We are pleased that both reviewers consider the previous revision to have addressed their concerns, and we are grateful for the suggestions raised by Reviewer 1, which we have taken up in the main text.

This revision contains no new experimental data. The changes are of two kinds: the editorial requests set out in the Author Checklist, which are answered individually in that document and are not repeated here; and one addition to the Discussion that responds to Reviewer 1. Reviewer comments are shown in bold and our responses follow in regular text.

We hope that the manuscript is now suitable for publication in Nature Communications.

Best regards,

Kenan Bozhüyük (in the name of all authors)

REVIEWER COMMENTS

Reviewer #1 (Remarks to the Author):

The authors offer comprehensive replies to all questions and specify concrete revisions to the main text and supplementary materials. They transparently acknowledge study limitations and carefully moderate overinterpretation of modeling and screening results. Most technical uncertainties raised have been satisfactorily clarified. A small number of open mechanistic questions are appropriately framed as future work.

We thank Reviewer 1 for this positive assessment and for the remaining suggestions, which we address in turn below.

Minor room for improvement remains: a few explanations rely on negative evidence or indirect support rather than dedicated control experiments;

We agree, and the manuscript is deliberately cautious on these points: the molecular dynamics results are presented as hypothesis-generating, and we state that targeted mutagenesis combined with biochemical and structural characterization will be required to establish causality. Two lines of evidence are, however, not indirect. The AI-67–AI-76 set was generated specifically for an LCL-type downstream interface, and these designs consistently gave no detectable product in the C/E-type

hybrids in which the C/E-context designs are broadly functional; in the LCL-type context the separation is real but partial, as the Results state. Independently of the activity data, the phylogenetic separation of the two design sets by downstream-partner identity (Supplementary Fig. 10) rests on sequence relatedness that was not used in generating or selecting the designs.

Finally, our claim that sequence similarity has limited predictive value concerns effect size rather than the absence of a relationship: the correlation is statistically significant but weak (Pearson r = 0.45, R^2 = 0.199, two-sided $P = 1.7 \times 10^{-4}$, n = 66), leaving roughly 80 % of the variance in activity unexplained. These statistics have been added to the legend of Supplementary Fig. 3.

some proposed future directions could be briefly highlighted within the main text rather than only in the response letter.

This is a fair observation. On checking our previous response letter we identified five directions that we had described there. Two were already stated in the Discussion: targeted mutagenesis combined with biochemical and structural characterization to establish causal contact–activity relationships, and deeper structural and kinetic interrogation of engineered junctions. The remaining three were not, and we have now added them to the closing paragraph of the Discussion:

> *"Future work should also test context-conditioned T-domain design in phylogenetically distant NRPS systems, extend biophysical characterization across larger panels of AI-designed T-domains to determine how developability relates to functional performance, and assess systematically how the choice of structural template shapes the generated sequences."*

The sentence was placed among the existing limitations in that paragraph so that the outlook which follows applies to these points as well. All five directions raised in our previous response are now stated in the main text.

Reviewer #2 (Remarks to the Author):

The authors have thoroughly addressed the concerns and general questions raised during the previous round of review. The revised manuscript incorporates the requested analyses and clarifications, and I consider that the changes have substantially strengthened the work. I have no further comments and believe that the manuscript is now suitable for publication in Nature Communications.

We thank Reviewer 2 for this assessment and for the constructive comments during the previous round, which improved the manuscript considerably. No further changes were requested and none have been made in response to this report.
